# Genome-Wide Analysis of the NAC Transcription Factor Gene Family Reveals Differential Expression Patterns and Cold-Stress Responses in the Woody Plant *Prunus mume*

**DOI:** 10.3390/genes9100494

**Published:** 2018-10-12

**Authors:** Xiaokang Zhuo, Tangchun Zheng, Zhiyong Zhang, Yichi Zhang, Liangbao Jiang, Sagheer Ahmad, Lidan Sun, Jia Wang, Tangren Cheng, Qixiang Zhang

**Affiliations:** 1 Beijing Key Laboratory of Ornamental Plants Germplasm Innovation & Molecular Breeding, National Engineering Research Center for Floriculture, Beijing Laboratory of Urban and Rural Ecological Environment, Engineering Research Center of Landscape Environment of Ministry of Education, Key Laboratory of Genetics and Breeding in Forest Trees and Ornamental Plants of Ministry of Education, School of Landscape Architecture, Beijing Forestry University, Beijing 100083, China; fjzhuoxk@126.com (X.Z.); zhengtangchun@126.com (T.Z.); zhangzhiyong543@163.com (Z.Z.); yichizhang@bjfu.edu.cn (Y.Z.); jiangliangbao@gmail.com (L.J.); sagheerhortii@gmail.com (S.A.); sunlidan@bjfu.edu.cn (L.S.); wangjia8248@163.com (J.W.); chengtangren@163.com (T.C.); 2 Beijing Advanced Innovation Center for Tree Breeding by Molecular Design, Beijing Forestry University, Beijing 100083, China

**Keywords:** NAC TFs, genetic evolution, gene duplication, expression pattern, cold response, *Prunus mume*

## Abstract

NAC transcription factors (TFs) participate in multiple biological processes, including biotic and abiotic stress responses, signal transduction and development. Cold stress can adversely impact plant growth and development, thereby limiting agricultural productivity. *Prunus mume*, an excellent horticultural crop, is widely cultivated in Asian countries. Its flower can tolerate freezing-stress in the early spring. To investigate the putative NAC genes responsible for cold-stress, we identified and analyzed 113 high-confidence *PmNAC* genes and characterized them by bioinformatics tools and expression profiles. These *PmNAC*s were clustered into 14 sub-families and distributed on eight chromosomes and scaffolds, with the highest number located on chromosome 3. Duplicated events resulted in a large gene family; 15 and 8 pairs of *PmNAC*s were the result of tandem and segmental duplicates, respectively. Moreover, three membrane-bound proteins (*PmNAC59*/*66*/*73*) and three miRNA-targeted genes (*PmNAC40*/*41*/*83*) were identified. Most *PmNAC* genes presented tissue-specific and time-specific expression patterns. Sixteen *PmNAC*s (*PmNAC11*/*19*/*20*/*23*/*41*/*48/58/74/75*/*76/78*/*79*/*85*/*86*/*103*/*111*) exhibited down-regulation during flower bud opening and are, therefore, putative candidates for dormancy and cold-tolerance. Seventeen genes (*PmNAC11*/*12*/*17*/*21*/*29*/*42*/*30*/*48*/*59*/*66*/*73*/*75*/*85*/*86*/*93*/*99*/*111*) were highly expressed in stem during winter and are putative candidates for freezing resistance. The cold-stress response pattern of 15 putative *PmNAC*s was observed under 4 °C at different treatment times. The expression of 10 genes (*PmNAC11*/*20*/*23*/*40*/*42/48*/*57*/*60*/*66*/*86*) was upregulated, while 5 genes (*PmNAC59*/*61*/*82*/*85*/*107*) were significantly inhibited. The putative candidates, thus identified, have the potential for breeding the cold-tolerant horticultural plants. This study increases our understanding of functions of the *NAC* gene family in cold tolerance, thereby potentially intensifying the molecular breeding programs of woody plants.

## 1. Introduction

Transcription factors (TFs) act as vital molecular control systems of gene expression via their specific cis-regulatory element. They can activate or repress transcriptional frequencies of their targeted genes by binding to specific promoter elements [1,2]. In plants, transcriptional regulatory networks are subtle and complex; a large part of plant genome encodes TFs genes, and more than 55 TF families have been identified, including 2296 TFs in *Arabidopsis thaliana*, 4287 in *Populus trichocarpa*, 3308 in *Zea mays* and 2780 TFs in *Prunus persica* [3]. Thus, the identification and characterization of TFs is vital to understand transcriptional regulatory mechanism. NAC (No apical meristem (NAM), *Arabidopsis* transcription activation factor (ATAF), Cup-shape cotyledon (CUC)) consists of a large and complex family of transcription factors. It is involved in multiple biological processes in plants, including perception of biotic/abiotic stress, signal transduction, transcription control and gene activation [1]. The NAC proteins harbor a highly conserved N-terminal NAC domain and a variable transcription regulatory (TR) region. The NAC domain is composed of five sub-domains (A–E) containing ~160 amino acids [4,5]. Domain A may be related to form dimer and DNA binding, and domains B and E may be involved in divergent functions, whereas domains C and D can bind to DNA [6]. The secondary structure analysis of the NACs in *Arabidopsis* and rice suggests a unique TF fold consisting of a twisted-sheet located between helical elements in N-terminus [7,8]. In addition, the NAC domain also modulates dimerization, protein-binding or DNA-binding, and nuclear shuttling or transport [1]. However, the transcription regulatory regions located in the highly divergent C-terminus are responsible for transcriptional activation or repression [7,8], and some NAC proteins sharing transmembrane motif at the C-terminal end play essential regulatory roles under environmental stress [9].

Many studies have shown that NAC proteins are widely involved in various signaling pathways [10,11], growth and development [5,12,13] and senescence [11,14,15], in response to hormones [11,16] and biotic [17,18,19,20] and abiotic stress [21,22,23]. For instance, *NAM* gene from petunia determines the positions of meristems [13]. *CUC* genes are related to the formation of a shoot-apical meristem, lateral roots, leaf margin serration and flower organ development in *Arabidopsis* [5]. Types of NAC TFs (VASCULAR-RELATED NAC-DOMAINS (VND), NAC SECONDARY WALL THICKENING PROMOTING FACTOR (NST), and SECONDARY WALL-ASSOCIATED NAC DOMAIN PROTEIN (SND)) play crucial roles in the cell wall thickening and formation of vascular vessels [24,25]. ATAF2 can bind to promoters and is involved in auxin biosynthesis [26]. FEZ and SMB control cell division [27] and *Jasmonic acid2* (*JA2*) and *JA2-like* (*JA2L*) homologs of *NAC* genes are involved in resisting the attack of pathogens [20]. The NAC family is large, involving more than a hundred genes in many plants, such as 105 *NAC* genes in *Arabidopsis* [4], 163 in poplar [28], 204 in cabbage [29], and 151 in rice [30]. However, little is known about diverse function and relationship of *NAC*s with environmental stress. 

NAC TFs may have an important contribution to plant adaptation to land [1]. Previous research revealed that a subset of *NAC*s (*NST*s, *SND*s and *VND*s) can regulate cell differentiation and promote cell wall thickening by inducing their death, which is a genetic basis of plants for water-conducting and adaptation to intricate land environment [1,24,25,31]. Recently, a novel NAC TF (*NAC87*) from oilseed rape has been reported to regulate reactive oxygen species (ROS) accumulation and promote cell death. ROS are regarded as important signaling molecules in aerobic metabolism [32]. NTM1-LIKE8 (NTL8), belonging to transmembrane NAC TF, can negate trichome formation by directly activating the expression of R3 MYB genes, *TRIPTYCHON* (*TRY*) and *TRICHOMELESS1* (*TCL1*) [33]. An S-palmitoylated MfNACsa TF protein in *Medicago falcata* can translocate to the nucleus and bind to the glyoxalase I (*MtGlyl*) promoter under drought stress [34]. In addition, NACs are also found to participate in the Abscisic acid (ABA) independent pathway of salt, cold and drought stresses [1], and ATAF subfamily acts as an import arbitrator of this process [35,36]. 

Low temperature can cause freezing of the cell membrane and oxidative stress and produces physiological and metabolic changes, resulting in irreversible damage and impact on plant growth and development [37]. In temperate climates, cold acclimation involves many plants and crops. Under cold stress, an array of cold-regulated (*COR*) genes of these plants can be induced to modify characteristics of plant physiology and biochemistry [38]. These genes can encode hydrophilic boiling-soluble polypeptides, and enhance activity of anti-oxidative enzymes and increase the accumulation of cryoprotective proteins and soluble sugars. This can improve freezing tolerance in plants by repairing membrane systems and stabilizing cellular osmotic potential [39]. For instance, 223 metabolites including carbohydrate and lipid metabolism, protein, amino and polyamines can be accumulated during cold acclimation in *Siberian spruce*, the metabolic changes help it rapidly adapt to extreme freezing tolerance [40]. The *COR* genes, mediated by multiple TFs, play a vital role in cold acclimation [39]. Over the past two decades, most studies mainly focused on the regulation of C-repeat binding factors (CBFs) and inducer of *CBF* expression1 (ICE1); CBF TFs are known as their dehydration responsive element binding factors (DREB1), which can recognize the CRT/DER cis-element and activate downstream target *COR* genes [39,41,42,43,44]. In addition, many cold-stress related cis-acting elements, including low-temperature responsive elements (LTRES) and ABA-responsive elements (ABREs), are modulated by ABA-independent pathways [45]. However, only 10–25% of *COR* genes are regulated by *CBF*s. This implies that some other TFs may be involved in cold tolerance. Recently, more attention has been paid to the NAC TFs related to cold stress [46,47,48,49]. For instance, SlNAC1 TF from *Suaeda liaotungensis* involves the ABA-dependent signaling pathway and enhances plant cold stress tolerance [49]. *MaNAC1* increases cold tolerance of banana fruit by interacting with ICE1-CBF signaling pathways [47]. *ONAC095* from rice enhances the accumulation of excess ROS and down-regulates expression of cold-responsive genes [50]. MdNAC029 can specifically bind to the promoters of *MdCBF1* and *MdCBF4*, functioning as a negative cold tolerance regulator in apple [48]. Therefore, NACs may be potentially important members in the cold-related transcriptional network. Thus far, studies about NAC TFs regulating cold tolerance remain limited.

*Prunus mume* is an excellent horticultural plant in Rosaceae, which has a long plantation history in China, and has widely been planted in Asia. This species harbors ornamental value including a fragrance, wonderful flower types and rich colors [51]. Additionally, *P. mume*’s flower buds contain multiple acylated sucrose [52]. It is well known that *P. mume* blooms earlier than most other plants in the early spring, and can tolerate −4 °C to −2 °C [53]. Thus, it is a very good material among woody plants to study mechanism of cold responses and dormancy. There are more than 300 representative *P. mume* landraces, and most of them grow well and have favorable ornamental features in southern China, where it is warm and wet (lowest temperature around 0 °C and average rainfall over 1000 mm per year) [54]. However, few landraces can tolerate extreme freezing temperature in winter and grow well in northern China (lowest temperature around −20 °C and the annual rainfalls less than 500 mm) [54], which seriously limits the popularization of excellent landraces and the production of fruit. Thus, studies have been performed to enhance the cold resistance of *P. mume* since 1957 [55]. So far, it is still unclear what potential roles of *P. mume NAC* genes are involved in cold stress. Understanding the functions and evolutions of *NAC* genes of *P. mume* could provide evidence for cold-resistance molecular breeding in future. This study presents the identification and characterization of *PmNAC*s in *P. mume* using various bioinformatics tools, including molecular characterizations, gene structure and molecular evolution and function. We also predict putative cold-stress tolerance *PmNAC*s through time- and tissue-specific expression patterns of flower bud and stem. Fifteen genes were further detected for their time-specific expression patterns under cold stress (4 °C). Our results provide potential candidate *PmNAC*s for breeding cold-resistant varieties.

## 2. Materials and Methods

### 2.1. Genome-Wide Scanning and Classification

To identify the *PmNAC* gene family in *P. mume*, the genome sequence and annotation data were obtained from the *P. mume* genome project (http: //prunusmumegenome.bjfu.edu.cn/). The Hidden Markov Model (HMM) seed profile of NAM domain (PF2365) was downloaded from the Pfam database [35] to identify NACs of *P. mume* using HMMER3 software. To ensure confidence, the E-value cut-off was set at 10^−5^. Then, all putative NAC proteins were screened to confirm the presence of the NAC domain by SMART (http://smart.embl-heidelberg.de/), and sequences without NAC domain were removed. Finally, we compared the annotated results with annotated *PmNAC* genes in *P. mume* genome. By multiple comparisons, we obtained the putative *PmNAC* genes and named them according to their physical position in the *P. mume* genome. *Arabidopsis* NAC protein sequences downloaded from TAIR 10 release (http://www.arabidopsis.org) were utilized to perform BLASTp against the PmNAC protein. In addition, the online ExPasy program (http://www.expasy.org/tools/) was used to analyze the pI (isoelectric point) and molecular weight (MW). Conserved protein motifs of the *PmNAC*s were predicted by the MEME tool (http://meme-suite.org/tools/meme). The parameters were as follows: the maximum number of motifs was set to 20 and the optimum motif width was set to 30–50. STRING 10.5 version was used to predict the interactions of the PmNAC proteins (https://string-db.org/).

### 2.2. Chromosomal Location and Gene Structure Analyses

All information of the *PmNAC* genes on chromosome was obtained from annotation documents. A schematic diagram of gene locations was drawn using an online tool (http://mg2c.iask.in/mg2c_v2.0). The gene structures of the *PmNAC*s were predicted using Gene Structure Display Server 2.0 (http://gsds.cbi.pku.edu.cn/).

### 2.3. miR164 Target Site Prediction and Membrane-Bound PmNACs Analyses

The full-length genomic sequences of *PmNAC*s were analyzed using psRNATarget online tool (http://plantgrn.noble.org/psRNATarget/), and the secondary structures were predicted using the RNAfold web server (http://rna.tbi.univie.ac.at/cgibin/RNAfold.cgi). The membrane-bound PmNAC proteins were predicted by TMHMM server v.2.0 (http://www.cbs.dtu.dk/services/TMHMM/). The hydrophobicity and tertiary structure of PmNAC protein were analyzed using ProtScale (http://www.expasy.org/protscale/) and PHYER2 Protein Fold Recognition Server (http://www.sbg.bio.ic.ac.uk/phyre2/). The sequence logo was generated using the WebLogo online (http://weblogo.threeplusone.com/).

### 2.4. Phylogenetic and Synteny Analysis

Phylogenetic trees were constructed based on maximum likelihood (ML) method using RAxML [56]. First, full-length protein sequences were performed using Mafft software with default parameters [57], trimmed using Gblocks with parameters: “conservative” mode. Then, ML phylogenetic trees, performed with optimal JTT (Jones-Taylor-Thornton) models with gamma distributed rates and tested by bootstrap of 500, were constructed using RAxML version 8 software [56]. FigTree v1.4.2 (http://tree.bio.ed.ac.uk/) was used to present the ML phylogenetic trees.

The syntenic relationship of *NAC* genes in *P.mume* and *P.persica* was conducted using Multiple Collinearity Scan Toolkit (MCScanX) [58]. Initially, potential gene pairs (E-value < 10^−5^, top 5 matches) obtained by BLASTP across *P. mume* genomes, were used as input file for MCScanX. The relationship between two species was plotted using R circlize package. Furthermore, MCScanX was also used to analyze segmental and tandem duplications, synonymous and non-synonymous rates. K_s_ values were used to calculate the dates of duplication events (T) according to the following equation: T = K_s_/2λ, where λ = 1.5 × 10^−8^ s for dicots [59,60]. The mode of selection was identified according to the standard described by Kayum using K_a_/K_s_ value [61].

### 2.5. Plant, Tissues and Stress Treatment

To understand the specific regulatory functions of the *PmNAC* genes in tissue development and cold stress tolerance of *P*. *mume*, expression patterns of *PmNAC*s were surveyed under cold stress. Initially, the expression patterns of *PmNAC*s were investigated in nine tissues (root, stem, leaf, bud, fruit and flower buds sampled in March, November, December and January) under normal growth conditions [62,63]. Moreover, expression patterns of *PmNAC* genes were analyzed in the stem of *P. mume* (‘Songchun’) at three different places (Beijing (BJ, N 39°54, E 116°28), Chifeng (CF, N 42°17, E 118°58) and Gongzhuling (ZGL, N 43°42, E 124°47)) and three periods (cold-acclimation (October, autumn), plateau period (January, winter), and deacclimation (March, spring)). Furthermore, expression profiling of *PmNAC* genes was performed for cold stress. The uniform seeds of ‘Liuban’ × ‘Fentai Chuizhi’ F1 generations natural hybrids from one individual were stored in sand with 90% humidity at 4 °C. After three months, germinated seedlings were transplanted into soil in the greenhouse. The 6-month-old seedlings were used to perform cold stress treatment under 4 °C, and around 65% humidity. Leaves of treated plants were sampled at 0, 1, 4, 6, 9, 12, 24 and 48 h for assays. All triplicate samples were frozen in liquid nitrogen, and stored at −80 °C before total RNA isolation.

### 2.6. RNA Extraction and qRT-PCR

Total RNA was isolated using RNA extraction kit (TaKaRa, Bejing, China) according to instructions. PrimeScript™ RT reagent Kit (TaKaRa) was used for the reverse transcription according to the instructions. Specific primers for qRT-PCR were designed using online tools (http://sg.idtdna.com/scitools/Applications/RealTimePCR/) and checked by Primer-BLAST tool in NCBI (https://www.ncbi.nlm.nih.gov/tools/primer-blast/) to confirm primer specificity. *PP2A* gene was used as an internal reference according to previous reports [64]. The detailed information of primers is shown in Table S1. Then, qRT-PCR was conducted using a CFX96 Real-Time PCR Detection System (Bio-Rad, Hercules, CA, USA) with SYBR Premix Ex Taq II (TaKaRa). Expression levels were calculated using the delta-delta CT method [65]. Each real-time qRT-PCR was conducted in triplicate in biological analysis.

## 3. Results

### 3.1. Identification and Annotation of PmNAC Genes

A total of 113 highly confident *NAC* genes of *P. mume* were obtained by HMMER and SMART search (Table S2), and these genes were designated as *PmNAC1*–*PmNAC113* based on the coordinate order on chromosome information following the rules for potato [66]. The N-terminal domain sequences were highly conserved, and all PmNACs contained subdomain A–D, some contained subdomain A–E, while certain PmNAC proteins contained specific domains (File S1 and Figure S2). The characteristics of NAC protein sequence greatly varied in *P. mume*. The molecular weight (10.13–161.50 kDa), sequences length (86–1429 aa), and the pI values (4.41–10.15) of 115 PmNACs are shown in Table S2.

**Figure 1:**
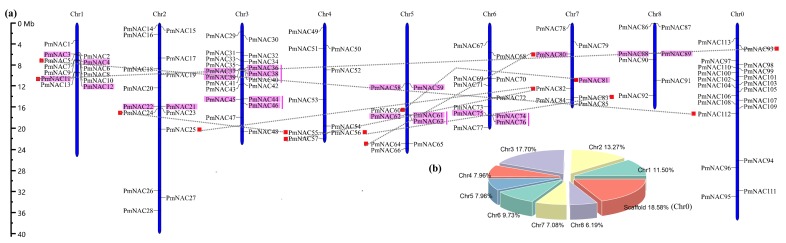
Chromosomal distribution of *PmNAC* genes on eight *Prunus mume* chromosomes. (**a**) The size of each chromosome and its corresponding *PmNAC*s distribution. All chromosomes possessed *PmNAC* genes. Chr0 indicates scaffolds; the pink rectangle refers to tandem duplicated gene pairs; genes connected with dotted grey lines refer to segmental gene pairs. (**b**) The pie chart presents the percentage of *PmNAC* genes on each chromosome.

Locations of 113 *PmNAC* genes on chromosomes were mapped, showing that 93 *PmNAC*s were located on chromosomes, and 21 *PmNAC* genes were located on scaffolds (Figure 1a). Chromosomes 1, 2 and 3 possessed 13 (~11.50%), 14 (~13.27%), and 20 (~17.70%) genes, respectively, while the proportion of *PmNAC* members on other chromosomes was less than 10%, ranging from 6.19% to 9.73% (Figure 1b). *PmNAC* genes on chromosomes 1 and 3 were clustered in the center of chromosomes. The uneven distribution of *PmNAC* on chromosomes reflected the diversification and complexity of *NAC* gene family.

### 3.2. Synteny Relationships and Duplication of PmNAC Genes

Duplication events are related to plant evolutionary patterns, and tandem duplication and segmental duplication are the sources of gene family expansion and genome complexity [67,68]. We identified 15 pairs of tandemly duplicated and 8 pairs of segmentally duplicated *PmNAC*s in the *P. mume* genome (Figure 1a). All chromosomes possessed tandemly duplicated genes except for chromosomes 4; segmentally duplicated genes were mainly found on chromosomes 1, 2, 4, 5, and 7 (Figure 1a). Chromosome 3 possessed the largest number of tandemly duplicated gene pairs, which implied that chromosome 3 might be more complex and diverse. Furthermore, the substitution rate of K_a_ (non-synonymous) and K_s_ (synonymous) were used to estimate the selection pressure and trace the divergence time of the duplicated events (Table 1). Among the 23 duplicated gene pairs, 9 pairs evolved under positive selection (K_a_/K_s_ > 1), and 14 pairs evolved under purifying selection (K_a_/K_s_ < 1). One duplicated gene pair (*PmNAC080* vs. *PmNAC080*) shared K_a_/K_s_ value above 2, suggesting that stronger positive selection occurred on this gene pair. Divergence time of the *PmNAC* genes suggested that the duplicated events traced to 87.11 million years ago (Mya) and continued up until 1.15 Mya, and most of duplicated gene pairs shared duplicated events dating back to 40–50 Mya.

**Figure 2:**
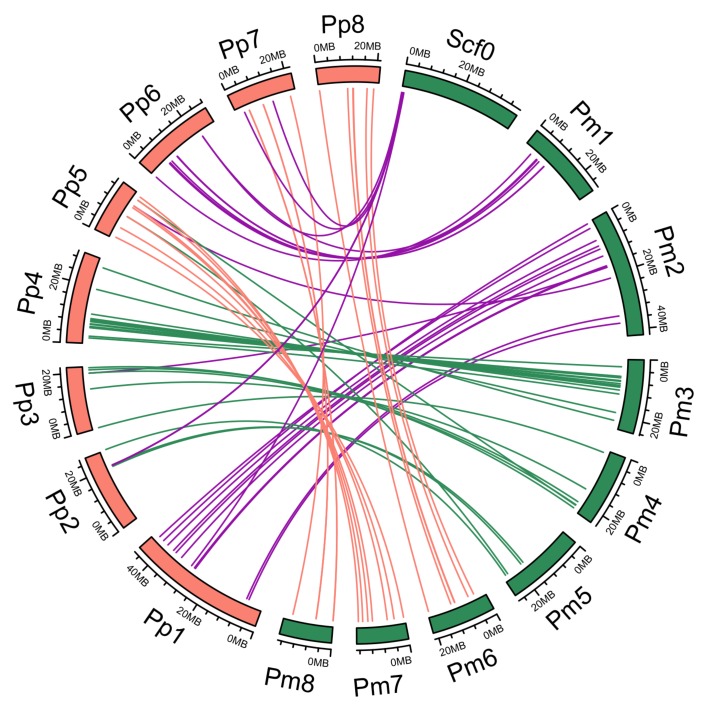
Syntenic analyses of *NAC* genes in *P. mume* (Pm) and *P. persica* (Pp). Green and orange blocks present *P. mume* and *P. persica* chromosomes, respectively. Lines denote syntenic *NAC* gene pairs on the chromosome. Purple lines present syntenic *PmNAC*s in scaffolds, Pm1 and Pm2; green lines present syntenic *PmNAC*s in Pm3, Pm4 and Pm5; orange lines present syntenic *PmNAC*s in Pm6, Pm7 and Pm8.

Syntenic analysis was performed using orthologous *NAC* gene pairs between *P. mume* and *P. persica* to explore their evolutionary events. 63 orthologous gene pairs were identified between *P. mume* and *P. persica* (Figure 2 and Table S3). This indicates that these two species have a close relationship. The divergence time of *NAC* genes between *P. mume* and *P. persica* was also calculated, suggesting that the duplication events began from 125.49 Mya and continued up until 0.69 Mya, and most of duplicated events occurred approximately 1–5 Mya. This showed that the time of speciation was shorter in these two species, providing further evidence of close relationship between *P. mume* and *P. persica*. In addition, the distribution of the syntenic genes across chromosomes and the subfamilies was also analyzed, showing that chromosomes 2 and 3 contained higher syntenic genes (Figure S1b). These syntenic genes were mainly clustered into subfamily II, III, IV, VI, and VII, which involved in growth and development, abiotic and biotic stress responses and formation of vascular vessels (Figure S1a).

### 3.3. Phylogenetic Analysis of PmNACs

**Figure 3:**
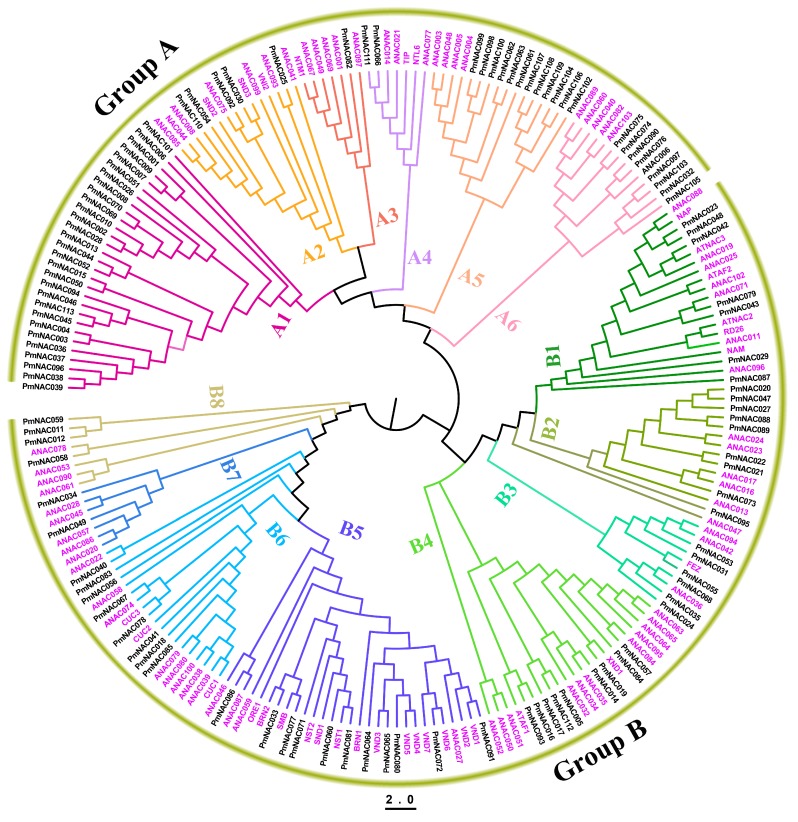
Phylogenetic analysis of NAC genes from *P. mume* and *Arabidopsis thaliana*. The maximum likelihood (ML) phylogenetic tree was generated by RAxML version 8 software using optimal JTT (Jones–Taylor–Thornton) models with gamma distributed rates. The Phylogenetic tree was divided into two groups (Groups A and B) and fourteen subgroups (Subgroups A1–6 and B1–8). Each subgroup is distinguished by a different color. Pink color presents *NAC* genes in *A. thaliana*.

To reveal the phylogenetic relationships and potential functional characteristics of *PmNAC* gene family, a phylogenetic tree was constructed using 218 full-length protein sequences from *P. mume* (113 sequences) and *A. thaliana* (105 sequences) (Table S4 and File S2). Finally, these NAC proteins were divided into two groups (A and B), including 14 well-supported subgroups (A1–6 and B1–8). In Group A, Subgroups A2, 3, 4, 5, and 6 corresponded to *Arabidopsis* VNI2, NTM1, NTL6, ANAC004, and ANAC082 gene classification, respectively (Figure 3), while Subgroup A1 did not include any NAC proteins of *Arabidopsis*, but only 29 PmNAC members. In Group B, Subgroups B1, 2, 3, 4, 5, 6, 7, and 8 corresponded to NAM, ANAC047, FEZ, ATAF1, NST, CUC, ANAC028, and ANAC078 gene classification, respectively (Figure 3). Noticeably, NACs with similar regulatory functions inclined to cluster together. For instance, membrane-associated TIP and NTL genes were clustered into Subgroup A4; secondary cell wall development related genes VNDs and NSTs were clustered into Subgroup B5; and CUCs were classified into Subgroup B6.

### 3.4. Conserved Motif, Gene Structure and Protein Interaction Analysis

To decipher the specific region of PmNAC proteins, ten distinct and non-redundant motifs were detected with high e-value by MEME tool. Ten distinct individual motifs were identified (File S1). Moreover, an independent phylogenetic tree of PmNACs was constructed using 113 full-length protein sequences, which classified PmNACs into fourteen subfamilies (Figure S2a). As expected, the PmNAC proteins with a close relationship shared similar motif composition (Figure S2b). It is obvious that the motif composition was clustered into two types: in the first type, motifs 1, 2, 3, 5, and 8 were present in subfamilies I–X, whereas, in the second type, motifs 4, 6, 7, 9 were found in subfamilies XI–XIV. Motif 4 was absent in subfamilies I, VII, and X. Motif 8 and 10 were absent in subfamily XIII. Most PmNACs processed 4–5 conserved motifs except for few extreme proteins. For instance, PmNAC098 had ten motifs and harbored two NAC domains, but PmNAC093 in subfamily II only had two motifs. Furthermore, motifs corresponding subdomains were also classified (File S1). Motifs 1 and 5 corresponded to Domains A and B; motifs 2 and 4 correspond to Subdomains C1 and C2; motifs 6 and 3 corresponded to Subdomains D1 and D2; and motif 7 corresponded to Domain E. As shown in Figure S2, most PmNACs in subfamilies I–X contained Domains A–D, and most PmNACs in subfamilies XI, XIII, XI and XIV contained Domains C, D, and E.

To assess the diversity of gene structure, we analyzed the exon/intron structures of *PmNAC* genes using the GSDS online tools. The number of exons/introns usually shared similarities in the same classes (Figure S3). For instance, subfamily I typically contained four exons; three exons were present in subfamily II; and only one exon was found in subfamilies XI–XIV except for *PmNAC052* and *PmNAC001* (Figure S3). However, the number of exons was greatly variable from subfamily III to X, ranging from one to fifteen; among them, most *PmNAC*s contained three to five exons except for some extreme ones. For instance, *PmNAC054* in subfamily III contained fifteen exons, whereas *PmNAC105* in subfamily IX contained only one exon.

**Figure 4:**
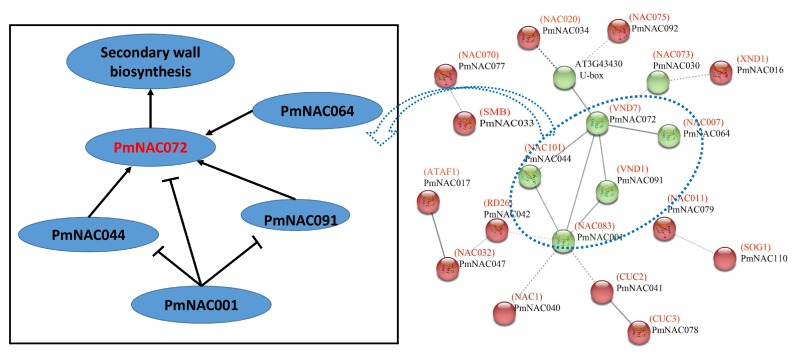
Interaction networks of the PmNACs based on their homology to *A. thaliana* NAC proteins. The thicker lines refer to stronger associations.

To further identify the putative function of PmNACs, the interactions of 113 PmNACs were predicted using STRNG 10.5 online tools based on homologous proteins of *Arabidopsis* (Figure 4). PmNAC072 showed high homology to VND7, which is a transcription activator involved in regulating protoxylem vessel differentiation [69]. VND7 is a key protein in this interaction network. VND7 is closely related to NAC007, NAC101, NAC083, VND1 and U-box, which are associated with the regulation of secondary wall thickening, xylem formation, stress tolerance and growth. PmNAC044, PmNAC064 and PmNAC091 are highly homologous to *Arabidopsis* NAC101, NAC007 and VND1, respectively, which can positively regulate VND7 [70]. However, PmNAC001 is highly homologous to NAC083, which is a transcriptional repressor that negatively regulates the expression of genes involved in xylem vessel formation [71]. In addition, PmNAC017 (ATAF1) vs. PmNAC047 (NAC032) and PmNAC042 (CUC2) vs. PmNAC078 also have strong interactions (Figure 4).

### 3.5. Membrane-Bound Analysis and miR164 Target Site Prediction

Three PmNAC proteins (PmNAC059/066/073) were identified, which contained α-helical trans-membrane (TM) domain (Figure S4). The TM positions of PmNAC059/066/073 were located in 562–585, 511–534 and 548–570 of the protein sequences, respectively (Figure S4a). PmNAC059/066/073 are highly homologous to *Arabidopsis* NAC2, TIP, and ANAC017, respectively. Furthermore, the hydrophobicity and tertiary structure of PmNAC protein were also analyzed. Obviously, the level of hydrophobicity was higher in TM position (Figure S4b). The illustration of the tertiary structure showed that all these TMs proteins had similar NAC domain, while α-helical structures of C-terminus were significantly different (Figure S4c).

It is well known that microRNA (a small non-coding RNA containing of about ~22 nucleotides) is involved in RNA silencing and post-transcriptional regulation mechanisms [72]. In *P. mume*, eight *Pmu-miRNA164*s have been identified [73]. We analyzed these *Pmu-miRNA164*s and their target genes using psRNATarget database online. We obtained five *Pmu-miRNA164*s and their corresponding target genes (Table S5), and the *Pmu-miRNA*164s sequences and secondary structures were quite different (Figure S5a). Three *PmNAC*s (*PmNAC040*/*041*/*083*) were targeted by *Pmu-miRNA164*s, and all of their target sites were located on exon1 (Figure S5b). *PmNAC040*/*041*/*083* are homologous to *NAC1*, *CUC2*, and *ATNAC5*, respectively, and all of them belonged to subfamily VI, suggesting that *miRNA164* targeted-genes were conserved.

### 3.6. Expression Pattern Analysis by Transcriptome

**Figure 5:**
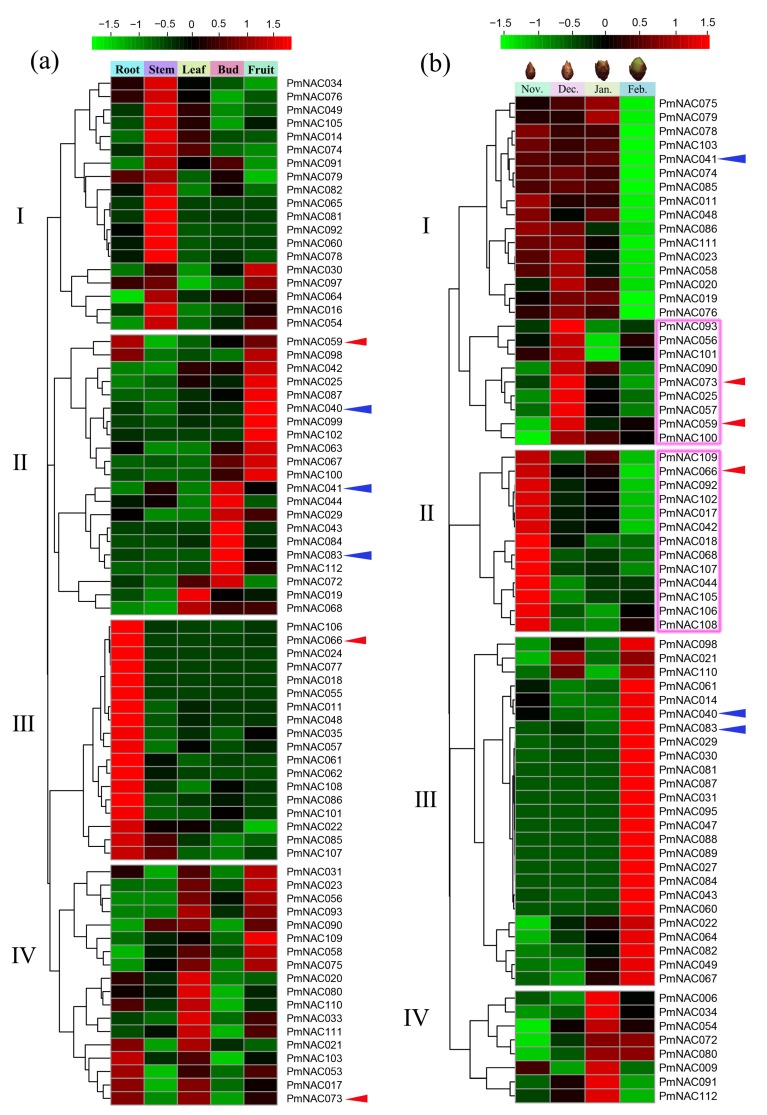
(**a**) Hierarchical clustering of expression profiles of *PmNAC*s in different tissues. (**b**) Expression profiles of *PmNAC*s in the flower bud during dormancy release. Pink rectangle presents genes showing specific high expression levels in flower bud in December and November. The expression levels were normalized by row using Z-Scores algorithms. Color scale at the top of heat map refers to relative expression level, and the color gradient from green to red presents an increasing expression level. Alpha numeric presents hierarchical clustering of gene expression. Red arrows show membrane-bound *PmNAC*s, and the blue arrows represent *Pmu-miRNA164* targeted genes.

To investigate the specific expression of *PmNAC*s, we analyzed the expression patterns of *PmNAC* genes in root, stem, leaf, bud and fruit; flower bud in November, December, January and February; and stems from Beijing, Chifeng and Gongzhuling during autumn (October), winter (January) and spring (March) based on available transcriptome data [62,63]. In all tissues, *PmNAC*s with the Fragments Per Kilobase Million (FPKM) >1 were collected for further analysis. We found that 76 *PmNAC* genes showed specific expression in root, stem, leaf, bud and fruit (Figure 5a); their FPKM value is shown in Table S6. *PmNAC*s with similar expression patterns were clustered into the same subset. As shown in Figure 5a, *PmNAC*s in subset I presented relatively higher expression levels in the stem, and most of these genes were mainly related to secondary wall formation. For instance, *PmNAC016/60*/*065* were orthologous genes of *Arabidopsis XND1*, *NST1*, and *VND4*, respectively (Table S6). *PmNAC*s in subset II presented specific expression in fruit and bud, including three *Pmu-miRNA164* targeted genes *PmNAC040*/*041*/*083*. *PmNAC*s in subset III showed higher expression levels in root, including a more highly expressed trans-membrane gene (*PmNAC066*). The expression of *PmNAC*s in subset IV was not very specific, and most of them were expressed in root, leaf and fruit.

A total of 71 *PmNAC* genes were expressed in flower bud, and most of them exhibited distinct temporal specificity during dormancy release (Figure 5b), their FPKM value is presented in Table S7. As shown in Figure 5b, 16 *PmNAC*s in subset I showed high expression levels from November to January, which were significantly down-regulated in February; among them, *PmNAC041* was *Pmu-miRNA164* targeted-gene. 22 *PmNAC*s (highlighted by pink rectangle in Figure 5b) were preferentially expressed in November and December, including three transmembrane genes *PmNAC059*/*66*/*73*. *PmNAC*s in subset III exhibited specifically higher expression levels in February, and *PmNAC*s in subset IV was up-regulated in January (Figure 5b).

**Figure 6:**
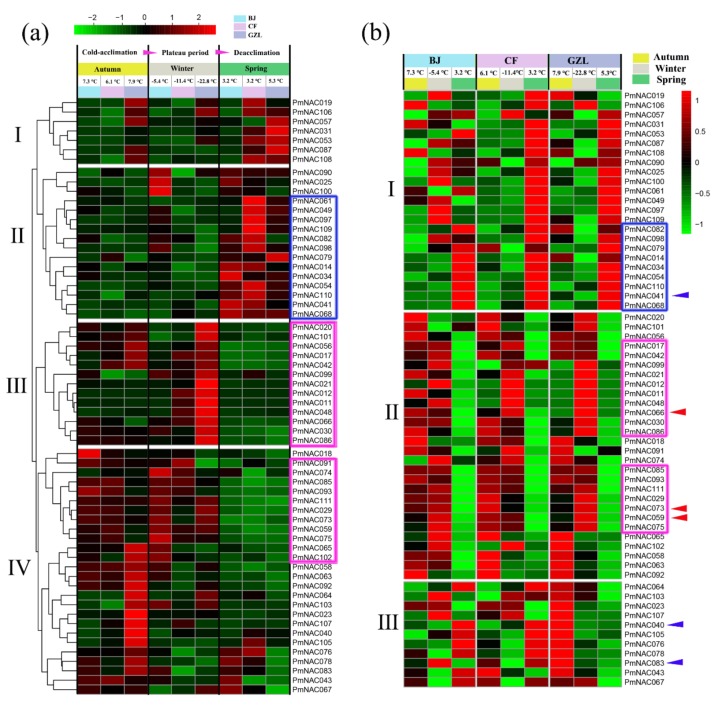
(**a**) Expression profiles of *PmNAC*s in stems of ‘Songchun’ in different regions (Beijing, Chifeng and Gongzhuling) and seasons (autumn, winter and spring). (**b**) Comparison of differential expression profiles of stems in Beijing, Chifeng and Gongzhuling during different seasons. Pink rectangle presents genes showing relatively high expression levels at low-temperature (below −5 °C). Aqua-blue rectangle indicates genes having consistent expression patterns and highly expressed in spring (above 3 °C). The expression levels were normalized by row using Z-Scores algorithms. Color scale at the top of heat map refers to relative expression level, and the color gradient from green to red shows an increasing expression level. Alpha numeric presents hierarchical clustering of gene expression. Red arrows present membrane-bound *PmNAC*s, and the blue arrows present *Pmu-miRNA164*-targeted-genes.

We further analyzed the expression profiles of *P. mume* (‘Songchun’) stems collected from three different growth regions—Beijing (BJ), Chifeng (CF) and Gongzhuling (GZL) in China—and their FPKM value is shown in Table S8. As shown in Figure 6a, 13 *PmNAC*s (subset II highlighted by blue rectangle) showed significantly high expression levels in spring; 24 *PmNAC*s (subsets III and IV highlighted by pink rectangle in Figure 6a) were prominently up-regulated by low temperature (below −5 °C), but downregulated in spring (temperature above 0 °C). To compare the expression patterns of these *PmNAC*s in stem in different periods, another heat map analysis was performed (Figure 6b). Even though *P. mume* ‘Songchun’ was planted in three different regions, these *PmNAC*s showed strikingly similar expression patterns in different periods (highlighted by rectangle in Figure 6b). Nine *PmNAC*s (highlighted by blue rectangle in Figure 6b), including *Pmu-miRNA164 PmNAC041*, showed almost identical expression patterns in three different periods (autumn, winter and spring) and all of them were highly expressed in spring. However, another subset *PmNAC*s highlighted by pink rectangle exhibited higher expression levels in autumn and winter, and all these genes also presented similar expression in different periods, including three transmembrane genes *PmNAC059*/*66*/*73* (Figure 6b).

### 3.7. Cold Response Patterns of PmNACs during Cold Treatment

**Figure 7:**
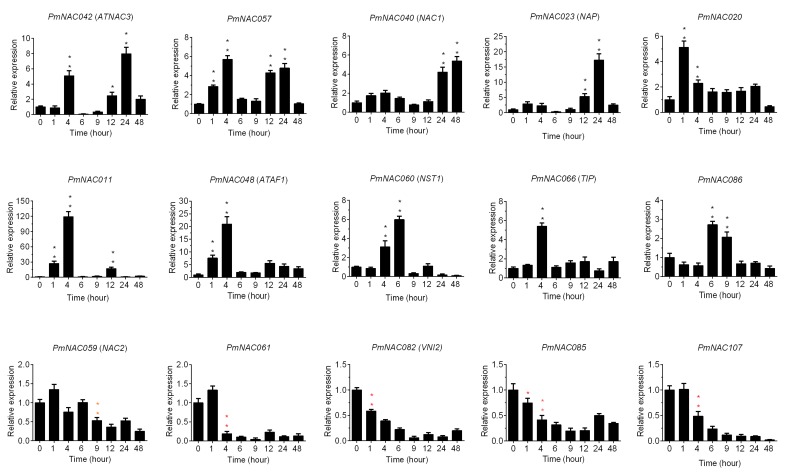
Expression analyses of 15 candidate *PmNAC*s in *P. mume* crossing lines (CP1) exposed to 4 °C for different time (0/1/4/6/912/24/48 h), where 0 h indicates control. The relative quantification method (2^−ΔΔCT^) was used to evaluate quantitative variation. Error bars represent standard error for three replicates. Significance levels of up-regulated expression: * < 0.05; ** < 0.01. Significance levels of down-regulated expression: * < 0.05; ** < 0.01.

To further investigate cold response patterns of *PmNAC*s, the seedlings of *P. mume* were treated at 4 °C for different time periods (0/1/4/6/912/24/48 h). In total, 15 potential *PmNAC*s were selected for qRT-PCR based on the analysis of transcription profiles and functional evolution. Details of these genes are shown in Table S9 and included *miR164*-targeted and membrane-bound genes. As shown in Figure 7, the majority of *PmNAC*s were up-regulated or down-regulated within 4 h during 4 °C treatment; *PmNAC011*/*20*/*42*/*48*/*57*/*60*/*66* were significantly up-regulated at 4 h in cold treatment; and *PmNAC040*, *PmNAC02* and *PmNAC086* were up-regulated at 24, 12 and 6 h in cold treatment, respectively. Moreover, all genes exhibited more than two-fold higher expression levels compared with control (0 h). However, *PmNAC059*/*61*/*82*/*85*/*107* were significantly down-regulated with the increase of treatment time under cold-stress (Figure 7).

## 4. Discussion

The NAC TFs have an important contribution to plant adaptation to complex land environment [1]. Plants have large number of NAC genes, and this gene family consists of more than 100 members in most plants [4,29,74], which can be involved in growth and development of plants or be induced by environmental stress. The genome of *P. mume* adds resolution to comprehend the phylogenetic relationship and duplication patterns of *NAC* gene family in *Prunus*. According to previous studies, genes showing a close evolutionary relationship usually perform similar function, thus we can understand and predict the potential function of genes on the basis of phylogenetic relationship of gene family [75,76]. In our study, 113 non-redundant *PmNAC* genes were identified, and the phylogenetic tree of *Arabidopsis* and *P. mume* with good bootstrap support was constructed. The genes were divided into 14 well-supported subgroups (Figure 3). The phylogenetic analysis revealed that *NAC* genes have high similarities within the same class of NACs in *Arabidopsis* and *P. mume*. For instance, NAC proteins such as SND1, NSTs and VNDs, clustered into Subgroup B5, were associated with the development of vascular vessel and the formation of secondary wall, suggesting that *PmNAC*s have similar function in subgroup B5. *P. mume* has more *NAC* genes than *Arabidopsis* [4], but fewer than rice [30] and poplar [28]. Duplicates can increase pernicious mutations in regulatory regions and retain complementary duplicates through purifying selection [77], which can contribute to the expansion of the gene family and functional diversification [78,79]. We identified 8 pairs of segmentally duplicated *PmNAC*s and 15 pairs of tandemly duplicated *PmNAC*s (Figure 1a and Table 1). Five tandemly duplicated gene pairs were found on chromosome 3, whereas chromosome 7 contained five gene pairs that were evolved by segmental duplication (Figure 1a). This suggests that tandemly duplicated genes have remarkably contributed to *NAC* gene family expansion. K_a_/K_s_ values of the duplicated gene pairs were calculated to estimate their evolution history. Sixteen duplicated gene pairs had K_a_/K_s_ values less than 1.0 (Table 1), indicating that most duplicated gene pairs evolved through purifying selection. In addition, we found that most duplicated gene pairs belonged to *FEZ*, *ATAF*, *NAP*, *CUC* and *NST* genes, which are related to plant growth and development or environmental stress responses (Figure S1).

Membrane-bound Transcription Factors (MTFs) harbor a distinctive transmembrane domain, which associates with nuclear, endoplasmic and plasma membrane [9,34]. Plant MTFs can mediate diverse kinds of stress responses thus playing a crucial role in stress resistance improvement [9]. During cold-acclimation, changes of biochemical compounds and membrane lipid composition can prevent phase changes resulting in irreversible injury of plant cells, which may play a vital roles in survival at extreme cold temperature (−20 to −30 °C) for woody plants [80]. In view of the great significance of MTFs, we identified three membrane-bound *NAC* genes (*PmNAC059*/*66*/*73*) with high confidence. *PmNAC059*, *PmNAC066* and *PmNAC073* were clustered into Subgroups B8, A4 and B2, respectively (Figure 3). *PmNAC059* was expressed in root, stem, leaf, dormant flower bud and stem at low temperature (Table S9). This suggests that *PmNAC059* may involve in diverse aspects of biological processes. *PmNAC066* genes was a homolog of *Arabidopsis NTL6*, which is often mediated by different stresses including cold [81], ABA [9] and drought [82]. NTL6 mediates ABA regulation of abiotic stress responses and is activated by ABA, overexpression of NTL6 was present a hypersensitive response to ABA in *Arabidopsis* [83]. *PmNAC073*, a homolog of *Arabidopsis ANAC017*, can mediate mitochondrial retrograde signaling, improve plant performance, and regulate the gene expression, plant growth and respiratory metabolism [82,84].

Previous studies indicated that the *miRNA164* can guide the cleavage of the mRNAs of *NAC* genes to regulate the growth, development, and responses to abiotic stress [85,86,87]. In our study, we identified three high confidence *miRNA164*-targeted genes (*PmNAC040*/*41*/*83*), belonging to Subgroup B6 (Figure S5 and Figure 3). These genes are highly homologous to *Arabidopsis CUC* genes. *PmNAC040* and *PmNAC041* is a tandemly duplicated gene pair (Figure 1), supporting the opinions that *CUC* lineages were generated by duplications [88]. In *Arabidopsis*, *miR164*-directed regulation of *CUC1*, plays an essential role in the development of embryonic, vegetative, floral and shoot apical meristems [89,5]. In *P. mume*, *PmNAC040*/*41*/*83* exhibited time- and organ-specific expression in bud, fruit, flower bud and stem under cold-stress (Figure 5 and Figure 6), suggesting that they are possibly involved in the development of bud and fruit, dormancy and cold stress response.

Protein interactions give us important clues in understanding gene function on a system-wide level [90]. According to the protein interaction networks, we found that PmNAC072 and PmNAC091 are similar to VNDs in *Arabidopsis*, which are involved in secondary wall biosynthesis [70]. PmNAC001, PmNAC044, PmNAC091 and PmNAC064 were associated with NAC083, NAC101, VND1 and NAC007, respectively, which were predicted to have a strong interaction with PmNAC072 (Figure 4). Among them, NAC101, NAC007 and VND1 can positively regulate VND7 [70], but NAC083 is a transcriptional repressor of VND7, VND1 and NAC101 and negatively regulates the formation of xylem vessel [71]. PmNAC017 was the homolog of ATAF1 that functioned in response to ABA, salt, drought, cold and oxidative stress [91,92]. PmNAC041 was more functionally related to CUC2, which was involved in plant growth and development [93], and was predicted to interact with CUC3. Based on these results, we speculate that these interaction proteins play crucial roles in regulating the xylem vessel differentiation, plant development and stress-response.

Cold is one of the most vital environmental factors that deeply affects plant growth, crop productivity and distribution area [94,95]. Cold acclimation is critical to help plants adapt to changes of temperature, and enhance cold tolerance [96]. The mechanisms of cold tolerance in plants are extremely complex, involving various signaling pathways and numerous genes. For instance, recent studies found that melatonin priming could improve cold-induced adverse effects in plants by increasing antioxidant enzyme activities and altering related gene expression [97,98]. Analysis of transcription profile revealed that *PmNAC*s were not only differentially expressed in different tissues, but also differentially expressed during the processes of dormancy release and cold acclimation. *PmNAC*s were expressed specifically in the stem and root, mainly belonging to subfamilies II/III/VII/VIII, which were associated with stress responses, development and the formation of xylem vessels and fibers (Figure 5a and Table S6), and had similar organ-specific expression patterns to *Arabidopsis* [4] and *Populous* [28]. *PmNAC025*/*40*/*42*/*63*/*67*/*87*/*99*/*102*/*100* were expressed in fruit, whereas *PmNAC029*/*43*/*41*/*44*/*72/83*/*84*/*112* were expressed in bud, suggesting that they might be related to the development of the respective organs. *PmNAC*s were also expressed differentially in the flower buds during dormancy release (Figure 5b). Notably, *PmNAC011*/*19*/*20*/*23*/*41*/*48*/*58*/*74*/*75*/*76*/*78*/*79*/*85*/*86*/*103*/*111* were highly expressed during dormancy (from November to January), and significantly down-regulated during dormancy release (February); among them, *PmNAC74*/*75*/*76* were tandemly duplicated genes. These results suggest that these *PmNAC*s are associated with dormancy release. Furthermore, a subset of *PmNAC*s highlighted by pink rectangle in Figure 5b, including three transmembrane genes (*PmNAC059*/*66*/*73*), was significantly up-regulated in November and December. It is clear that membrane-bound NACs play a key role in the adaptation to temperature changes through signal transduction, transcriptional activation and reduced membrane fluidity [81,95]. Thus, we speculated that these *PmNAC*s were involved in the cold response to protect flower bud at low temperature. In addition, *PmNAC*s were also highly expressed in the stem at low temperatures (below −5 °C). A subset of *PmNAC*s highlighted by pink rectangle in Figure 6a was remarkably up-regulated at temperature below −22 °C, and these *PmNAC*s had very similar expression patterns at three different regions (Beijing, Chifeng and Gongzhuling) in autumn, winter and spring (Figure 6b). Moreover, these putative candidate *PmNAC*s involved in the cold resistance also contained three membrane-bound *PmNAC*s (*PmNAC059*/*66*/*73*). Similarly, this co-expression pattern also appeared in flower bud mentioned above. We suggested that this subset of genes (highlighted by pink rectangle in Figure 6b) with stable co-expression with transmembrane *PmNAC*s might therefore be putative candidate responsible for cold resistance. Among these genes, *PmNAC017* and *PmNAC048* showed similarity to *ATAF1* in *Arabidopsis*. Overexpression of *ATAF1* can improve plant tolerance to ABA, salt and oxidative stresses [92]. In addition, ATAF1-dependent regulation of ABA-responsive genes was also observed in *Arabidopsis*, wild-type plants showed higher levels of ABA than the mutant since ATAF1-mediated repression of ABA biosynthesis [99]. This evidence provides that these genes might be involved in regulation of cold-stress.

As expected, 15 candidate *PmNAC*s were triggered by low-temperature (4 °C) and exhibited up or down regulation following treatment time (Figure 7). Seven genes (*PmNAC042*/*57*/*20*/*11*/*48*/*60*/*66*) were up-regulated by the cold treatments within 4 h, suggesting that these genes were strongly sensitive to low temperature and involved in cold-response. However, *PmNAC040*, a *miRNA164*-targeted gene in *P. mume*, was up-regulated under 24 h cold stress, speculating that cold sensitivity of this gene may be controlled by up-stream genes or other internal factors. The expression levels of five *PmNAC*s (*PmNAC059*/*61*/*82*/*85*/*107*) decreased with the increase of treatment times (Figure 7); these genes might be negatively regulated by cold stress responses and increase cold susceptibility. Hence, these genes might be potential candidate genes for breeding cold-tolerant *P. mume* cultivars through molecular and genetic techniques. Our results also contribute to further revealing the cold-stress response mechanism of *NAC* genes in other woody plants.

## 5. Conclusions

In conclusion, we first reported the genome-wide identification and characterization of the NACs in *P. mume*, including chromosomal location, duplicated genes, gene structure and conserved motifs, prediction of interaction proteins, membrane-bound and miRNA164-targeted genes and expression profiling. This study increases our understanding of function and evolution of *NAC* genes in plants. We identified 113 PmNACs and predicted 15 putative candidates involved in cold stress using bioinformatics and expression pattern analyses. Ten *PmNAC*s (*PmNAC42*/*57*/*40*/*23*/*20*/*11*/*48*/*60*/*66*/*86*), including membrane-bound and miRNA164-targeted genes, were up-regulated under cold stress compared to the control and might positively regulate cold stress responses. Five *PmNAC*s (*PmNAC59*/*61*/*82*/*85*/*107*) were down-regulated under cold stress. Multiple approaches were utilized to comprehensively predict potential cold-related genes, which could provide potential genes to improve plant cold tolerance through molecular and genetic techniques in our future research.

## Figures and Tables

**Table 1:** Estimated K_a_/K_s_ ratios and divergence times of the duplicated *PmNAC* genes.

Duplicated Gene Pairs			K_s_	K_a_	K_a_/K_s_	Type of Duplication	Type of Selection	Divergence Time (Mya)
*PmNAC003*	vs.	*PmNAC004*	0.253	0.072	0.283	Tandem	Purifying	8.43
*PmNAC011*	vs.	*PmNAC012*	0.240	0.189	0.787	Tandem	Purifying	8.01
*PmNAC021*	vs.	*PmNAC022*	0.574	0.656	1.144	Tandem	Purifying	19.12
*PmNAC036*	vs.	*PmNAC037*	0.076	0.033	0.438	Tandem	Purifying	2.54
*PmNAC037*	vs.	*PmNAC038*	1.102	1.670	1.515	Tandem	Purifying	36.73
*PmNAC038*	vs.	*PmNAC039*	1.050	1.659	1.580	Tandem	Positive	35.00
*PmNAC044*	vs.	*PmNAC045*	1.452	2.173	1.497	Tandem	Positive	48.39
*PmNAC045*	vs.	*PmNAC046*	0.093	0.025	0.270	Tandem	Purifying	3.09
*PmNAC058*	vs.	*PmNAC059*	1.743	2.088	1.198	Tandem	Positive	58.11
*PmNAC061*	vs.	*PmNAC062*	1.220	1.101	0.903	Tandem	Purifying	40.65
*PmNAC062*	vs.	*PmNAC063*	1.033	1.221	1.183	Tandem	Positive	34.42
*PmNAC074*	vs.	*PmNAC075*	0.034	0.036	1.035	Tandem	Positive	1.15
*PmNAC075*	vs.	*PmNAC076*	0.441	0.166	0.377	Tandem	Purifying	14.69
*PmNAC080*	vs.	*PmNAC081*	1.188	2.878	2.424	Tandem	Positive	39.58
*PmNAC088*	vs.	*PmNAC089*	1.454	1.926	1.324	Tandem	Positive	48.47
PmNAC005	vs.	*PmNAC112*	1.550	0.484	0.312	Segmental	Purifying	51.66
PmNAC011	vs.	*PmNAC093*	1.301	0.443	0.340	Segmental	Purifying	43.37
PmNAC024	vs.	*PmNAC055*	1.341	0.336	0.250	Segmental	Purifying	44.71
PmNAC025	vs.	*PmNAC082*	1.352	0.276	0.204	Segmental	Purifying	45.05
PmNAC056	vs.	*PmNAC083*	1.462	0.263	0.180	Segmental	Purifying	48.72
PmNAC057	vs.	*PmNAC082*	2.613	0.566	0.217	Segmental	Purifying	87.11
PmNAC060	vs.	*PmNAC081*	2.246	0.360	0.160	Segmental	Purifying	74.86
PmNAC064	vs.	*PmNAC080*	1.466	0.245	0.167	Segmental	Purifying	48.87

K_a_, non-synonymous; K_s_, synonymous; Mya, Million years ago.

## Supplementary Materials

The following are available online at http://www.mdpi.com/2073-4425/9/10/494/s1. Figure S1: Distribution of segmentally duplicated *NAC* gene pairs in *P. mume* and *P. persica*; Figure S2: Phylogenetic relationships and motif distribution of *P. mume*; Figure S3: Exon–intron structures of *PmNAC* genes; Figure S4: Prediction and analyses of membrane-bound *PmNAC*s; Figure S5: Analysis of mature *Pmu-miR164* sequence and its corresponding target sites; File S1: Schematic diagram of PmNAC protein motif; File S2: Protein sequences of *A. thaliana* and *P. mume*; Table S1: Primer sequences used for qRT-PCR; Table S2: Characteristics and classification of the 113 PmNAC proteins; Table S3: Duplication events and K_a_/K_s_ values of *NAC* genes in *P. mume* and *P. prersica*; Table S4: Typical NAC protein in *Arabidopsis*; Table S5: Identification of mature *miRNA164s* and targeted *NAC* genes in *P. mume*; Table S6: Expression profiles of 76 *PmNAC*s in different tissues (root, stem, leaf, bud and fruit); Table S7: Expression profiles of 71 *PmNAC*s in the flower bud during dormancy release; Table S8: Expression profiles of 62 *PmNAC*s in the stem in different regions and seasons; Table S9: Details of putative candidate genes for qRT-PCR.
