# Supplementary material for: Genome-Wide Analysis of the NAC Transcription Factor Gene Family Reveals Differential Expression Patterns and Cold-Stress Responses in the Woody Plant Prunus mume
Source: Genes (Basel). 2018 Oct 12;9(10):494. doi: 10.3390/genes9100494 (PMC6209978; doi:10.3390/genes9100494)
Supplement: Supplementary file 1 [file genes-09-00494-s001.zip › Supplementary materials/Supplementary Figure.pdf]

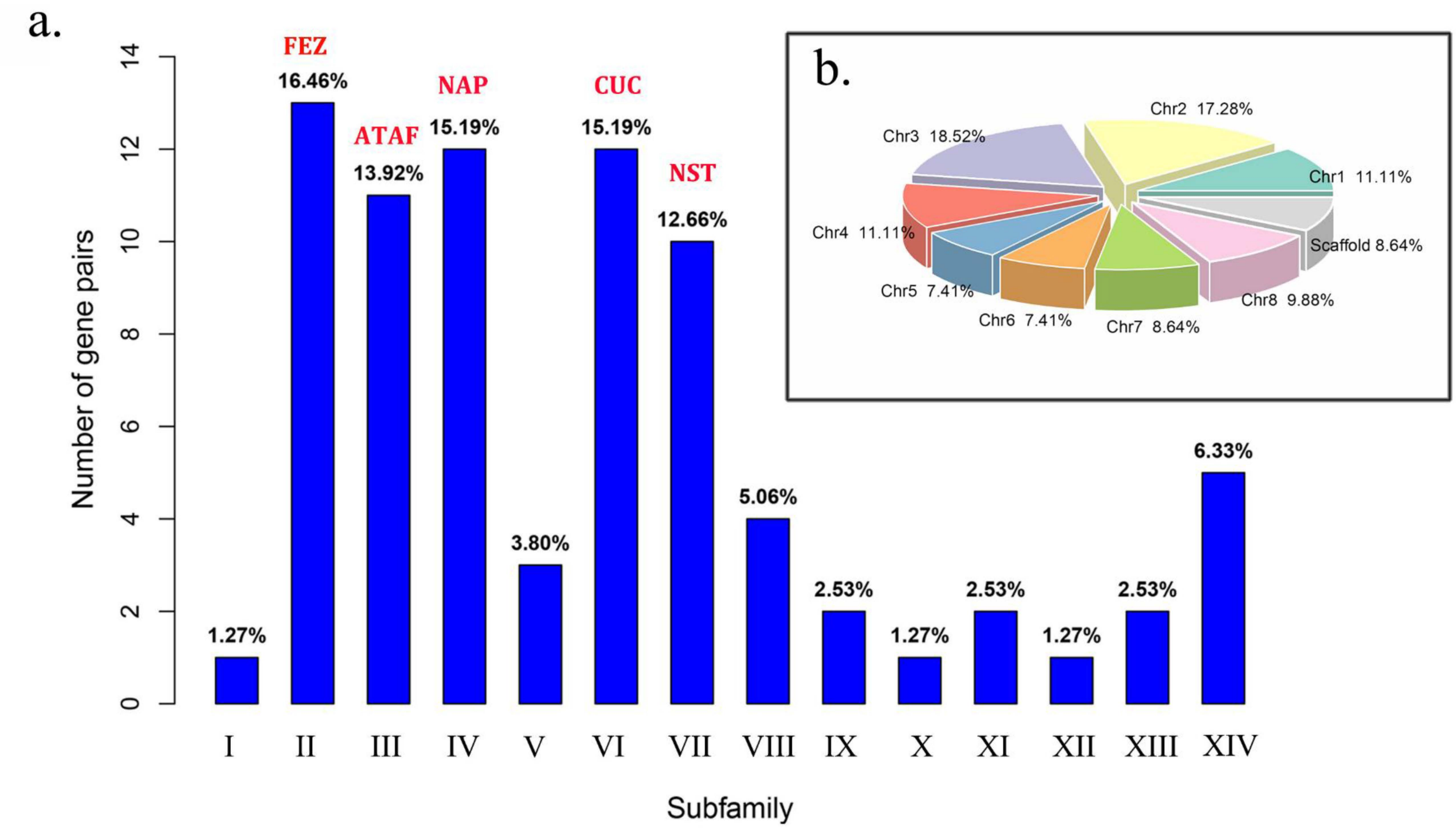

Supplementary Figure S1 Distribution of segmentally duplicated *NAC* gene pairs in *P. mume* and *P. persica*. (a) The number of segmentally duplicated gene pairs in each subfamily. (b) Percentage of segmentally duplicated genes on chromosomes. *FEZ, ATAF*, *NAP*, *CUC* and *NST* present orthologs of *Arabidopsis thaliana*.

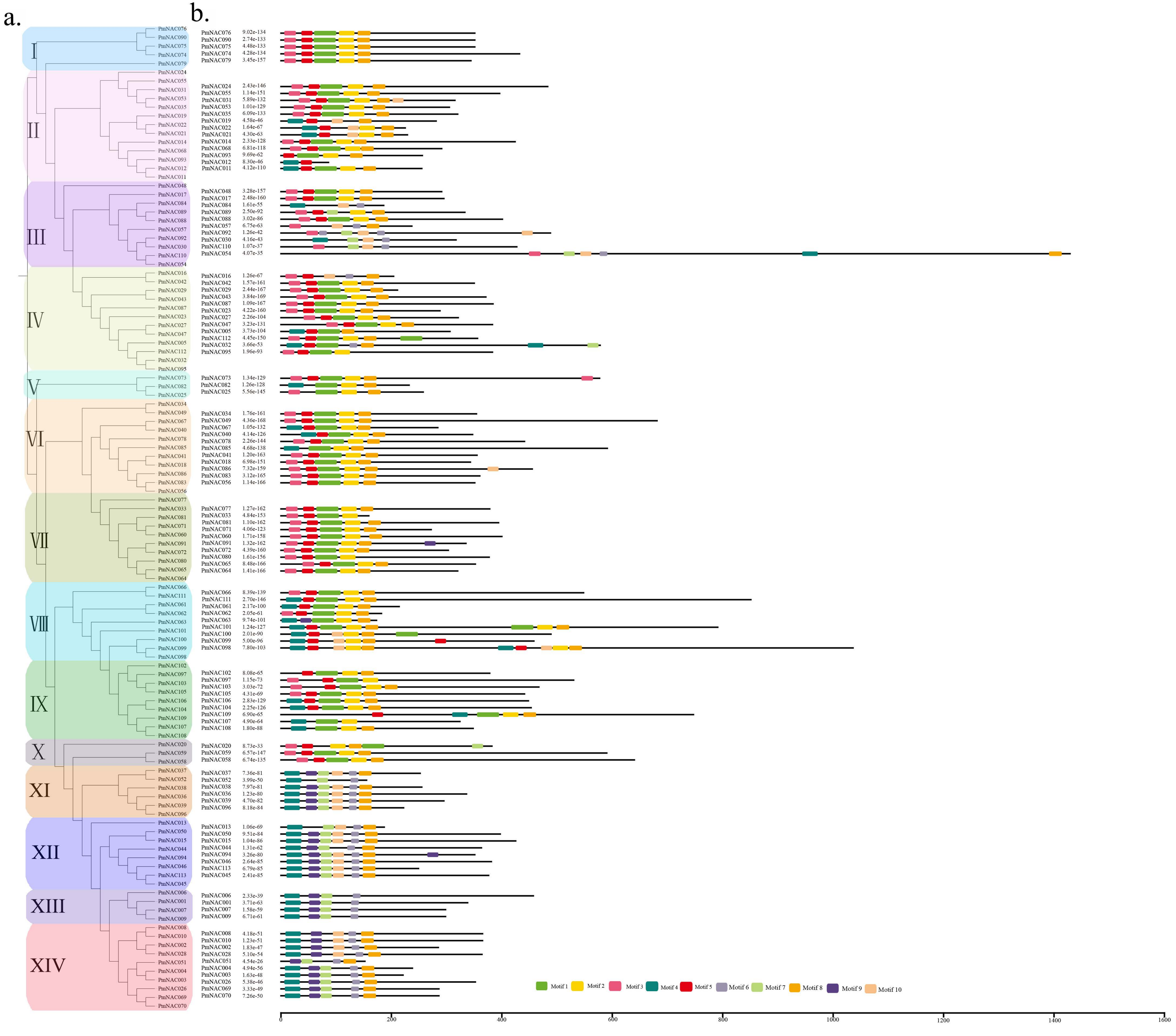

Supplementary Figure S2 Phylogenetic relationships and motif distribution of *P. mume*. (a) The maximum likelihood (ML) phylogenetic tree was generated by RAxML version 8 software using optimal LG models with gamma distributed rates. The 113 PmNAC proteins were clustered into fourteen phylogenetic subfamilies designated as I–XIV. Each subfamily is marked with different background colors. (b) Motif distribution of PmNAC proteins. Each motif is presented by a different colored block at the bottom. The details of the motif can be seen in Supplementary File S1.

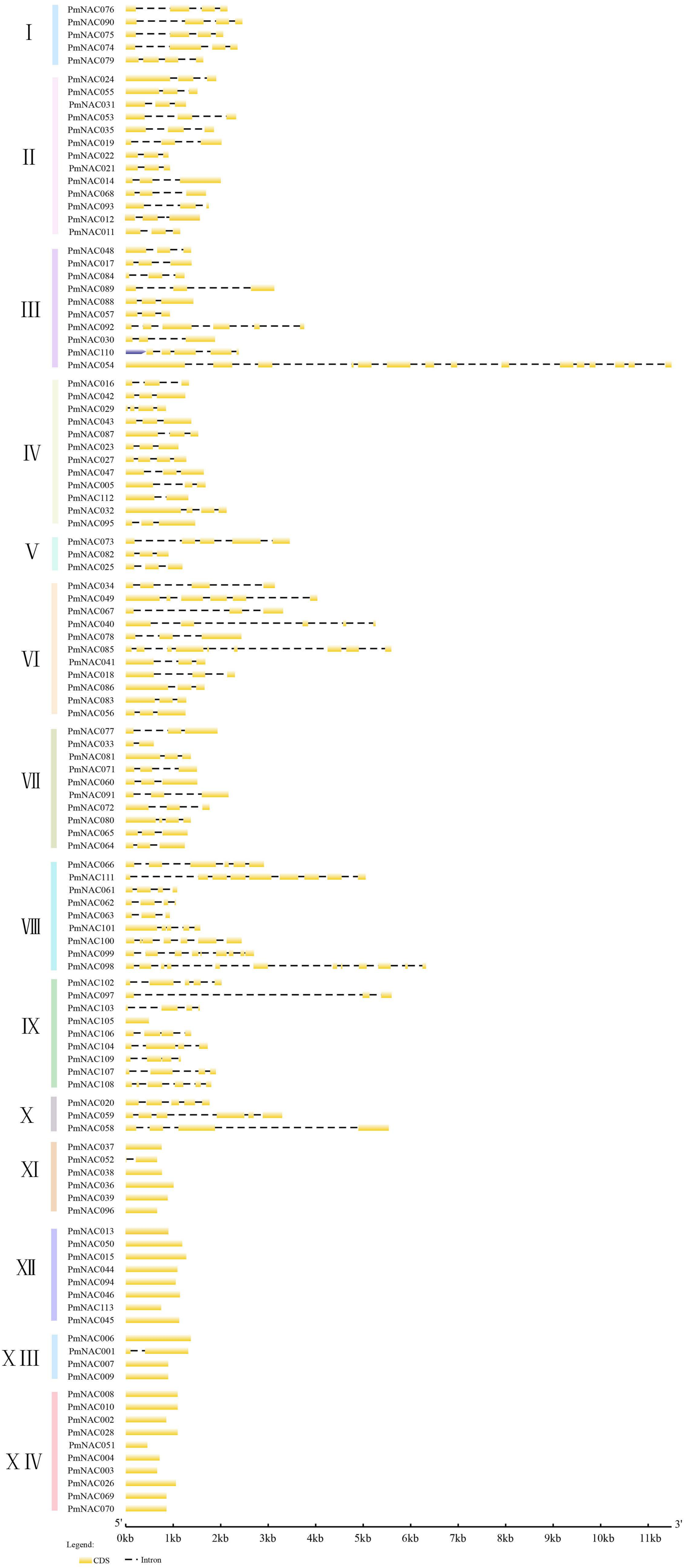

Supplementary Figure S3 Exon–intron structures of *PmNAC* genes. Exons and introns are indicated by yellow rectangles and dot-dashed lines, respectively. The scale estimates the lengths of exons and introns. The different bar colors present subfamilies I–XIV.

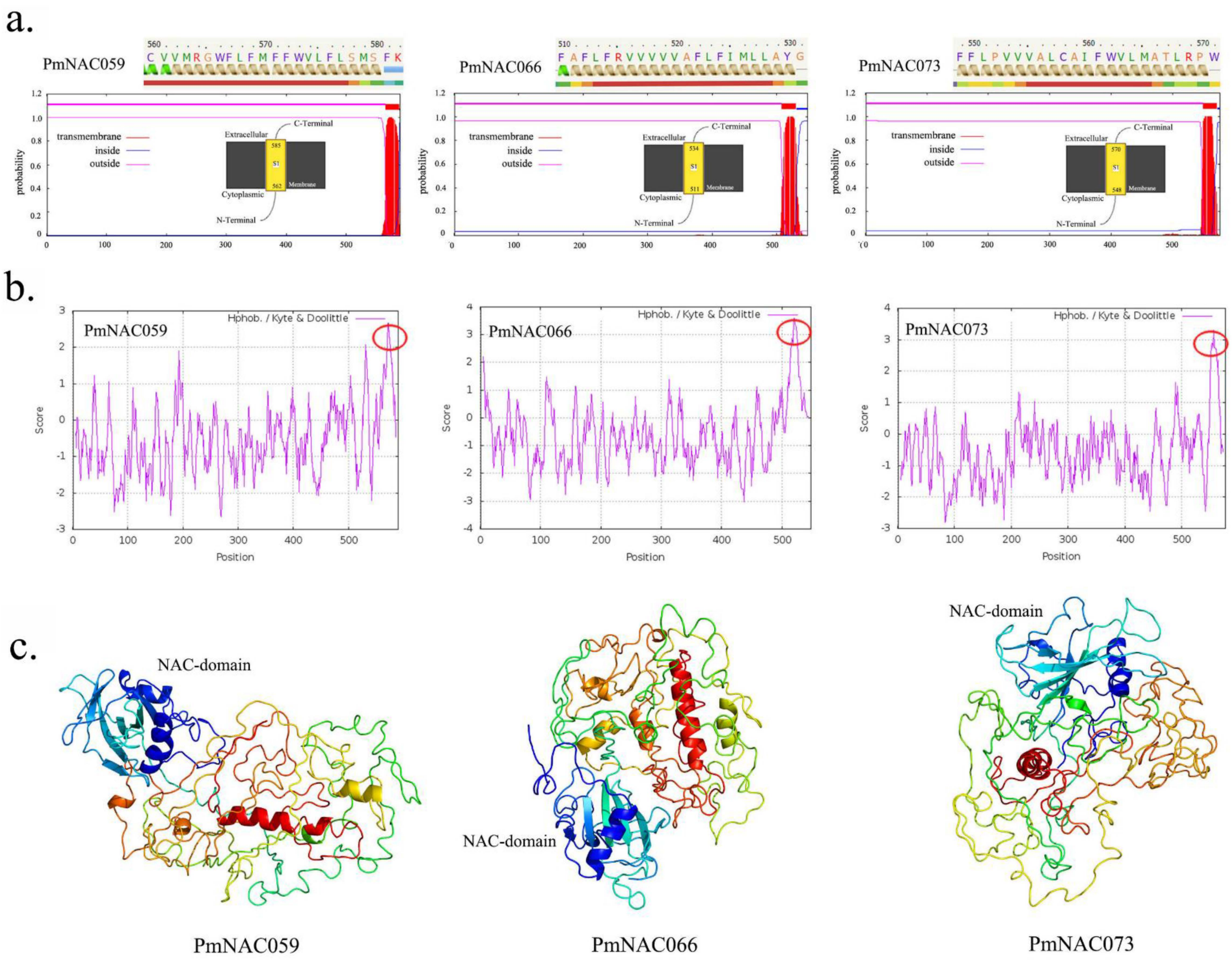

Supplementary Figure S4 Prediction and analyses of membrane-bound PmNACs. (a) Prediction of PmNAC proteins. Three transmembrane PmNAC proteins (PmNAC059/66/73) were predicted, including secondary structure and cartoons. (b) Analyses of hydrophobicity for three membrane PmNACs. The regions with red ellipse present strong hydrophobicity peak. (c) Tertiary structure of transmembrane proteins. The three membrane-bound proteins have similar NAC-domain and diverse α-helical structure.

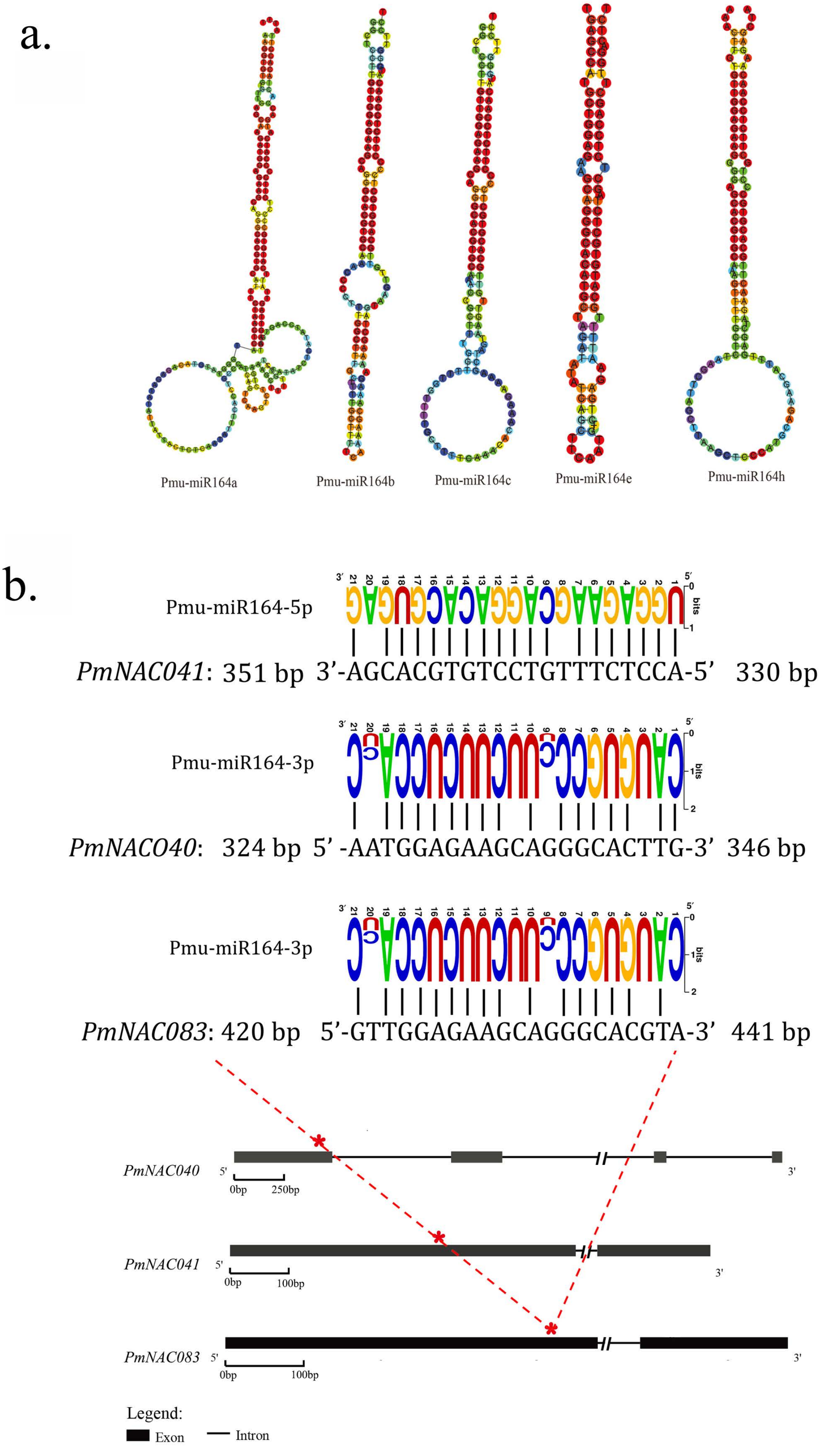

Supplementary Figure S5 Analysis of mature *Pmu-miR164* sequence and its corresponding target sites. (a) The secondary structure of the *Pmu-miR164* precursors sequences. (b) Mapping of *Pmu-miR164* target sites. The target sites of these three genes were located in exon1.
