# Supplementary material for: Genome-Wide Analysis of the NAC Transcription Factor Gene Family Reveals Differential Expression Patterns and Cold-Stress Responses in the Woody Plant Prunus mume
Source: Genes (Basel). 2018 Oct 12;9(10):494. doi: 10.3390/genes9100494 (PMC6209978; doi:10.3390/genes9100494)
Supplement: Supplementary file 1 [file genes-09-00494-s001.zip › Supplementary materials/Supplementary File S2.docx]

**Protein sequences of *Arabidopsis thaliana* and *P. mume***

>ANAC001

MEDQVGFGFRPNDEELVGHYLRNKIEGNTSRDVEVAISEVNICSYDPWNLRFQSKYKSRDAMWYFFSRRENNKGNRQSRTTVSGKWKLTGESVEVKDQWGFCSEGFRGKIGHKRVLVFLDGRYPDKTKSDWVIHEFHYDLLPEHQRTYVICRLEYKGDDADILSAYAIDPTPAFVPNMTSSAGSVVNQSRQRNSGSYNTYSEYDSANHGQQFNENSNIMQQQPLQGSFNPLLEYDFANHGGQWLSDYIDLQQQVPYLAPYENESEMIWKHVIEENFEFLVDERTSMQQHYSDHRPKKPVSGVLPDDSSDTETGSMIFEDTSSSTDSVGSSDEPGHTRIDDIPSLNIIEPLHNYKAQEQPKQQSKEKVISSQKSECEWKMAEDSIKIPPSTNTVKQSWIVLENAQWNYLKNMIIGVLLFISVISWIILVG

>ANAC002

MSELLQLPPGFRFHPTDEELVMHYLCRKCASQSIAVPIIAEIDLYKYDPWELPGLALYGEKEWYFFSPRDRKYPNGSRPNRSAGSGYWKATGADKPIGLPKPVGIKKALVFYAGKAPKGEKTNWIMHEYRLADVDRSVRKKKNSLRLDDWVLCRIYNKKGATERRGPPPPVVYGDEIMEEKPKVTEMVMPPPPQQTSEFAYFDTSDSVPKLHTTDSSCSEQVVSPEFTSEVQSEPKWKDWSAVSNDNNNTLDFGFNYIDATVDNAFGGGGSSNQMFPLQDMFMYMQKPY>A>NAC003

METPVGLRFCPTDEEIVVDYLWPKNSDRDTSHVDRFINTVPVCRLDPWELPCQSRIKLKDVAWCFFRPKENKYGRGDQQMRKTKSGFWKSTGRPKPIMRNRQQIGEKKILMFYTSKESKSDWVIHEYHGFSHNQMMMTYTLCKVMFNGGMREKSSSSPSSSGVSGIEQSRRDSLIPQLVNNSEGSSLHREDPSQFGDVLQEAPIEDAKLTEELVKWLMNDEDDAQIEDAIPIEEWETWLNDIDDAKEKSIMFMHDNRSDYRPPNSLTGVFSDDVSSDDNDSDLLTPKTNSIQTSSTCDSFGSSNHRIDQIKDLQESPTSTINLVSLTQEVSQALITSIDTAEKKKNPYDDAQGTEIGEHKLGQETIKKKRAGFFHRMIQKFVKKIHLCSSISRT

>ANAC004

MMNPVGFRFRPNDEEIVDHYLRPKNLDSDTSHVDEVISTVDICSFEPWDLPSKSMIKSRDGVWYFFSVKEMKYNRGDQQRRRTNSGFWKKTGKTMTVMRKRGNREKIGEKRVLVFKNRDGSKTDWVMHEYHATSLFPNQMMTYTVCKVEFKGEETEISSSSTGSEIEQIHSLIPLVNSSGGSEGSSFHSQELQNSSQSGVFANVQGESQIDDATTPIEEEWKTWLNNDGDEQRNIMFMQDHRSDYTPLKSLTGVFSDDSSDDNDSDLISPKTNSIGTSSTCASFASSNHQIDQTQHSPDSTVQLVSLTQEVSQGPGQVTVIREHKLGEESVKKKRASFVYRMIHRLVKKIHQCYSISRT

>NAC005

MANPVGFRFRPTDGEIVDIYLRPKNLESNTSHVDEVISTVDICSFDPWDLPSHSRMKTRDQVWYFFGRKENKYGKGDRQIRKTKSGFWKKTGVTMDIMRKTGDREKIGEKRVLVFKNHGGSKSDWAMHEYHATFSSPNQIMTYTLCKVKFKGERREFSVATGSGIKHTHSLIPPTNNSGVLSVETEGSLFHSQESQNPSQFSGFLDVDALDRDFCNILSDDFKGFFNDDDEQSKIVSMQDDRNNHTPQKPLTGVFSDHSTDGSDSDPISATTISIQTLSTCPSFGSSNPLYQITDLQESPNSIKLVSLAQEVSKTPGTGIDNDAQGTEIGEHKLGQETIKNKRAGFFHRMIQKFVKKIHLRT

>ANAC006

MKILPVGSRFCPTDLGLVRLYLRNKVERNQSSFITTMDIHQDYPWLLPHVNNPLFNNNEWYYFVPLTERGGKILSVHRKVAARGGSEGGTWRSNDGKKEIKDGHMQKGDGLRASDDLQKVVLCRIRYKKEANVNEFGLVNHQAHQTQDALTGFADQLEMMLEGQEDREQKEEADLTGFADSLETMLEGQEDHEQPEDADLTGFADSLETMLEGHEDREQPEEAELTVTQQQQQQQQQQQRQEDCDVTQEQEKDDVMVLINNPNDALALGNYEIIDLTRVDK

>VND4_NAC007

MNSFSHVPPGFRFHPTDEELVDYYLRKKVASKRIEIDFIKDIDLYKIEPWDLQELCKIGHEEQSDWYFFSHKDKKYPTGTRTNRATKAGFWKATGRDKAIYLRHSLIGMRKTLVFYKGRAPNGQKSDWIMHEYRLETDENGTPQEEGWVVCRVFKKRLAAVRRMGDYDSSPSHWYDDQLSFMASELETNGQRRILPNHHQQQQHEHQQHMPYGLNASAYALNNPNLQCKQELELHYNHLVQRNHLLDESHLSFLQLPQLESPKIQQDNSNCNSLPYGTSNIDNNSSHNANLQQSNIAHEEQLNQGNQNFSSLYMNSGNEQVMDQVTDWRVLDKFVASQLSNEEAATASASIQNNAKDTSNAEYQVDEEKDPKRASDMGEEYTASTSSSCQIDLWK

>ANAC008

MAGRSWLIDSNRIATKIMSASASSDPRQVVWKSNPSRHCPKCQHVIDNSDVVDDWPGLPRGVKFDPSDPEIIWHLLAKSGLSGLSSHPFIDEFIPTVNQDDGICYTHPKNLPGVKSDGTVSHFFHKAIKAYSTGTRKRRKIHDDDFGDVRWHKTGRTKPVVLDGVQRGCKKIMVLYGGKAVKTNWVMHQYHLGIEEDEKEGDYVVSKIFYQQPQQLVVKRGDKAEQEVSEDIFAAVTPTADPVTPKLATPEPRNAVRICSDSHIASDYVTPSDYVSAHEVSLAETSEVMCMEDEVQSIQPNHERPSSGPELEHGLENGAKEMLDDKEEQEKDRDNENQGEEDPTWFDSGSQFILNSQQLVEALSLCDDLLGSQDREENTNSGSLKDKQPCIADYAHLGPEDFKRDLEECQKIVLDPSNIELDTPPEFRLSQLEFGSQDSFLAWGTGKTD

>ANAC009

MAADPSEMGDRNNDGDQKMEDVLLPGFRFHPTDEELVSFYLKRKVQHNPLSIELIRQLDIYKYDPWDLPKFAMTGEKEWYFYCPRDRKYRNSSRPNRVTGAGFWKATGTDRPIYSSEGNKCIGLKKSLVFYKGRAAKGVKTDWMMHEFRLPSLSEPSPPSKRFFDSPVSPNDSWAICRIFKKTNTTTLRALSHSFVSSLPPETSTDTMSNQKQSNTYHFSSDKILKPSSHFQFHHENMNTPKTSNSTTPSVPTISPFSYLDFTSYDKPTNVFNPVSCLDQQYLTNLFLATQETQPQFPRLPSSNEIPSFLLNTSSDSTFLGEFTSHIDLSAVLAQEQCPPLVSLPQEYQETGFEGNGIMKNMRGSNEDHLGDHCDTLRFDDFTSTINENHRHHQDLKQNMTLLESYYSSLSSINSDLPACFSSTT

>ANAC010

MSWCDGSDDNYDLNLERVSNTDHPSVQLKDQSQSCVTSRPDSKISAETPITTCPSCGHKLHHHQDDQVGSIKDLPSLPAGVKFDPSDKEILMHLEAKVSSDKRKLHPLIDEFIPTLEGENGICYTHPEKLPGVSKDGQVRHFFHRPSKAYTTGTRKRRKVSTDEEGHETRWHKTGKTRPVLSQSGETGFKKILVLYTNYGRQKKPEKTNWVMHQYHLGSSEDEKDGEPVLSKVFYQTQPRQCGSMEPKPKNLVNLNRFSYENIQAGFGYEHGGKSEETTQVIRELVVREGDGSCSFLSFTCDASKGKESFMKNQ

>ANAC011

MVGSFLPPGFRFYPTDEELVGYYLHRRNEGLEIELEIIPLMDLYKFDPWELPEKSFLPNRDMEWFFFCHRDRKYQNGSRINRATKSGYWKATGKDRKIVCHSSSSSSSSSITGCRKTLVFYMGRAPFGGRTEWVMHEYRLFDNDTSQGSLNFKGDFALCRVIKRNEHTLKKCEIISPEVSDESLSNNVNNFCQASDLEKGSCDASNTRLSSPDFILESSFQGNSHSKTEEDSGFQVFTLPEFEYPLEVFADLNFDLEMEDPFMFDYHPEPHMNNEVMSHHIRG

>ANAC012_SND1

MADNKVNLSINGQSKVPPGFRFHPTEEELLHYYLRKKVNSQKIDLDVIREVDLNKLEPWDIQEECRIGSTPQNDWYFFSHKDKKYPTGTRTNRATVAGFWKATGRDKIICSCVRRIGLRKTLVFYKGRAPHGQKSDWIMHEYRLDDTPMSNGYADVVTEDPMSYNEEGWVVCRVFRKKNYQKIDDCPKITLSSLPDDTEEEKGPTFHNTQNVTGLDHVLLYMDRTGSNICMPESQTTTQHQDDVLFMQLPSLETPKSESPVDQSFLTPSKLDFSPVQEKITERPVCSNWASLDRLVAWQLNNGHHNPCHRKSFDEEEENGDTMMQRWDLHWNNDDNVDLWSSFTESSSSLDPLLHLSV

>ANAC13

MDLSVENGGLAPGFRFHPTDEELVVYYLKRKIRRKKLRVEAIGETDVYKFDPEELPEKALYKTRDRQWFFFSLRDRKHGSRSSRATERGYWKATGKDRVIHCDSRPVGEKKTLVFHRGRAPNGERTNWVMHEYTLHKEELKRCGGEDVKDAYVLYKIYKKSGSGPKNGEQYGAPFIEEEWAEDDDDDVDEPANQLVVSASVDNSLWGKGLNQSELDDNDIEELMSQVRDQSGPTLQQNGVSGLNSHVDTYNLENLEEDMYLEINDLMEPEPEPTSVEVMENNWNEDGSGLLNDDDFVGADSYFLDLGVTNPQLDFVSGDLKNGFAQSLQVNTSLMTYQANNNQFQQQSGKNQASNWPLRNSYTRQINNGSSWVQELNNDGLTVTRFGEAPGTGDSSEFLNPVPSGISTTNEDDPSKDESSKFASSVWTFLESIPAKPAYASENPFVKLNLVRMSTSGGRFRFTSKSTGNNVVVMDSDSAVKRNKSGGNNDKKKKKNKGFFCLSIIGALCALFWVIIGTMGGSGRPLLW

>ANAC014

MNQIKNKTLPEMTTEQALLSMEALPLGFRFRPTDEELINHYLRLKINGRDLEVRVIPEIDVCKWEPWDLPGLSVIKTDDQEWFFFCPRDRKYPSGHRSNRATDIGYWKATGKDRTIKSKKMIIGMKKTLVFYRGRAPRGERTNWIMHEYRATDKELDGTGPGQNPYVLCRLFHKPSDSCDPAHCEEIEKVNFTPTTTTRCSPDDTSSEMVQETATSGVHALDRSDDTERCLSDKGNNDVKPDVSVINNTSVNHAETSRAKDRNLGKTLVEENPLLRDVPTLHGPILSEKSYYPGQSSIGFATSHMDSMYSSDFGNCDYGLHFQDGASEQDASLTDVLDEVFHNHNESSNDRKDFVLPNMMHWPGNTRLLSTEYPFLKDSVAFVDGSAEVSGSQQFVPDILASRWVSEQNVDSKEAVEILSSTGSSRTLTPLHNNVFGQYASSSYAAIDPFNYNVNQPEQSSFEQSHVDRNISPSNIFEFKARSRENQRDLDSVVDQGTAPRRIRLQIEQPLTPVTNKKERDADNYEEEDEVQSAMSKVVEEEPANLSAQGTAQRRIRLQTRLRKPLITLNNTKRNSNGREGEASHRKCEMQEKEDISSSSSWQKQKKSLVQFSSVVIIVAVIVVLVEIWKESRDAKCSFLFHQLDSFKGMFT

>ANAC015

MSSSNGGVPPGFRFHPTDEELLHYYLKKKISYEKFEMEVIKEVDLNKIEPWDLQDRCKIGSTPQNEWYFFSHKDRKYPTGSRTNRATHSGFWKATGRDKCIRNSYKKIGMRKTLVFYKGRAPHGQKTDWIMHEYRIEDTEDDPCEDGWVVCRVFKKKNLFKVGNDVGSNISNNRLEARSFIRRESPYQGISMFELNKPEEISVHQYPQPPMFQPHHKPLSIGYDYSLALLPRESEYQQACQPSGVEVGTCKAVSEWGIVNCNMVSHEDSSRAMRFEDDGNNTSSTVQPPSNLLSLRGENGFLGLF

>ANAC016

MVDSSRDSCFKAGKFSAPGFRFHPTDEELVVYYLKRKICCKKLRVNAIGVVDVYKVDPSELPGNFQHLLIDFDSCLSMLKTGDRQWFFFTPRNRKYPNAARSSRGTATGYWKATGKDRVIEYNSRSVGLKKTLVFYRGRAPNGERTDWVMHEYTMDEEELGRCKNAKEYYALYKLYKKSGAGPKNGEQYGAPFQEEEWVDSDSEDADSVAVPDYPVVRYENGPCVDDTKFCNPVKLQLEDIEKLLNEIPDAPGVNQRQFDEFVGVPQGNSAEVIQSTLLNNSSGEYIDPRTNGMFLPNGQLYNRDSSFQSHLNSFEATSGMAPLLDNEKEEYIEMNDLLIPELGASSTEKSTEFLNHGEFGDVNEYDQLFNDISVFQGTSTDLSCLSNFTNNTSGQRQQLLYEQFQYQTPENQLNNYMHPSTTLNQFTDNMWFKDDQAALYVQPPQSSSGAFTSQSTGVMPESMNPTMSVNPQYKEGQNGGGTRSQFSSALWELLESIPSTPASACEGPLNQTFVRMSSFSRIRFNGTSVTSRKVTVAKKRISNRGFLLLSIMGALCAIFWVFKATVGVMGRPLLS

>NAC017

MADSSPDSCFKGGKFSAPGFRFHPTDEELVMYYLKRKICRKRLRVNVIGVVDVYKMDPEELPGQSMLKTGDRQWFYFTPRSRKYPNAARSNRGTENGYWKATGKDRVIEYNSRSVGLKKTLVFYRGRAPSGERTDWVMHEYTMDEDELGRCKNPQEYYALYKLFKKSGAGPKNGEQYGAPFQEEEWVDDDNEDVNAIAVAVPEQPVVRYEDARRVDERRLFNPVILQLEDIDELLNGIPNAPGVPQRCIPQVNSEEELQSTLVNNSAREFLPNGQQYNRPSSFDSLETAEVTSAPLVFEKEDFIEMDDLLLIPEFGASSTEKAAQFSNHGEFDDFNEFDQLFHDVSMSLDMEPIDQGTSANLSSLSDSANYTSDQKQQLLYQQFQDQTPENQLNNIMDPSTTLNQITSDIWFEDDQAILFDQQQSFSGAFASPSSGVMPDSTNPTMSVNAQGHEIQNGGGTTSQFSSALWALMDSIPSTPASACEGPLNRTFVRMSSFSRMRFNGKANGTPVSTTIAKKGIRNRGFLLLSIVGALCAIFWVLVATVRVSGRSLLLKD

> ANAC018

MESTDSSGGPPPPQPNLPPGFRFHPTDEELVIHYLKRKADSVPLPVAIIADVDLYKFDPWELPAKASFGEQEWYFFSPRDRKYPNGARPNRAATSGYWKATGTDKPVISTGGGGSKKVGVKKALVFYSGKPPKGVKSDWIMHEYRLTDNKPTHICDFGNKKNSLRLDDWVLCRIYKKNNSTASRHHHHLHHIHLDNDHHRHDMMIDDDRFRHVPPGLHFPAIFSDNNDPTAIYDGGGGGYGGGSYSMNHCFASGSKQEQLFPPVMMMTSLNQDSGIGSSSSPSKRFNGGGVGDCSTSMAATPLMQNQGGIYQLPGLNWYS

>ANAC019

MGIQETDPLTQLSLPPGFRFYPTDEELMVQYLCRKAAGYDFSLQLIAEIDLYKFDPWVLPNKALFGEKEWYFFSPRDRKYPNGSRPNRVAGSGYWKATGTDKIISTEGQRVGIKKALVFYIGKAPKGTKTNWIMHEYRLIEPSRRNGSTKLDDWVLCRIYKKQSSAQKQVYDNGIANAREFSNNGTSSTTSSSSHFEDVLDSFHQEIDNRNFQFSNPNRISSLRPDLTEQKTGFHGLADTSNFDWASFAGNVEHNNSVPELGMSHVVPNLEYNCGYLKTEEEVESSHGFNNSGELAQKGYGVDSFGYSGQVGGFGFM

>ANAC020

MAPMSLPPGFRFHPTDEELVAYYLDRKVNGQAIELEIIPEVDLYKCEPWDLPEKSFLPGNDMEWYFYSTRDKKYPNGSRTNRATRAGYWKATGKDRTVESKKMKMGMKKTLVYYRGRAPHGLRTNWVMHEYRLTHAPSSSLKESYALCRVFKKNIQIPKRKGEEEEAEEESTSVGKEEEEEKEKKWRKCDGNYIEDESLKRASAETSSSELTQGVLLDEANSSSIFALHFSSSLLDDHDHLFSNYSHQLPYHPPLQLQDFPQLSMNEAEIMSIQQDFQCRDSMNGTLDEIFSSSATFPASL

> ANAC021

MACVGGKDWYFYSQRDRKYATGLRTNRATATGYWKATGKDRTILRKGKLVGMRKTLVFYQGRAPRGRKTDWVMHEFRLQGSHHPPNHSLSSPKEDWVLCRVFHKNTEGVICRDNMGSCFDETASASLPPLMDPYINFDQEPSSYLSDDHHYIINEHVPCFSNLSQNQTLNSNLTNSVSELKIPCKNPNPLFTGGSASATLTGLDSFCSSDQMVLRALLSQLTKIDGSLGPKESQSYGEGSSESLLTDIGIPSTVWNC

> ANAC022

METEEEMKESSISMVEAKLPPGFRFHPKDDELVCDYLMRRSLHNNHRPPLVLIQVDLNKCEPWDIPKMACVGGKDWYFYSQRDRKYATGLRTNRATATGYWKATGKDRTILRKGKLVGMRKTLVFYQGRAPRGRKTDWVMHEFRLQGSHHPPNHSLSSPKEDWVLCRVFHKNTEGVICRDNMGSCFDETASASLPPLMDPYINFDQEPSSYLSDDHHYIINEHVPCFSNLSQNQTLNSNLTNSVSELKIPCKNPNPLFTGGSASATLTGLDSFCSSDQMVLRALLSQLTKIDGSLGPKESQSYGEGSSESLLTDIGIPSTVWNC

>ANAC023

MKVEDEATYELIKDELMKAEDEATYWLIKEELIKAEDDVIISRYLKRMIVNGDSWPDHFIEDVDVFNKNPNEEFHSQSPRFVIVKPRTENCGRTDGCQSGCWRIIGRDKLIKSKETGKILGFKKILKFCLKKKPREYKRSWVMEEYRLNNNLNCKQDHVICKIRFMFDAEISFLLAKHFSCLSTRSPLPANQLLPAYGVCFFDSEAEGAFYLETIIGYDGNTWPSYVTNDVYRLHPLTLVDPQDDKFKEFGTCIFANRTKTCGKTDECDGGGCWRIVEGHRVIKSKGKVLGYRRIFQFSENEEPRNVCEGEDPKKTAWFIEEYRPDENNKKDKVLCVIKFLIPLNQR

>ANAC024

MEEDAAFDLLKAELLNAEDDAIISRYLKRMVVNGDSWPDHFIEDADVFNKNPNVEFDAESPSFVIVKPRTEACGKTDGCETGCWRIMGRDKPIKSTETVKIQGFKKILKFCLKRKPRGYKRSWVMEEYRLTNNLNWKQDHVICKIRFMFEAEISFLLAKHFYTTSESLPRNELLPAYGFLSSDKQLEDVSYPVTIMTSEGNDWPSYVTNNVYCLHPLELVDLQDRMFNDYGTCIFANKTCGKTDRCINGGYWKILHRDRLIKSKSGIVIGFKKVFKFHETEKERYFCGGEDVKVTWTLEEYRLSVKQNKFLCVIKFTYDN

>ANAC025

MENMGDSSIGPGHPHLPPGFRFHPTDEELVVHYLKKKADSVPLPVSIIAEIDLYKFDPWELPSKASFGEHEWYFFSPRDRKYPNGVRPNRAATSGYWKATGTDKPIFTCNSHKVGVKKALVFYGGKPPKGIKTDWIMHEYRLTDGNLSTAAKPPDLTTTRKNSLRLDDWVLCRIYKKNSSQRPTMERVLLREDLMEGMLSKSSANSSSTSVLDNNDNNNNNNEEHFFDGMVVSSDKRSLCGQYRMGHEASGSSSFGSFLSSKRFHHTGDLNNDNYNVSFVSMLSEIPQSSGFHANGVMDTTSSLADHGVLRQAFQLPNMNWHS

> ANAC026

MNSFSQVPPGFRFHPTDEELVDYYLRKKVASKRIEIDIIKDVDLYKIEPCDLQELCKIGNEEQSEWYFFSHKDKKYPTGTRTNRATKAGFWKATGRDKAIYIRHSLIGMRKTLVFYKGRAPNGQKSDWIMHEYRLETSENGTPQEEGWVVCRVFKKKLAATVRKMGDYHSSPSQHWYDDQLSFMASEIISSSPRQFLPNHHYNRHHHQQTLPCGLNAFNNNNPNLQCKQELELHYNQMVQHQQQNHHLRESMFLQLPQLESPTSNCNSDNNNNTRNISNLQKSSNISHEEQLQQGNQSFSSLYYDQGVEQMTTDWRVLDKFVASQLSNDEEAAAVVSSSSHQNNVKIDTRNTGYHVIDEGINLPENDSERVVEMGEEYSNAHAASTSSSCQIDL

>ANAC027

MQAEEIICRVSDEEIIENYLRPKINGETSSIPRYVVELAEELYTVEPWLLPRQTAPILNPGEWFYFGKRNRKYSNLEGVHCEGSWILEDGCIAVLSKETGEEIGGTTRFRYYYRNKGDKESRLKMSNWFMREYRLYYKSRRVFNGRQVFCIITCNDEHFIE

>ANAC028_NAC028

MAPVSMPPGFRFHPTDEELVIYYLKRKINGRTIELEIIPEIDLYKCEPWDLPGKSLLPSKDLEWFFFSPRDRKYPNGSRTNRATKAGYWKATGKDRKVTSHSRMVGTKKTLVYYRGRAPHGSRTDWVMHEYRLEEQECDSKSGIQDAYALCRVFKKSALANKIEEQHHGTKKNKGTTNSEQSTSSTCLYSDGMYENLENSGYPVSPETGGLTQLGNNSSSDMETIENKWSQFMSHDTSFNFPPQSQYGTISYPPSKVDIALECARLQNRMLPPVPPLYVEGLTHNEYFGNNVANDTDEMLSKIIALAQASHEPRNSLDSWDGGSASGNFHGDFNYSGEKVSCLEANVEAVDMQEHHVNFKEERLVENLRWVGVSSKELEKSFVEEHSTVIPIEDIWRYHNDNQEQEHHDQDGMDVNNNNGDVDDAFTLEFSENEHNENLLDKNDHETTSSSCFEVVKKVEVSHGLFVTTRQVTNTFFQQIVPSQTVIVYINPTDGNECCHSMTSKEEVHVRKKINPRINGVSSTVLGQWRKFAHVIGFIPMLLLMRCVHRGNSNKNRGSEGYSRQPTRGDCNNRGTILMMENAVVRRKIWKKKKEKNMVDEQGFRFQDSFVLKKLGLSLAIILAVSTISLI

> ANAC029

MEVTSQSTLPPGFRFHPTDEELIVYYLRNQTMSKPCPVSIIPEVDIYKFDPWQLPEKTEFGENEWYFFSPRERKYPNGVRPNRAAVSGYWKATGTDKAIHSGSSNVGVKKALVFYKGRPPKGIKTDWIMHEYRLHDSRKASTKRNGSMRLDEWVLCRIYKKRGASKLLNEQEGFMDEVLMEDETKVVVNEAERRTEEEIMMMTSMKLPRTCSLAHLLEMDYMGPVSHIDNFSQFDHLHQPDSESSWFGDLQFNQDEILNHHRQAMFKF

>ANAC030

MDNIMQSSMPPGFRFHPTEEELVGYYLDRKINSMKSALDVIVEIDLYKMEPWDIQARCKLGYEEQNEWYFFSHKDRKYPTGTRTNRATAAGFWKATGRDKAVLSKNSVIGMRKTLVYYKGRAPNGRKSDWIMHEYRLQNSELAPVQEEGWVVCRAFRKPIPNQRPLGYEPWQNQLYHVESSNNYSSSVTMNTSHHIGASSSSHNLNQMLMSNNHYNPNNTSSSMHQYGNIELPQLDSPSLSPSLGTNKDQNESFEQEEEKSFNCVDWRTLDTLLETQVIHPHNPNILMFETQSYNPAPSFPSMHQSYNEVEANIHHSLGCFPDS

> ANAC031

MMLAVEDVLSELAGEERNERGLPPGFRFHPTDEELITFYLASKIFHGGLSGIHISEVDLNRCEPWELPEMAKMGEREWYFYSLRDRKYPTGLRTNRATTAGYWKATGKDKEVFSGGGGQLVGMKKTLVFYKGRAPRGLKTKWVMHEYRLENDHSHRHTCKEEWVICRVFNKTGDRKNVGLIHNQISYLHNHSLSTTHHHHHEALPLLIEPSNKTLTNFPSLLYDDPHQNYNNNNFLHGSSGHNIDELKALINPVVSQLNGIIFPSGNNNNDEDDFDFNLGVKTEQSSNGNEIDVRDYLENPLFQEASYGLLGFSSSPGPLHMLLDSPCPLGFQL

>ANAC032

MMKSGADLQFPPGFRFHPTDEELVLMYLCRKCASQPIPAPIITELDLYRYDPWDLPDMALYGEKEWYFFSPRDRKYPNGSRPNRAAGTGYWKATGADKPIGRPKPVGIKKALVFYSGKPPNGEKTNWIMHEYRLADVDRSVRKKNSLRLDDWVLCRIYNKKGVIEKRRSDIEDGLKPVTDTCPPESVARLISGSEQAVSPEFTCSNGRLSNALDFPFNYVDAIADNEIVSRLLGGNQMWSTTLDPLVVRQGTF

> ANAC033

MEIGSSSTVAGGGQLSVPPGFRFHPTEEELLYYYLKKKVSYEPIDLDVIREVDLNKLEPWELKEKCRIGSGPQNEWYFFSHKDKKYPTGTRTNRATAAGFWKATGRDKSIHLNSSKKIGLRKTLVFYTGRAPHGQKTEWIMHEYRLDDSENEIQEDGWVVCRVFKKKNHFRGFHQEQEQDHHHHHQYISTNNDHDHHHHIDSNSNNHSPLILHPLDHHHHHHHIGRQIHMPLHEFANTLSHGSMHLPQLFSPDSAAAAAAAAASAQPFVSPINTTDIECSQNLLRLTSNNNYGGDWSFLDKLLTTGNMNQQQQQQVQNHQAKCFGDLSNNDNNDQADHLGNNNGGSSSSPVNQRFPFHYLGNDANLLKFPK

>ANAC034 MAIVSSTTSIIPMSNQVNNNEKGIEDNDHRGGQESHVQNEDEADDHDHDMVMPGFRFHPTEEELIEFYLRRKVEGKRFNVELITFLDLYRYDPWELPAMAAIGEKEWYFYVPRDRKYRNGDRPNRVTTSGYWKATGADRMIRSETSRPIGLKKTLVFYSGKAPKGTRTSWIMNEYRLPHHETEKYQKAEISLCRVYKRPGVEDHPSVPRSLSTRHHNHNSSTSSRLALRQQQHHSSSSNHSDNNLNNNNNINNLEKLSTEYSGDGSTTTTTTNSNSDVTIALANQNIYRPMPYDTSNNTLIVSTRNHQDDDETAIVDDLQRLVNYQISDGATTLMPQTQAALAMNMIPAGTIPNNALWDMWNPIVPDGNRDHYTNIPFK

> ANAC035

MAIVSSTTSIIPMSNQVNNNEKGIEDNDHRGGQESHVQNEDEADDHDHDMVMPGFRFHPTEEELIEFYLRRKVEGKRFNVELITFLDLYRYDPWELPAMAAIGEKEWYFYVPRDRKYRNGDRPNRVTTSGYWKATGADRMIRSETSRPIGLKKTLVFYSGKAPKGTRTSWIMNEYRLPHHETEKYQKAEISLCRVYKRPGVEDHPSVPRSLSTRHHNHNSSTSSRLALRQQQHHSSSSNHSDNNLNNNNNINNLEKLSTEYSGDGSTTTTTTNSNSDVTIALANQNIYRPMPYDTSNNTLIVSTRNHQDDDETAIVDDLQRLVNYQISDGGNINHQYFQIAQQFHHTQQQNANANALQLVAAATTATTLMPQTQAALAMNMIPAGTIPNNALWDMWNPIVPDGNRDHYTNIPFK

>ANAC036

MGKDIELPGFRFHPTEEELLDFYLKNMVYGKRSSVEVIGFLNIYRHDPWDLPGLSRIGEREWYFFVPRERKHGNGGRPSRTTEKGYWKATGSDRKIISLSEPKRVIGLKKTLVFYRGRAPGGSKTDWVMNEFRMPDNCSLPKDVVLCKIYRKATSLKVLEQRAEMEAKMNQTCPNSPLSSSETISFVGKEENMMTSFRAPQVIAMEEANKIQMHQENAKTEEKQREAETKEPSSSLKLPFGSLPELQLPKPGVEWDQLLSISPWLQNLTPIVNIYW

> ANAC037

MEPMESCSVPPGFRFHPTDEELVGYYLRKKIASQKIDLDVIRDIDLYRIEPWDLQEQCRIGYEEQNEWYFFSHKDKKYPTGTRTNRATMAGFWKATGRDKAVYDKTKLIGMRKTLVFYKGRAPNGKKSDWIMHEYRLESDENAPPQEEGWVVCRAFKKRATGQAKNTETWSSSYFYDEVAPNGVNSVMDPIDYISKQQHNIFGKGLMCKQELEGMVDGINYIQSNQFIQLPQLQSPSLPLMKRPSSSMSITSMDNNYNYKLPLADEESFESFIRGEDRRKKKKQVMMTGNWRELDKFVASQLMSQEDNGTSSFAGHHIVNEDKNNNDVEMDSSMFLSEREEENRFVSEFLSTNSDYDIGICVFDN

>ANAC038

MEQGDHQQHKKEEEALPPGFRFHPTDEELISYYLVNKIADQNFTGKAIADVDLNKSEPWELPEKAKMGGKEWYFFSLRDRKYPTGVRTNRATNTGYWKTTGKDKEIFNSTTSELVGMKKTLVFYRGRAPRGEKTCWVMHEYRLHSKSSYRTSKQDEWVVCRVFKKTEATKKYISTSSSSTSHHHNNHTRASILSTNNNNPNYSSDLLQLPPHLQPHPSLNINQSLMANAVHLAELSRVFRASTSTTMDSSHQQLMNYTHMPVSGLNLNLGGALVQPPPVVSLEDVAAVSASYNGENGFGNVEMSQCMDLDGYWPSY

>ANAC039

MEQGDHQQHKKEEEALPPGFRFHPTDEELISYYLVNKIADQNFTGKAIADVDLNKSEPWELPEKAKMGGKEWYFFSLRDRKYPTGVRTNRATNTGYWKTTGKDKEIFNSTTSELVGMKKTLVFYRGRAPRGEKTCWVMHEYRLHSKSSYRTSKQDEWVVCRVFKKTEATKKYISTSSSSTSHHHNNHTRASILSTNNNNPNYSSDLLQLPPHLQPHPSLNINQSLMANAVHLAELSRVFRASTSTTMDSSHQQLMNYTHMPVSGLNLNLGGALVQPPPVVSLEDVAAVSASYNGENGFGNVEMSQCMDLDGYWPSY

>ANAC040

MSKEAEMSIAVSALFPGFRFSPTDVELISYYLRRKIDGDENSVAVIAEVEIYKFEPWDLPEESKLKSENEWFYFCARGRKYPHGSQSRRATQLGYWKATGKERSVKSGNQVVGTKRTLVFHIGRAPRGERTEWIMHEYCIHGAPQDALVVCRLRKNADFRASSTQKMEDGVVQDDGYVGQRGGLEKEDKSYYESEHQIPNGDIAESSNVVEDQADTDDDCYAEILNDDIIKLDEEALKASQAFRPTNPTHQETISSESSSKRSKCGIKKESTETMNCYALFRIKNVAGTDSSWRFPNPFKIKKDDSQRLMKNVLATTVFLAILFSFFWTVLIARN

>ANAC041

MEKRSSIKNRGVLRLPPGFRFHPTDEELVVQYLRRKVTGLPLPASVIPETDVCKSDPWDLPGDCESEMYFFSTREAKYPNGNRSNRSTGSGYWKATGLDKQIGKKKLVVGMKKTLVFYKGKPPNGTRTNWVLHEYRLVDSQQDSLYGQNMNWVLCRVFLKKRSNSNSKRKEDEKEEVENEKETETEREREEENKKSTCPIFYDFMRKDTKKKRRRRRCCDLNLTPATCCCCSSSTSSSSVCSSALTHTSSNDNRQEISYRENKFCLFL

>ANAC042

MSGEGNLGKDHEEENEAPLPGFRFHPTDEELLGYYLRRKVENKTIKLELIKQIDIYKYDPWDLPRVSSVGEKEWYFFCMRGRKYRNSVRPNRVTGSGFWKATGIDKPVYSNLDCVGLKKSLVYYLGSAGKGTKTDWMMHEFRLPSTTKTDSPAQQAEVWTLCRIFKRVTSQRNPTILPPNRKPVITLTDTCSKTSSLDSDHTSHRTVDSMSHEPPLPQPQNPYWNQHIVGFNQPTYTGNDNNLLMSFWNGNGGDFIGDSASWDELRSVIDGNTKP

> ANAC043

MMSKSMSISVNGQSQVPPGFRFHPTEEELLQYYLRKKVNSIEIDLDVIRDVDLNKLEPWDIQEMCKIGTTPQNDWYFFSHKDKKYPTGTRTNRATAAGFWKATGRDKIIYSNGRRIGMRKTLVFYKGRAPHGQKSDWIMHEYRLDDNIISPEDVTVHEVVSIIGEASQDEGWVVCRIFKKKNLHKTLNSPVGGASLSGGGDTPKTTSSQIFNEDTLDQFLELMGRSCKEELNLDPFMKLPNLESPNSQAINNCHVSSPDTNHNIHVSNVVDTSFVTSWAALDRLVASQLNGPTSYSITAVNESHVGHDHLALPSVRSPYPSLNRSASYHAGLTQEYTPEMELWNTTTSSLSSSPGPFCHVSNGSG

>ANAC044

MARAWIVDGRGIAAKVKNASLSSALQIQDCGAHIKCPNCTYRIDNSNVLIPWPGLPKGVKFEPTDEDIIEFLEAKCGIGGSEPHVLIEEFIRPVTEDVGINYTHPQNLPGANKDGVSVFFFHKTVQAYGTGQRKRRKITPTLVNDEPVRWHKTGRTKPVMLSGVQRGCKKIMVLYKSARKGTKPEKSNWVLHQYHLGTEGKEIGDYVVSKITYQQQKLGENPDEGESSSGVRGGPTTPKTNTPTPPSLVDGVAGDEEAFDDLKMFDPFFEELDSIPEAALGKMWSKKARMDEEFVVNLSEDNLICDESMEASSLWENQVLPNPSLGTVGDFDGFSISDLENADLGTPPDFLTVRSCLKKTLFTFGKFNGT

>ANAC045

MAPVSLPPGFRFHPTDEELITYYLKRKINGLEIELEVIAEVDLYKCEPWDLPGKSLLPSKDQEWYFFSPRDRKYPNGSRTNRATKGGYWKATGKDRRVSWRDRAIGTKKTLVYYRGRAPHGIRTGWVMHEYRLDETECEPSAYGMQDAYALCRVFKKIVIEAKPRDQHRSYVHAMSNVSGNCSSSFDTCSDLEISSTTHQVQNTFQPRFGNERFNSNAISNEDWSQYYGSSYRPFPTPYKVNTEIECSMLQHNIYLPPLRVENSAFSDSDFFTSMTHNNDHGVFDDFTFAASNSNHNNSVGDQVIHVGNYDEQLITSNRHMNQTGYIKEQKIRSSLDNTDEDPGFHGNNTNDNIDIDDFLSFDIYNEDNVNQIEDNEDVNTNETLDSSGFEVVEEETRFNNQMLISTYQTTKILYHQVVPCHTLKVHVNPISHNVEERTLFIEEDKDSWLQRAEKITKTKLTLFSLMAQQYYKCLAIFF

>ANAC046

MVEEGGVVVNQGGDQEVVDLPPGFRFHPTDEEIITHYLKEKVFNIRFTAAAIGQADLNKNEPWDLPKIAKMGEKEFYFFCQRDRKYPTGMRTNRATVSGYWKATGKDKEIFRGKGCLVGMKKTLVFYTGRAPKGEKTNWVMHEYRLDGKYSYHNLPKTARDEWVVCRVFHKNAPSTTITTTKQLSRIDSLDNIDHLLDFSSLPPLIDPGFLGQPGPSFSGARQQHDLKPVLHHPTTAPVDNTYLPTQALNFPYHSVHNSGSDFGYGAGSGNNNKGMIKLEHSLVSVSQETGLSSDVNTTATPEISSYPMMMNPAMMDGSKSACDGLDDLIFWEDLYTS

>ANAC047

MISKDPRSSLPPGFRFHPTDEELILHYLRKKVSSSPVPLSIIADVDIYKSDPWDLPAKAPFGEKEWYFFSPRDRKYPNGARPNRAAASGYWKATGTDKLIAVPNGEGFHENIGIKKALVFYRGKPPKGVKTNWIMHEYRLADSLSPKRINSSRSGGSEVNNNFGDRNSKEYSMRLDDWVLCRIYKKSHASLSSPDVALVTSNQEHEENDNEPFVDRGTFLPNLQNDQPLKRQKSSCSFSNLLDATDLTFLANFLNETPENRSESDFSFMIGNFSNPDIYGNHYLDQKLPQLSSPTSETSGIGSKRERVDFAEETINASKKMMNTYSYNNSIDQMDHSMMQQPSFLNQELMMSSHLQYQG

>ANAC048

MENPVGLRFRPTDKEIVVDYLRPKNSDRDTSHVDRVISTVTIRSFDPWELPCQSRIKLKDESWCFFSPKENKYGRGDQQIRKTKSGYWKITGKPKPILRNRQEIGEKKVLMFYMSKELGGSKSDWVMHEYHAFSPTQMMMTYTICKVMFKGDVREISSSSASYGSEIEQSRDSLIPLLVNDSEEEAQIEDAIPIEEWETWLTDDGVDEQVNHIMNMKDDRNNHRPQKPLTGVLIDDSSDDDDDSDLLSPTTNSIENSSTCDSFGSSDQINLVSLTQEVSQALITSIDTPEKIKSPYDDAQGTGAGGQKLGQETREKKRAGFFHRMIQIFVKKIHQCSSISRT

>ANAC049 MYRCTFNPPEEELINYYLNNKITENDDLVGKQIAEVNILHHEPADLPGLAKIESSHTWYFISPVEKFGKLNRTKRVSRSGHWKITGNSRTIKDVDGNPIGLKKFLVFQENKRSSSSSLSTTTTQQQKTNWIIHEFHSFLPHPNKMLTENKNEDYGGDYCNGLDELLQSLEQGESSGLLFGENGHACEQDTAENSNVTM

>ANAC050

MGRESLAVVSSPPSATAPSTAVSATSLAPGFRFHPTDEELVSYYLKRKVLGKPVRFDAIGEVDIYKHEPWDLAEKILCSYLALNCFKNFVFACATVMFACATVMFALFSKLKTRDQEWYFFSALDKKYGNGARMNRATNKGYWKATGKDREIRRDIQLLGMKKTLVFHSGRAPDGLRTNWVMHEYRLVEYETETNGSLLQDAYVLCRVFHKNNIGPPSGNRYAPFMEEEWADGGGALIPGIDVRVRVEALPQANGNNQMDQWADLLKLHNSIKFAITFCRTQLNLTALSNERCSTREIFIVFWLICKEMHSASKDLININELPRDATPMDIEPNQQNHHESAFKPQESNNHSGYEEDEDTLKREHAEEDERPPSLCILNKEAPLPLLQYKRRRQNESNNNSSRNTQDHCSSTITTVDNTTTLISSSAAAATNTAISALLEFSLMGISDKKENQQKEETSPPSPIASPEEKVNDLQKEVHQMSVERETFKLEMMSAEAMISILQSRIDALRQENEELKKKNASGQAS

>ANAC051

MGRESVAVVTAPPSATAPGTASVATSLAPGFRFHPTDEELVSYYLKRKVLGQPVRFDAIGEVDIYKHEPWDLAVFSRLKTRDQEWYFYSALDKKYGNGARMNRATNRGYWKATGKDREIRRDILLLGMKKTLVFHSGRAPDGLRTNWVMHEYRLVEYETEKNGNLVQDAYVLCRVFHKNNIGPPSGNRYAPFMEEEWADDEGALIPGIDVKLRLEPPPVANGNDQMDQIIYASSGVHL

>ANAC052

MGRESVAVVTAPPSATAPGTASVATSLAPGFRFHPTDEELVSYYLKRKVLGQPVRFDAIGEVDIYKHEPWDLAVFSRLKTRDQEWYFYSALDKKYGNGARMNRATNRGYWKATGKDREIRRDILLLGMKKTLVFHSGRAPDGLRTNWVMHEYRLVEYETEKNGNLVQDAYVLCRVFHKNNIGPPSGNRYAPFMEEEWADDEGALIPGIDVKLRLEPPPVANGNDQMDQEIQSASKSLININEPPRETAPLDIESDQQNHHENDLKPEEHNNNNNYDENEETLKREQMEEEERPPRPVCVLNKEAPLPLLQYKRRRQSESNNNSSRNTQDHCSSTTTTVDNTTTLISSSAAATNTAISALLEFSLMGISDKKEKPQQPLRPHKEPLPPQTPLASPEEKVNDLQKEIHQMSVERETFKLEMMSAEAMISILQSRIDALRQENEELKKNNANGQ

>ANAC053

MGRGSVTSLAPGFRFHPTDEELVRYYLKRKICNKPFKFDAISVTDVYKSEPWDLPDKSRLKSRDLEWYFFSMLDKKYRNGSKTNRATEMGYWKTTGKDREILNGSKVVGMKKTLVYHKGRAPRGERTNWVMHEYRLVDQDLDKTGVHQDAFVLCRIFQKSGSGPKNGEQYGAPFVEEEWEEEDDMTFVPDQEDLGSEDHVYVHMDDIDQKSENFVVYDAIPIPLNFIHGESSNNVETNYSDSINYIQQTGNYMDSGGYFEQPAESYEKDQKPIIRDRDGSLQNEGIGCGVQDKHSETLQSSDNIFGTDTSCYNDFPVESNYLIGEAFLDPNSNLLENDGLYLETNDLSSTQQDGFDFEDYLTFFDETFDPSQLMGNEDVFFDQEELFQEVETKELEKEETSRSKHVVEEKEKDEASCSKQVDADATEFEPDYKYPLLKKASHMLGAIPAPLANASEFPTKDAAIRLHAAQSSGSVHVTAGMITISDSNMGWSYGKNENLDLILSLGLVQGNTAPEKSGNSSAWAMLIFMCFWVLLLSVSFKVSILVSSR

> ANAC054

MDVDVFNGWGRPRFEDESLMPPGFRFHPTDEELITYYLLKKVLDSNFSCAAISQVDLNKSEPWELPEKAKMGEKEWYFFTLRDRKYPTGLRTNRATEAGYWKATGKDREIKSSKTKSLLGMKKTLVFYKGRAPKGEKSCWVMHEYRLDGKFSYHYISSSAKDEWVLCKVCLKSGVVSRETNLISSSSSSAVTGEFSSAGSAIAPIINTFATEHVSCFSNNSAAHTDASFHTFLPAPPPSLPPRQPRHVGDGVAFGQFLDLGSSGQIDFDAAAAAFFPNLPSLPPTVLPPPPSFAMYGGGSPAVSVWPFTL

>ANAC055

MGLQELDPLAQLSLPPGFRFYPTDEELMVEYLCRKAAGHDFSLQLIAEIDLYKFDPWVLPSKALFGEKEWYFFSPRDRKYPNGSRPNRVAGSGYWKATGTDKVISTEGRRVGIKKALVFYIGKAPKGTKTNWIMHEYRLIEPSRRNGSTKLDDWVLCRIYKKQTSAQKQAYNNLMTSGREYSNNGSSTSSSSHQYDDVLESLHEIDNRSLGFAAGSSNALPHSHRPVLTNHKTGFQGLAREPSFDWANLIGQNSVPELGLSHNVPSIRYGDGGTQQQTEGIPRFNNNSDVSANQGFSVDPVNGFGYSGQQSSGFGFI

> ANAC056

MESTDSSGGPPPPQPNLPPGFRFHPTDEELVVHYLKRKAASAPLPVAIIAEVDLYKFDPWELPAKASFGEQEWYFFSPRDRKYPNGARPNRAATSGYWKATGTDKPVLASDGNQKVGVKKALVFYSGKPPKGVKSDWIMHEYRLIENKPNNRPPGCDFGNKKNSLRLDDWVLCRIYKKNNASRHVDNDKDHDMIDYIFRKIPPSLSMAAASTGLHQHHHNVSRSMNFFPGKFSGGGYGIFSDGGNTSIYDGGGMINNIGTDSVDHDNNADVVGLNHASSSGPMMMANLKRTLPVPYWPVADEEQDASPSKRFHGVGGGGGDCSNMSSSMMEETPPLMQQQGGVLGDGLFRTTSYQLPGLNWYSS

>ANAC057

MAPVGLPPGFRFHPTDEELVNYYLKRKINGQEIELDIIPEVDLYKCEPWDLAEKSFLPSRDPEWYFFGPRDRKYPNGFRTNRATRGGYWKSTGKDRRVTSQSRAIGMKKTLVYYKGRAPQGIRTDWVMHEYRLDDKDCDDPSSLQDSYALCRVFKKNGICSELESERQLQTGQCSFTTASMEEINSNNNNNYNNDYETMSPEVGVSSACVEEVVDDKDDSWMQFITDDAWDTSSNGAAMGHGQGVY

>ANAC058

MEENLPPGFRFHPTDEELITHYLCRKVSDIGFTGKAVVDVDLNKCEPWDLPAKASMGEKEWYFFSQRDRKYPTGLRTNRATEAGYWKTTGKDKEIYRSGVLVGMKKTLVFYKGRAPKGEKSNWVMHEYRLESKQPFNPTNKEEWVVCRVFEKSTAAKKAQEQQPQSSQPSFGSPCDANSSMANEFEDIDELPNLNSNSSTIDYNNHIHQYSQRNVYSEDNTTSTAGLNMNMNMASTNLQSWTTSLLGPPLSPINSLLLKAFQIRNSYSFPKEMIPSFNHSSLQQGVSNMIQNASSSSQVQPQPQEEAFNMDSIW

> ANAC059

MDYKVSRSGEIVEGEVEDSEKIDLPPGFRFHPTDEELITHYLRPKVVNSFFSAIAIGEVDLNKVEPWDLPWKAKLGEKEWYFFCVRDRKYPTGLRTNRATKAGYWKATGKDKEIFKGKSLVGMKKTLVFYKGRAPKGVKTNWVMHEYRLEGKFAIDNLSKTAKNECVISRVFHTRTDGTKEHMSVGLPPLMDSSPYLKSRGQDSLAGTTLGGLLSHVTYFSDQTTDDKSLVADFKTTMFGSGSTNFLPNIGSLLDFDPLFLQNNSSVLKMLLDNEETQFKKNLHNSGSSESELTASSWQGHNSYGSTGPVNLDCVWKF

>ANAC060

MAAAPPIEPAVTTTFPGFKFSPTDIELISYYLKRKMDGLERSVEIIPEVEIYNFEPWDLPDKSIVKSDSEWFFFCARGKKYPHGSQNRRATKIGYWKATGKERNVKSGSEVIGTKRTLVFHIGRAPKGGRTEWLMHEYCMIGVSLDALVICRLRRNTEFQGSTIQKPPQPSLPLDKHVNLRNEAISESIYGWETMVDFYLSSESGQELLSEIAESSQSSQNPQVPSEEDFYADILRDEIVKLDDPAVSGNTLINVPRLQTESNTTRVLPLPDMVDKQMQSLLQKLPLQNDTGEENNISMSNCFIGIYSIKSINRARWDVVVWLLVMIAVLVFYLV

>ANAC061

MGEELSVGFRFYPTEVELLTYYLRIQLGGGNATIHSLIPILDVFSVEPTQLPNLAGERCRGDAEQWIFFVPRQEREARGGRPSRTTGSGYWKATGSPGPVFSPDNRVIGVKKTMVFYTGKAPTGRKTKWKMNEYKAVETASVSTIPKLRPEFSICRIYIKSGSSRAFDRRPTEAYAIERNLPSNGVETSSRATISTSPETSHSGGNQVDLPVNATTITQSISDMVDELSQPFWEWEQMNWS

> ANAC062

MNQNLHVLSMDSLPVGLRFRPTDEELIRYYLRRKINGHDDDVKAIREIDICKWEPWDLPDFSVIKTKDSEWLYFCPLDRKYPSGSRQNRATVAGYWKATGKDRKIKSGKTNIIGVKRTLVFHAGRAPRGTRTNWIIHEYRATEDDLSGTNPGQSPFVICKLFKKEELVLGEEDSKSDEVEEPAVSSPTVEVTKSEVSEVIKTEDVKRHDIAESSLVISGDSHSDACDEATTAELVDFKWYPELESLDFTLFSPLHSQVQSELGSSYNTFQPGSSNFSGNNNNSFQIQTQYGTNEVDTYISDFLDSILKSPDEDPEKHKYVLQSGFDVVAPDQIAQVCQQGSAVDMSNDVSVTGIQIKSRQAQPSGYTNDYIAQGNGPRRLRLQSNFNGINTKNPELQAIKREAEDTVGESIKKRCGKLMRSKNVTGFVFKKITSVKCSYGGLFRAAVVAVVFLMSVCSLTVDFRASAVS

>ANAC063

MSPPSTIAYVLPPGFKFVPNDEEVIHCYLKPYSDGNTNVLLHVPIHLVNIYESNPQTLSEEFQKGNDKEWFIITERNKVDQGLSQTKRVGYGAKRQKRVDTNGGYWHATVAAQKINAGDGVVRNKRPLAYYVGKPSEGVKTDWLMQEYSLDHSSHNNDKDYTLCKIYLTPQATKMNKEVGEEKKKQKKGEAVVSVAPVEALEEQLPCNVEYHQPLAPLDSCQPQPHDLAYQQQQFCPGPLDSYQPQPHDMENQQPHNEKLKKEEDVEQLDLHQPDQGKGC

>ANAC064

MAIPQRNKRKARSSPERLTQPPELPHNSDVPSSSSSSAADNFFSWSTKQFAFPPGYRFVPKDQELIFHYLKPFSQGNKCSLLNVPIHRVNIYESNPQHLSEKYEKGNDKDWFYISERTKTGKAGRSNKRVDNGGYWSATVAAQKINAGNGIVGYKTSLEYYVGKQSNSVKGDWLMQEYWFESSDDNNNEKVDHALCKIYLTPAAAKKKKAEEAENEKLKKEEDVEQLDLNQPDQLQLQQPHDIVYQPQYCLLPEHHQPQPFPDNFSELISFQQQPVMIPDDFEDFLAEFTKPHSLDGDEEFNNYGLFEGFFDTEGMIKH

>ANAC065

MFDFKIFTFSFAQSKYLENIREKTLTEVSTSDIELLRRNSQPFRRATPCSLTVDLQANSFTPKLQSYLMMMPGRNKRKERSWPEPQTQPQSEIPSSSSSLAADNMSPPSTIAYVLPPGFKFMPNDKEVINCYLKPYSDGNTNVLLNVPIHRVNIYESNPQTLSGRNVATLTEDIGTQKWLPKRFKAGDGVVGNKRPLAYYVGKPSEGVKTDWLMQEYSLDHSSHNTTR

> ANAC066

MNISVNGQSQVPPGFRFHPTEEELLKYYLRKKISNIKIDLDVIPDIDLNKLEPWDIQEMCKIGTTPQNDWYFYSHKDKKYPTGTRTNRATTVGFWKATGRDKTIYTNGDRIGMRKTLVFYKGRAPHGQKSDWIMHEYRLDESVLISSCGDHDVNVETCDVIGSDEGWVVCRVFKKNNLCKNMISSSPASSVKTPSFNEETIEQLLEVMGQSCKGEIVLDPFLKLPNLECHNNTTITSYQWLIDDQVNNCHVSKVMDPSFITSWAALDRLVASQLNGPNSYSIPAVNETSQSPYHGLNRSGCNTGLTPDYYIPEIDLWNEADFARTTCHLLNGSG

>ANAC067

MMKDPTGYRFSPTGEEVINHYLKNKILGKTWLVDEAISEINILNHKPSKDLPKLARIQSEDLEWYFFSPIEYTNPNKMKMKRTTGSGFWKPSGVDRKIRDKRGNGVVIGIKKTLVYHEGKSPHGVRTPWVMHEYHITCLPHHKRKYVVCQVKYKGEAAEISYEPSPSLVSDSHTVIAINGEPEPELQVEQPGKENLLGMSVDDLIEPMNQQEEPQGPHLAPNDDEFIRGLRHVDREPVEYLFANEENMDGLSIMNDLTIPMIAQQEDLILSEWEGFIAATFFSDNNNNNNLNVHQLTSFLPG

>ANAC068

MMKGLIGYRFSPTGEEVINHYLKNKLLGKYWLVDEAISEINILSHKPSKDLPKLARIQSEDLEWYFFSPIEYTNPNKMKMKRTTGSGFWKPTGVDREIRDKRGNGVVIGIKKTLVYHEGKSPHGVRTPWVMHEYHITCLPHHKRKYVVCQVKYKGEAAEISYEPSPSLVSDSHTVIAITGEPEPELQVEQPGKENLLGMSVDDLIEPMNQQEEPQGPHLAPNDDEFIRGLRHVDRGTVEYLFANEENMDGLSMNDLRIPMIVQQEDLSEWEGFNADTFFSDNNNNYNLNVHHQLTPYGDGYLNAFSGYNEGNPPDHELVMQENRNDHMPRKPVTGTIDYSSDSGSDAGSISTTSYQGTSSPNISVGSSSRHLSSCSSTDSCKDLQTCTDPSIISREIRELTQEVKQEIPRAVDAPMNNESSLVKTEKKGLFIVEDAMERNRKKPRFIYLMKMIIGNIISVLLPVKRLIPVKKL

>ANAC069

MVKDLVGYRFYPTGEELINHYLKNKILGKTWLVDEAISEINICSYDPIYLPSLSKIKSDDPVWYFFCPKEYTSAKKKVTKRTTSSGYWKATGVDRKIKDKRGNRGEIGIKKTLVYYEGRVPKGVWTPWVMHEYHITCLPQDQRNYVICQVMYKGEDGDVPSGGNNSSEPSQSLVSDSNTVRATSPTALEFEKPGQENFFGMSVDDLGTPKNEQEDFSLWDVLDPDMLFSDNNNPTVHPQAPHLTPNDDEFLGGLRHVNREQVEYLFANEDFISRPTLSMTENRNDHRPKKALSGIIVDYSSDSNSDAESISATSYQGTSSPGDDSVGSSNRQFLQTGGDEILSSCNDLQTYGEPSISSSTRQSQLTRSIIRPKQEVKQDTSRAVDSDTSIDKESSMVKTEKKSWFITEEAMERNRNNPRYIYLMRMIIGFILLLALISNIISVLQNLNPAMKFDRER

>ANAC070

MGSSSNGGVPPGFRFHPTDEELLHYYLKKKISYQKFEMEVIREVDLNKLEPWDLQERCKIGSTPQNEWYFFSHKDRKYPTGSRTNRATHAGFWKATGRDKCIRNSYKKIGMRKTLVFYKGRAPHGQKTDWIMHEYRLEDADDPQANPSEDGWVVCRVFMKKNLFKVVNEGSSSINSLDQHNHDASNNNHALQARSFMHRDSPYQLVRNHGAMTFELNKPDLALHQYPPIFHKPPSLGFDYSSGLARDSESAASEGLQYQQACEPGLDVGTCETVASHNHQQGLGEWAMMDRLVTCHMGNEDSSRGITYEDGNNNSSSVVQPVPATNQLTLRSEMDFWGYSK

>ANAC071

MGSSCLPPGFRFHPTDEELIGYYLSRKIEGLEIELEVIPVIDLYKFDPWELPGKSFLPNRDLEWFFFCPRDKKYANGSRTNRATKAGYWKATGKDRKITCKSSHVIAGYRKTLVFYEGRAPLGDRTNWFMHEYRLCDIDDHSQKSPNFKGAFALCRVVKKNELKKNSKSLKNKNEQDIGSCYSSLATSPCRDEASQIQSFKPSSTTNDSSSIWISPDFILDSSKDYPQIKEVASECFPNYHFPVTTANHHVEFPLQEMLVRS

> ANAC72

MGVREKDPLAQLSLPPGFRFYPTDEELLVQYLCRKVAGYHFSLQVIGDIDLYKFDPWDLPSKQTCFTFVGEYNCNYLGKALFGEKEWYFFSPRDRKYPNGSRPNRVAGSGYWKATGTDKIITADGRRVGIKKALVFYAGKAPKGTKTNWIMHEYRLIEHSRSHGSSKLDDWVLCRIYKKTSGSQRQAVTPVQACREEHSTNGSSSSSSSQLDDVLDSFPEIKDQSFNLPRMNSLRTILNGNFDWASLAGLNPIPELAPTNGLPSYGGYDAFRAAEGEAESGHVNRQQNSSGLTQSFGYSSSGFGVSGQTFEFRQ

>ANAC073

MTWCNDRSDVQTVERIIPSPGAAESPVASLPVSCHKTCPSCGHNFKFHEQAGIHDLPGLPAGVKFDPTDQEVLEHLEGKVRDDAKKLHPLIDEFIRTIDGENGICYTHPEKLPGVNKDGTVRHFFHRPSKAYTTGTRKRRKVHTDSDVGGETRWHKTGKTRPVLAGGRVRGYKKILVLYTNYGKQKKPEKTNWVMHQYHLGTSEEEKEGELVVSKVFYQTQPRQCGGSVAAAATAKDRPYLHGLGGGGGRHLHYHLHHNNGNGKSNGSGGTAGAGEYYHNIPAIISFNQTGIQNHLVHDSQPFIP

>ANAC074

MGLKDIGSKLPPGFRFHPSDEELVCHYLCNKIRAKSDHGDVDDDDDDVDEALKGSTDLVEIDLHICEPWELPDVAKLNAKEWYFFSFRDRKYATGYRTNRATVSGYWKATGKDRTVMDPRTRQLVGMRKTLVFYRNRAPNGIKTTWIMHEFRLECPNIPPKEDWVLCRVFNKGRDSSLQDNNYYNNDNQTQRLEVNDAPDLNYNNQLPPLLSSPPHNHQHEKMKIQVCDQWEQLMKQPSRTTGHPYHHHCHHQTIACGWEQMMIGSLSSPSSHGPDHESLLNLLYVDNNNSVNISGDHHQNYEKILLSSLDMTSLDHDKTCMGSSSDGGMVSDLHMECGGLSFETENILAFQ

>ANAC075

MNKSNPAGSVTGSDIIDAKIEEHQLCGSKKCPSCGHKLEGKPQDWVGLPAGVKFDPTDQELIEHLEAKVLAKDFKSHPLIDEFIPTIEGEDGICYTHPEKLPGVTRDGLSRHFFHRPSKAYTTGTRKRRKIQTECDNNLQGSSSSGETRWHKTGKTRPVMVNGKQKGCKKILVLYTNFGKNRKPEKTNWVMHQYHLGTHEEEKEGELVVSKIFYQTQPRQCNWSSSTSSLNAIGGGGGEASSGGGGGEYHMRRDSGTTSGGSCSSSREIINVNPPNRSDEIGGVGGGVMAVAAAAAAVAAGLPSYAMDQLSFVPFMKSFDEVARRETPQTGHATCEDVMAEQHRHRHQPSSSTSHHMAHDHHHHHHQQQQQRHHAFNISQPTHPISTIISPSTSLHHASINILDDNPYHVHRILLPNENYQTQQQLRQEGEEEHNDGKMGGRSASGLEELIMGCTSSTTHHDVKDGSSSMGNQQEAEWLKYSTFWPAPDSSDNQDHHG

>ANAC076_VND2

MESVDQSCSVPPGFRFHPTDEELVGYYLRKKVASQKIDLDVIRDIDLYRIEPWDLQESCRIGYEERNEWYFFSHKDKKYPTGTRTNRATMAGFWKATGRDKAVYDKSKLIGMRKTLVFYKGRAPNGQKTDWIMHEYRLESDENAPPQEEGWVVCRAFKKKPMTGQAKNTETWSSSYFYDELPSGVRSVTEPLNYVSKQKQNVFAQDLMFKQELEGSDIGLNFIHCDQFIQLPQLESPSLPLTKRPVSLTSITSLEKNKNIYKRHLIEEDVSFNALISSGNKDKKKKKTSVMTTDWRALDKFVASQLMSQEDGVSGFGGHHEEDNNKIGHYNNEESNNKGSVETASSTLLSDREEENRFISGLLCSNLDYDLYRDLHV

>ANAC077

MARDSKKAGGALPAPATAMGNAAAETSLPPGFRFHPSDEELISYYLKKKVQGKPMRYDEIGEVDICKLEPWDLAVIIFLCFLALDFRYVLKTRDKEWFFFSALDKKTRTGTSMSRATKQGYWKVTGTDGKIRQGGDGKVTIGTMKTLVFHRGRSPNGLGTDWVMNEYHLAKNDEGVPQNNTGSRYAPFLEEEWDDDNGERVAIHVPDDGPLPLCVLNKEAPLPLIQYKRKRRISSSQTTQDHRSFTETIIDSTASAEPLDISERIALRALNGMLDDLEKEQEPVVVDGNKINEIQQESQLQRKKLIDLNLKEDAPSPLCVVNKETPSPLKYMIDDLEKEQEPATKRINDLVLKENDEIVVFREMQERESMKAEMEISFLEAQIDALDRKIDHPHK

>ANAC078

MGRGSVTSLAPGFRFHPTDEELVRYYLKRKVCNKPFKFDAISVTDIYKSEPWDLPDKSKLKSRDLEWYFFSMLDKKYSNGSKTNRATEKGYWKTTGKDREIRNGSRVVGMKKTLVYHKGRAPRGERTNWVMHEYRLSDEDLKKAGVPQEAYVLCRIFQKSGTGPKNGEQYGAPYLEEEWEEDGMTYVPAQDAFSEGLALNDDVYVDIDDIDEKPENLVVYDAVPILPNYCHGESSNNVESGNYSDSGNYIQPGNNVVDSGGYFEQPIETFEEDRKPIIREGSIQPCSLFPEEQIGCGVQDENVVNLESSNNNVFVADTCYSDIPIDHNYLPDEPFMDPNNNLPLNDGLYLETNDLSCAQQDDFNFEDYLSFFDDEGLTFDDSLLMGPEDFLPNQEALDQKPAPKELEKEVAGGKEAVEEKESGEGSSSKQDTDFKDFDSAPKYPFLKKTSHMLGAIPTPSSFASQFQTKDAMRLHAAQSSGSVHVTAGMMRISNMTLAADSGMGWSYDKNGNLNVVLSFGVVQQDDAMTASGSKTGITATRAMLVFMCLWVLLLSVSFKIVTMVSAR

>ANAC079

METFGVFHKEDDEQMDLPPGFRFHPTDEELITHYLHKKVLDLGFSAKAIGEVDLNKAEPWELPYKAKIGEKEWYFFCVRDRKYPTGLRTNRATQAGYWKATGKDKEIFRGKSLVGMKKTLVFYRGRAPKGQKTNWVMHEYRLDGKLSAHNLPKTAKNEWVICRVFHKTAGGKKIPISTLIRIGSYGTGSSLPPLTDSSPY

NDKTKTEPVYVPCFSNQAETRGTILNCFSNPSLSSIQPDFLQMIPLYQPQSLNISESSNPVLTQEQSVLQAMMENNRRQNFKTLSISQETGVSNTDNSSV

FEFGRKRFDHQEVPSPSSGPVDLEPFWNY

>ANAC080

MDLPPGFRFHPTDEELITHYLHKKVLDLGFSAKAIGEVDLNKAEPWELPYKAKIGEKEWYFFCVRDRKYPTGLRTNRATQAGYWKATGKDKEIFRGKSLV

GMKKTLVFYRGRAPKGQKTNWVMHEYRLDGKLSAHNLPKTAKNEWVICRVFHKTAGGKKIPISTLIRIGSYGTGSSLPPLTDSSPYNDKTKTEPVYVPCF

SNQAETRGTILNCFSNPSLSSIQPDFLQMIPLYQPQSLNISESSNPVLTQEQSVLQAMMENNRRQNFKTLSISQETGVSNTDNSSVFEFGRKRFDHQEVP

SPSSGPVDLEPFWNY

>ATAF2_ANAC081

MKSELNLPAGFRFHPTDEELVKFYLCRKCASEQISAPVIAEIDLYKFNPWELPEMSLYGEKEWYFFSPRDRKYPNGSRPNRAAGTGYWKATGADKPIGKP

KTLGIKKALVFYAGKAPKGIKTNWIMHEYRLANVDRSASVNKKNNLRLDDWVLCRIYNKKGTMEKYFPADEKPRTTTMAEQSSSPFDTSDSTYPTLQEDD

SSSSGGHGHVVSPDVLEVQSEPKWGELEDALEAFDTSMFGSSMELLQPDAFVPQFLYQSDYFTSFQDPPEQKPFLNWSFAPQG

>ANAC082

MGKTQLAPGFRFHPTDVELVRYYLKRKILGKKLLVDAIAEVDIYKFEPPDLPDMSFIRSGDLKWHFFCPREKKYASGVRANRATECGYWKTTGKERPVLCNSEVVGKIKTLVYHFGKSPRGERTDWVMHEYRLDDKVLTQMNVPQDTYVVCVLFKKDGPGPRNGAQYGAPFKEEDWSDEEVRTDVPSTSNPTNLLEPSKETTLALTAPDDSNKDCFGGMISESCVSDFLPATTNTTSELPHPSDAATTPMSTAPLAETVQTPNNDDLYSMLDLFDDDEEFLGFNNNEVRYDPGVSAPVCLEEEGIFNGLPELSSMPRTASYDLVENSELYLELQDLTAPLNPQTGLQDLTAPFNPQTGLQDLTAPFNHQTGLQDHTAPFNPQTGLQDHTAPFNHQTGLQDLTAPFNPQTGLQDLTAPFNPQTGLHDLTSPFNPQTGLQDLTAPLNPQTGNRNDPRSSSFLYNQGHFDFSGGNDDDPYGFSASMRHRPKM

>ANAC083

MDNVKLVKNGVLRLPPGFRFHPTDEELVVQYLKRKVCSSPLPASIIPEFDVCRADPWDLPGNLEKERYFFSTREAKYPNGNRSNRATGSGYWKATGIDKRVVTSRGNQIVGLKKTLVFYKGKPPHGSRTDWIMHEYRLSSSPPSSMGPTQNWVLCRIFLKKRAGNKNDDDDGDSRNLRHNNNNNSSDQIEIITTDQTDDKTKPIFFDFMRKERTTDLNLLPSSPSSDHASSGVTTEIFSSSDEETSSCNSFR

>ANAC084

MEVEKRIVVNGGMKLPIGYRFHPTEQELILHYLLPKAFASPLPSSIIPVFDLFFSHPLSFPGDQKEKQRYFFCKKREVSSNEHRIKISSGDGYWKPIGKERPIIACGKTFGIRRTLAFYETNKSSSYCNKTRWSMTEYCLAGFASAKVSGEWAVYNVYERKGSKGRKQRKSREGDDEELRCIDHFTVGSNHETGPPPPSPPTSADE

>ANAC085

MKTLHRTWIVDGPWISRNVKNASLSSALQIKDCGAYINCFNCSYRIDNSNVLTPWPGLPKGVKFEPTDEEVIEHLEAKCGIDGLKPHLLIQDFICSVTQDVGINYTHPQNLPGVSKDGTSVFFFNKTAHAYQNGQRKRRRITPTSLKDDTVRWHKTGQTKPVMLNGIQKGCKKIMVLYKSARKGFKPEKSNWVLHQYHLGTEEGEIGEYVVSKITYQQPKQQEKTIDESESSGVRGGPSTPKTSTITQVRPVISVDEDEIAFDDDSKMVLDSYAEGLENIQEASSGSTSDKIAKVGGNVSVIEDNLMSKKIEASSIPNHGNVDYGSGNFSVSDLENAELGTLPDLLSVRT

>ANAC086

MAPVSLPPGFRFHPTDEELITYYLKRKINGQEIELEIIPEVDLYKCEPWDLPGKSLIPSKDQEWFFFSPRDRKYPNGSRTNRATKGGYWKATGKDRRVSWRDRAIGTKKTLVYYRGRAPHGIRTGWVMHEYRLDESECEPSAFGMQDAYALCRVFKKIVIEAKPRDQHQQQHQPYVHTSSNISGSSSFDVCSDLEISSNTPYNTAAHIQPRFGNANAISDHDDWSQYLSQNMPTSFSDYGSPYGPYLTQSKVNTEVQCEMFQHQMSLPPLRVENSQAQTSDFSKRLHQNSGQSGFDDFTFAASNSNQFYNSNVDDHLIHIGNLDEQSYIEEQELILPSFQSNDQDLELYGGSRTNTIDNIEIDDFFSFENQAQDNDNSNVTPNSAGFEMIGEEIIVNHKMLISTRQTTEILYYQVVPSQILKIHINPVHGNEERTMLMEEDSDDSWFQKAENVAKMKLKQISLVAKRYYKCLTIIF

>ANAC087

MAVVVEEGVVLNHGGEELVDLPPGFRFHPTDEEIITCYLKEKVLNSRFTAVAMGEADLNKCEPWDLPKRAKMGEKEFYFFCQRDRKYPTGMRTNRATESGYWKATGKDKEIFKGKGCLVGMKKTLVFYRGRAPKGEKTNWVMHEYRLEGKYSYYNLPKSARDEWVVCRVFHKNNPSTTTQPMTRIPVEDFTRMDSLENIDHLLDFSSLPPLIDPSFMSQTEQPNFKPINPPTYDISSPIQPHHFNSYQSIFNHQVFGSASGSTYNNNNEMIKMEQSLVSVSQETCLSSDVNANMTTTTEVSSGPVMKQEMGMMGMVNGSKSYEDLCDLRGDLWDF

>ANAC088

MMPMTEEFYDEEEELGCFRFNPSEEELILDYLLPKLGFHQPNTIYLLEDRDNIYAKEPWRLNHTENDIFEPNEWFYFVKRTNRKVKGWKATGELKDVVSKKTGEVIGKKRNLRFYVEGDESKTSGWTMREYSSIGNQNQRLCHLKGP

>ANAC089

MDTKAVGVSKDTAASMEASTVFPGFKFSPTDVELISYYLKRKMDGLERSVEVIPDLEIYNFEPWDLPDKSIVKSDSEWFFFCARGKKYPHGSQNRRATKMGYWKATGKERDVKSGSEVIGTKRTLVFHIGRAPKGERTDWIMHEYCVKGVSLDDAMVVCRVRRNKEYNSGTSQKAPKPNSSAEKHAKVQNGATSSGSPSDWDNLVDFYLAGESGEKLLAEMAESSENLQVDNDEDFFADILRDEIINLDEAVMTGNTPNEVPTLESASMEIRVLPLPNMIDKQMSSLLEERPSQKKKGKDATESLSSCFVGLYSIKSVNKARWDVIIGVVALIAMLFYLE

>ANAC090

MADEVTIGFRFYPTEEELVSFYLRNQLEGRSDDSMHRVIPVLDVFEVEPSHLPNVAGVRCRGDAEQWFFFVPRQEREARGGRPSRTTGSGYWKATGSPGPVFSKDNKMIGAKKTMVFYTGKAPTGRKTKWKMNEYHAVDETVNASTIPKLRREFSLCRVYITTGSSRAFDRRPEGVLQTERMLTSDVAVAETSFRVESSLETSISGGEHIDVSMNTEFVDGLSEPMWDWEQLTWP

>ANAC091

MKEDMEVLSLASLPVGFRFSPTDEELVRYYLRLKINGHDNDVRVIREIDICKWEPWDLPDFSVVKTTDSEWLFFCPLDRKYPSGSRMNRATVAGYWKATGKDRKIKSGKTKIIGVKRTLVFYTGRAPKGTRTCWIMHEYRATEKDLDGTKSGQNPFVVCKLFKKQDIVNGAAEPEESKSCEVEPAVSSPTVVDEVEMSEVSPVFPKTEETNPCDVAESSLVIPSECRSGYSVPEVTTTGLDDIDWLSFMEFDSPKLFSPLHSQVQSELGSSFNGLQSESSELFKNHNEDYIQTQYGTNDADEYMSKFLDSFLDIPYEPEQIPYEPQNLSSCNKINDESKRGIKIRARRAQAPGCAEQFVMQGDASRRLRLQVNLNSHKSETDSTQLQFIKKEVKDTTTETMTKGCGNFTRSKSRTSFIFKKIAAMGCSYRGLFRVGVVAVVCVMSVCSLVA

> ANAC092

MDYEASRIVEMVEDEEHIDLPPGFRFHPTDEELITHYLKPKVFNTFFSATAIGEVDLNKIEPWDLPWKAKMGEKEWYFFCVRDRKYPTGLRTNRATEAGYWKATGKDKEIFKGKSLVGMKKTLVFYKGRAPKGVKTNWVMHEYRLEGKYCIENLPQTAKNEWVICRVFQKRADGTKVPMSMLDPHINRMEPAGLPSLMDCSQRDSFTGSSSHVTCFSDQETEDKRLVHESKDGFGSLFYSDPLFLQDNYSLMKLLLDGQETQFSGKPFDGRDSSGTEELDCVWNF

>ANAC093

MAKKEKIEQVISMGGIMWEGLNSSLIKVDEALLKQQIREFEKGNDKEWFIITERNKVDQGLSQTKRVGNGAKRQKRVDTNGGYWHATVAAQKINAGDGVVGNKRPLAYYDRKPSEDVKTDWLMQEYSLDHNNDKVRLHFVQDLSYSTSNKEVGEEKKKQKKGEPVEASEGQQPCNAEYHQPLAPLDSCQPQPHDLAEQLDLHQPEQLQLQQPHDIVYQPQYCLLPEQHQLQPFPDNFSELNSFQQQPVMIPDDLEDFLAELMEPHSLDGDEESNNYGFFEGLFDTEGINDKTLH

>ANAC094

MVLVMDDEESNNVERYDDVVLPGFRFHPTDEELVSFYLKRKVLHKSLPFDLIKKVDIYKYDPWDLPKLAAMGEKEWYFYCPRDRKYRNSTRPNRVTGGGFWKATGTDRPIYSLDSTRCIGLKKSLVFYRGRAAKGVKTDWMMHEFRLPSLSDSHHSSYPNYNNKKQHLNNNNNSKELPSNDAWAICRIFKKTNAVSSQRSIPQSWVYPTIPDNNQQSHNNTATLLASSDVLSHISTRQNFIPSPVNEPASFTESAASYFASQMLGVTYNTARNNGTGDALFLRNNGTGDALVLSNNENNYFNNLTGGLTHEVPNVRSMVMEETTGSEMSATSYSTNN

>ANAC095

MGPTYRALPVGMRFRPSDLELAVYFLIKKALGLPMKALTVPDECNDIFSTHPRDLPGYGSEEHWYFYCKKPKNQVTRTKSYNLWIPTGEKTDVLDPKKNGGELVGIKHSFTFIENEEEEESDNKNGDEEEPPQCNWFLDEISLPLTVVDTDWTLCHIFYEKVKPEFGNLHIVESESESEEEEEDESVDKPAESLDSVKEKDGTVLPPPPATP

>ANAC096

MGSSCLPPGFRFHPTDEELIEYYLKRKVEGLEIELEVIPVIDLYSFDPWELPDKSFLPNRDMEWYFFCSRDKKYPNGFRTNRGTKAGYWKATGKDRKITSRSSSIIGYRKTLVFYKGRAPLGDRSNWIMHEYRLCDDDTSQGSQNLKGAFVLCRVAMKNEIKTNTKIRKIPSEQTIGSGESSGLSSRVTSPSRDETMPFHSFANPVSTETDSSNIWISPEFILDSSKDYPQIQDVASQCFQQDFDFPIIGNQNMEFPASTSLDQNMDEFMQNGYWTNYGYDQTGLFGYSDFS

>ANAC097

MDISAQRFAMNGRSMRLPPGFRFDPDDEDLVFEYLAKKVLHRPMDFDLPELRSCNVDPWDLLGEKNKEVYYFVKKEERERKGRETLSGYWEECEEEEVMEAGGRDCIHLEGRRKTFAFFIGKKPRGTITPWIMYEFRLLSSRATRWSSSPLPRGEVGKWRAVKVVVKEENDEEMVEDEHESDESDGEEVIQSR

> ANAC098

MDIPYYHYDHGGDSQYLPPGFRFHPTDEELITHYLLRKVLDGCFSSRAIAEVDLNKCEPWQLPGRAKMGEKEWYFFSLRDRKYPTGLRTNRATEAGYWKATGKDREIFSSKTCALVGMKKTLVFYKGRAPKGEKSNWVMHEYRLEGKFSYHFISRSSKDEWVISRVFQKTTLASTGAVSEGGGGGGATVSVSSGTGPSKKTKVPSTISRNYQEQPSSPSSVSLPPLLDPTTTLGYTDSSCSYDSRSTNTTVTASAITEHVSCFSTVPTTTTALGLDVNSFSRLPPPLGFDFDPFPRFVSR

NVSTQSNFRSFQENFNQFPYFGSSSASTMTSAVNLPSFQGGGGVSGMNYWLPATAEENESKVGVLHAGLDCIWNY

>ANAC099

MKNSKCNLIDSKLEEHHHLCGSKHCPGCGRMIQAATKPNWVGLPAGVKFDPTDQELIEHLEAKVKGKEENKKWSSSHPLIDEFIPTIDGEDGICYTHPQKLPGVTRDGLSKHFFHKPSRAYTTGTRKRRKIIQTDHDSELTGSSETRWHKTGKTRPVMINGQQRGCKKILVLYTNFGKNRRPEKTNWVMHQYHLGINEEEREGELVVSKIFYQTQPRQCVSNTNWSDHHGSKDVIGIGVGDEISSVAATLQSLGSGDVVSRVNMHPHTRSFDEGTAEASKGRENQHVSGTCEEVHDGIITSSMSSHHMIHDHHNQHHQIGDRREFHMSSSYPMTPTITSQHESIFHVTSTMPFQGSVVWFGIRRPNYGLYHSYVYRRRKFLTNLCI

>ANAC100

METFCGFQKEEEQMDLPPGFRFHPTDEELITHYLHKKVLDTSFSAKAIGEVDLNKSEPWELPWMAKMGEKEWYFFCVRDRKYPTGLRTNRATEAGYWKATGKDKEIYRGKSLVGMKKTLVFYRGRAPKGQKTNWVMHEYRLEGKFSAHNLPKTAKNEWVICRVFQKSAGGKKIPISSLIRIGSLGTDFNPSLLPSLTDSSPYNDKTKTEPVYVPCFSNQTDQNQGTTLNCFSSPVLNSIQADIFHRIPLYQTQSLQVSMNLQSPVLTQEHSVLHAMIENNRRQSLKTMSVSQETGVSTDMNTDISSDFEFGKRRFDSQEDPSSSTGPVDLEPFWNY

>ANAC101

MESLAHIPPGYRFHPTDEELVDYYLKNKVAFPGMQVDVIKDVDLYKIEPWDIQELCGRGTGEEREWYFFSHKDKKYPTGTRTNRATGSGFWKATGRDKAIYSKQELVGMRKTLVFYKGRAPNGQKSDWIMHEYRLETDENGPPHEEGWVVCRAFKKKLTTMNYNNPRTMMGSSSGQESNWFTQQMDVGNGNYYHLPDLESPRMFQGSSSSSLSSLHQNDQDPYGVVLSTINATPTTIMQRDDGHVITNDDDHMIMMNTSTGDHHQSGLLVNDDHNDQVMDWQTLDKFVASQLIMSQEEEEVNKDPSDNSSNETFHHLSEEQAATMVSMNASSSSSPCSFYSWAQNTHT

>ANAC102

MDFALFSSISIFEINHKDPPIRRFIKTQNRILSTRKQQGTFPKMKAELNLPAGFRFHPTDEELVKFYLCRRCASEPINVPVIAEIDLYKFNPWELPEMALYGEKEWYFFSHRDRKYPNGSRPNRAAGTGYWKATGADKPIGKPKTLGIKKALVFYAGKAPKGIKTNWIMHEYRLANVDRSASTNKKNNLRLDDWVLCRIYNKKGTMEKYLPAAAEKPTEKMSTSDSRCSSHVISPDVTCSDNWEVESEPKWINLEDALEAFNDDTSMFSSIGLLQNDAFVPQFQYQSSDFVDSFQDPFEQKPFLNWNFAPQG

>ANAC103

MGKTNLAPGFRFHPTDVELVRYYLKRKVMGKKFQVDAIAEVDIYKFEPPDLPDKSCLGTGDLKWYFFCPREKKYPKGGKANRSTECGYWKTTGRDRDVSYNDEVTGKIRTLIYHYGKIPRGDRTDWVIHEYRLEDKVLAQKNVPQDTYVLCVLFKKNGLGPRHGSQYGAPFKEEDWSDKEEEYTQNHLVAGPSKETSLAAKASHSYAPKDGLTGVISESCVSDVPPLTATVLPPLTSDVIAYNPFSSSPLLEVPQVSLDGGELNSMLDLFSVDNDDCLLFDDFDYHNEVRHPDGFVNKEAPVFLGDGNFSGMFDLSNDQVVELQDLIQSPTPHPPSPPAQASIPDDSRSNGQTKDD

>ANAC104

MNLPPGFRFFPTDEELVVHFLHRKASLLPCHPDVIPDLDLYHYDPWDLPGKALGEGRQWYFYSRKTQERVTSNGYWGSMGMDEPIYTSSTHKKVGIKKYLTFYLGDSQTNWIMQEYSLPDSSSSSSRSSKRSSRASSSSHKPDYSKWVICRVYEQNCSEEEDDDGTELSCLDEVFLSLDDLDEVSLP

>ANAC105

MMKVDQDYSCSIPPGFRFHPTDEELVGYYLKKKIASQRIDLDVIREIDLYKIEPWDLQERCRIGYEEQTEWYFFSHRDKKYPTGTRTNRATVAGFWKATGRDKAVYLNSKLIGMRKTLVFYRGRAPNGQKSDWIIHEYYSLESHQNSPPQEEGWVVCRAFKKRTTIPTKRRQLWDPNCLFYDDATLLEPLDKRARHNPDFTATPFKQELLSEASHVQDGDFGSMYLQCIDDDQFSQLPQLESPSLPSEITPHSTTFSENSSRKDDMSSEKRITDWRYLDKFVASQFLMSGED

>PmNAC001

MTNYSGNRLVGFRFHPTDQELISYFLYKKAVAKEPLMASYSNIVHDFNLLGETEPWVVWDIFGGPDLIDEDLYFFSELKNLSPKGSRIKRKIEAGGTWSEAFSKKVYDETTGNPIGRKRNLRYENEGCEHHGKWLLEEYSLVDQETQKIVLCRLKKNNRVWNKINNSSQELKEKPSNKKARKGLGSPRTKIEDEEPHRTSGPSSVYNNLDQDLIIDTNYNAISNNICGDNQDNMLMTSDFASDPVCFDYGGLTDEEYVFDVNELLATVEPQPLSLELPQMAETLEPLLSSGLGDDRAVEAWNSSAIKGQVSIPVNFFDGARGNVNPKSLSIKARLKLP

>PmNAC002

MANSGVTQLLGFRFVPTDPEIIGFFLYKMVVANNPLTLMPPYNKVINKCNLFGNKREPSEIWRDYGGDQLDDQDLYFVSELQRNDLRIQRKTGRCGTWSETETYQDVKDEVDEIIGRKWKFQYENGNTSEDHAGWLLDEYSLFEKGCKNRTSRNCYDFDVVICRLRRKCNTDKSGKKRKCSSQDQSNKKTKRDQPTKEMKTENSVGPQIMNDESNQLMINDITTCDQDYLIDTNHKSFDIDELFVKLDRKPPLLNSQQLPQIVESLLLASVLINLMDHWKQPPMI

>PmNAC003

MANSRNVSLAFHFHPTDQEIIRSILYKMVIEGEPLNPSYHGIVHDEDLFGTKELWKIWEDYGRDQLHDQDLYFLCQLKRINYCSSRTHRRIGREGTWSQRVAPKLIYDGNPNPIGSVRKLRYKNPKSEHDAEWFLDEYSLFVGDDGHDQTTPGFDFVVCRLRRNHNKFDRIRASVHKKTNKAPQQIHERKRKSMNVVINIDDEEEEEVHGCSSAKSHSCTL

>PmNAC004

MANSRHVSLAFRFQPTDQEIIRSILYKMVVEREPLNPSYKGIVHDEDLFGTKEPWKIWEDYGGDQLYDQDLYFLCQLKRLNFSRSRTHRKVGCEGSWRGVVSKLVYDGNANPIGRIRKLRYKNPKSEHHGGWYLDEYSLFVGDDGHDQTTPGINFVICRLRKNHRSGRIRAGVDKKTNKAPQQIHENESKRKSMNVIINIDDEEVPHGCSKNARTRESFRKRGSFFSPEFLSFPTALF

>PmNAC005

MEKVSTFPSSALQLLPPGFKFHPSDEELIVHYLRNKVISHPLPmQLITEIDLYKYNPWELPNNALFGEDEWYFFSPRDRKYPNGVRPNRAAASGYWKAASSDKPILTSRGLKRIGLDDWVLCRVQQRGNRSKNTCNSQDSYSTEFWRYLPKVEQTQATYANLHADIITDFLYKDCQLLASILSGQAPSPTETNSSVSFQGSKDSYEVRSNNMVKSTISISSSVIDSNTLKMKSTEENMKENHFPLINNRESKKIDKNLLPSMRPmGNAINCYIPNHSQYDLLNAHPTDPTNFEEFNEMTFPGTYFE

>PmNAC006

MADSDDRVGFRFRPTDQELITLFLYKMVVEKKALMATYDTIIRKFNIFGTEFEPSEIWNGFGGEQLHPHDQELYFFSELNTLSSKAGSRKINRKVGLGGGTWSGERCDAVYDEEGNEIGHKRKFRYENEGSEEHSGWFLEEYSLSGEGCAFDYVICRLRKNERLFGNKRKCSSSLSEHQYPKKKMLTKPKPSLTTTKGKKVKKDEQEMGSQENMNLEPQNYRSPTTLVVSDDEKGNNQLTYNNHDDQFINSNYFPDDNTMMINDIITLDDINTLDDTKMIFSEMEWPLPDLPQIADQNHVPLPmSSVGADSVDGVIEACEPSNLVNDNQMGNNNSDEHQDQLLNNTDYCPHDTLIFLGDSDEEGYLINSPTIDDDMDELFPDLAGEWPLPQMPQIAATDESQVPFPTSEVGAESTNGEEMEAYCNPSHFQNMGNYQPLEEDINCFSNYSFVDELMGH

>PmNAC007

MANNNRLLGFRFRPTDQELISYFLYKKSITKDPLMASYYSNIVHEFNFLGETEPWVVWDQFGGPGLVDEDLYFFTELKNLSQKGKRIKRKIEAGGTWRETFSKKVYNKTTGNPIGQKRNLRYENQGCEDHGKWLLEEYSLLDKEAQKIVLCRLKKNGRVSNKRNNSTQEFKEKPSNKKARKELASATVKIDDEQPQRSSSLSSVVNNHDQELIFDINYNAISNYLCGDDQDNIDDPFDYGCLTDEEYAIDMDALLGAPPLSQELPHIDETLRVEAMNSSGLENICNQQLDDITYWFNE

>PmNAC008

MANSGVTQLLGFRFRPTDQEIIGSFLYKMVVEKRPLTSMPQYNKVIHKCNLFGNNREPSEIWRDYGGDQLKDQDLYFISELQRNGLRIQRKTGRGTWSETETYQNVDVIGRKRKFRYENGNASEDHAVWLLDEYSLFRKASKNGTSSNCYDFDVVICRLRRKGNMDKSGKKRKCSSQDQSNKKMKRDQSTKEMKTENSVGPQIMNDERNQLMINESTTYDQEYLIDTNYKSFDIDQLFTELDREPPLLHSQQLPQIAESQEEPCVDQINGPmEAATSNLSNFEYNSDELAGLIGQYNMNADDYEDPNLFIDVDELLAADAEPLSTFAELDDVHNVVSQPLDQNGYWSNSFMDQTYNSFMEEIMAD

>PmNAC009

MANNNRLLGFRFRPTDQELLSYFLYKKAITKDPLMASYYSSIVREFNFLGETEPWVVWDQFGGPGLVDEDLYFFSELKTLSKKGKRIKRKIEAGGTWSEAFSKNIYDKTTGNPIGQKRNLRYENQGCEHHGKWLLEEYSLLNKEAQKIVLCRLKKNSRVSNKRNNSTQEFKEKPSNKKARKELASATVKIDDEQPQRSSSLSSVVNNHDQELIFDINYNAISNYLCGDDQDNIDDPFDYGCLTDEEYAIDMDALLGAPPLSQELPHIDETLRVEAMNSSGLENICNQQLDDITYWFNN

>PmNAC010

MANSGVTQLLGFRFRPTDQEIIGSFLYKMVVEKRPLTSMPQYNKVIHKCNLFGNKREPSEIWRDYGGDQLTDQDLYFVSELQRNGLRIQRKTGRGTWSETETYQNVDVIGRKRKFRYENGNASEDHAVWLLDEYSLFKKACKNGTSSNCYDFDVVICRLRRKGNMDKSGKKRKCSCQDQSNKKMKRDQSTKEMKTEISVGPQIMNDERNQLMINESTTYDQEYLIDTNYKSFNIDQLFAGLDGEPPLLHSQQLPQIAESQEEPCVDQIDGPmEAATSNLSNFEYNRDELAGLIGQYNMNADDYEDQNLFIDVDELLAADAEPLSTFAELDDVHNVVSQPLDQNGYWSNSFMDQTYNSFMEEIMAD

>PmNAC011

MEELPPGYRFYPTEEELVSFYLLNKLEGKRDDTRRVIPVVDIYRKEPWDLPKFSGELCHGDTEQWFFFTPRQQREAQGGRPNRTTASGYWKATGSPGYVYSSDNKVIGMKKTMVFYKGKAPTGRKTKWKMNEYRAIEADNPTTTTTTNSVPKYLRNEFRLCRVYVVSGSCRAFDRRPLEGGGETKHQLSESMGGTSTFSLMAPMVEKASSSDETYSYSGELADLPETSAGINSTDWEIIEGLEQPLWEWEQLNLL

>PmNAC012

MEELPPGYRFYPTEEELVSFYLLNKLEGKRDDTRRVIPVVDIYSKEPWDLPKFSGELCHGDTEQWFFCTLPmSIPTSYSNIICFPV

>PmNAC013

MAEDGYAYDRVPPGYHFYPSEEELLLYYLRPKVNGEEVPGENHVVFDFNLYSDQPRKIWDHFQTTRQNDLKMSNDLYFFTELQTKTTNGSRVSRTAGGGTWKGEDGGKKIYAPGTDHVIGIKKRFRYENKGSAEHGRWLMHEFELGQSLIHNRQAKKYVLCLLRKKEEPEKKRKEPEEEDRRQVLCL

>PmNAC014

MVMPGFRFHPTEEELVEFYLRRKVEGKRFNVELITFLDLYRYDPWELPmMAAIGEKEWFFYVPRDRKYRNGDRPNRVTTSGYWKATGADRMIRSENFRSIGLKKTLVFYSGKAPKGIRTSWIMNEYRLPHHETERYQKGEISLCRVYKRAGVEDHPSLPRSLPmSKASTSRSSVLVAPSTTISSDNKKQHNTSTNINNNNINTVHSIMEKLQAFNEGQSQQFHQDPEHQVDQKMSVEPEGSSGNSDVTTVLGLSKRNAYHHNNIGQAHHHPLDQEEGMAAFLHHSNSKQAAANCFSLIPSSTNSSATLFTAGSSSVSSSNMNAIDDLHRLLTYQHVQQQAAAASSHHQQQHVHHHVVQYYDDAHHHPQPNNLFSTFPmAAPHVQSAIPPQQLAPNALPTAFSDRLWDWNPISDPNRPDYNNPFK

>PmNAC015

MAPCEYVDVPIGFKFRPSDDELLRYYLLNKVSGTPFKYSNVVPEFNLYGKKEPWDIWNDFGGQNLEKGEDLYFFTKLKLVTGKGSRVARTIGNGTWKGEDKGTTVRDPmKKNTPLGLCKRFRYENEKSDQHGCWIMHEYSLHPSLVKPQSNSNTCGCDGKYVLCRIRKNDRKRKLSENDGVGGHALILQSPTKILRQLESSDEVVLGANSTPISKASEVTNHYNQQPEQTTTANAYLPMDQPHYFLTDYPTTMASASTSPPPSLPQPDYGFELISDTDHQPmQGAGFNYDLSQFLDSDDVDALLKDLSNLDNIQPLTAQPLGGGSHPYPCTIEESAVDEDSGNINTGLGISSEDQTGGELGTENVSLNVSGFPVHDDEMLDFLNRASFDDLVDMIQFEDNDDELQAFAKSESVMGMESTTTATTY

>PmNAC016

MGDDQFKLPPGFRFDPTDEELVVHFLQRKAALLPFHPDVIPDLHLYPYDPWELDGKALREGNKWYFYSRRTQNRVTSNGYWKALGVEEPVITRSSCNSTHKVGMKRYLAFYVGEAPNSGIKTNWIMHEYRLSPLSAGDGPSSSSTTTRSSSKRRGHLKTEFSGYVLCRVYERDEDDDDDDGTELSCLDEVFLSLDDLDEISLPN

>PmNAC017

MSSSDLQLPPGFRFHPTDEELVKHYLCRKCQSQPISVPIIAEIDLYKYNPWDLPGLALYGEKEWYFFSPRDRKYPNGSRPNRAAGSGYWKATGADKPIGSPKPVGIKKALVFYAGKAPRGEKTNWIMHEYRLADVDRSPRKKSSSLRLDDWVLCRIYNKKGTVEKQQQQQQQQQQQTTSRMSSGSEFEDRKPENLACPPPPmVVAPTRPNDYVYFDTSDSVPRLHTDSSCSEHVVSPEFTCEVQSEPKWKEWEKALDFNYNYMDTSVENGFGPQFQSANQMSPLQDIFMYLQKPY

>PmNAC018

MTGEQKEELPPGFRFHPTDEELITFYLINKITDATFTGRAIADVDLNKCEPWELPmKAKMGEKEWYFFSLRDRKYPTGVRTNRATNTGYWKTTGKDKEIFNSVTSEMVGMKKTLVFYRGRAPRGEKSNWVMHEYRIHSKSAFRTSKDEWVVCRVFQKSAGIKKYPTNQSRAAHPYNLEIGPSVVPSPMMQLGDHQFPYGRNYMTSAELAEISRVLRTGGSGGGGSSNGTINVPIQPQFNYPNLGGGGGGGFTISGLNLNLGAPmTQPMLRPMPPPQLHHQGPPmMNHHQQDVMSNGANNNSLGPDHQAGYGAIDMNNNNANGNGNRFMNMEHCVDLDTYWPPY

>PmNAC019

MGDPKATSLRSLPPGCRFYPSEEQLLCCYLSNKNDADPmQSGGYDLIRELDLYDHDPFDLPDSACYAYGHAGRKRHWFCYTVRVLKERRAKSGYWRRKGRDRDVVGRGGKAVLGRRTSFVFYLGNSPNTAVRTDWVLYQYAQVDHVKASFVLCRVFVRSRRGNRLSYNWVSSCAEESACTVRHIGIQHDGFHTPNIVETEVHGDNSAHRKNEMLSFPMRLDCEINDRVKNGPVSISRLQPNGQVPSSVLGGSNPIFLDDLATEHLLSIVEGDFIELDDLND

>PmNAC020

MRPQSAALPmDIGFQCTDEELCISLGKIISGSPLPGNVIEDANPYQLVPSNLPDGFWYFIHSNENKPTNFGYWRTKGEACRIFSNSSITGWRATLEFYQGHAPHESKTNWVMQEYWLTQKKLSEDSKAKEPRSLCRVFCSNEQKSNCKNLQKMVSSDSASYSTHSAVPRAKNCTSHSSTSKPQVIKDNERGTLIGTERGPDHNVQIMPEIDYVSRGDYLELLDLDTPmSFSFSSDSSCLTMSSDECFDSLALLEELEPKNSQDLVNKNAGCKFSISAPPRSDELVMFAASSGSFSKSPSEERVKTHSPIPGSAVCGKISGKTSNNMSRIQKPDNRNEGGPSTSHNIDVSPSSNTASQEGKRRARTIGRTEKLKKKYFCFMPF

>PmNAC021

MALPSPQSTSQVPETEKDFDLLKCSKIENGKKEAEIHLVGYRFDPSEDQIVVFYLFNKILGRELPINDIIKEISVYDHDPDELPNGDFKHGLDCNEAFYFAYTEQVYSSRGKITRRTTKSGYWDLDGEEEEVKYRNGDITVGFEKVMVFHKGTAPNGIETDFIMHEYRVNPLIVPTHVLNDSIRAKIERYVVCRIIHEGVSNFPRTNFHQDGLLGLLKKQNQIDMTEIA

>PmNAC022

MALPSPQSSSQVPETEKDFDLLKCSKIENGKKEADVDLPTGYRFDPSEDEIVVYYLFNKILDRELPITDVIKEISVYDHDPDELPNGDFKHGCDCNEAFYFTYTEQIYSSEGKITRRTTKSGYWDLDGEEEEVKYGNGDITVGFEKVMVFHKGTAPNGIETDFIMHEFRLNPLIVPTHVLNDSTRAKIERYVVCRIIHEGISNYQPTFKLPPGWTARPVDEGEPS

>PmNAC023

MEAKHSSELPPGFRFHPTDEELIMFYLKNQATSRPCPVSIIPEVDLYKFDPWQLPEKAEFGENEWYFFTPRDRKYPNGVRPNRATVSGYWKATGTDKAIYSGAKYVGVKKALVFYQGKPPKGVKSDWIMHEYRLSDSRKQANKQLGSMRLDDWVLCRIYKKRHLGKAYLDQKVEEEDPITDPKIEITAANNHEEQMMLKFPRTCSITSLLEMDYLAPISQLLSENSSYDFQNNLASAGNAGHAQMFQFGEMPYQSTTDTAKFQVTQSCPSNQPWFANPVHNLNGLDRN

>PmNAC024

MDERSDMNIIGGDKMDEVMLPGFRFHPTDEELVGFYLKRKIQQRPLSIELIKQLDIYKFDPWDLPKLAASGEKEWYFYCPRDRKYRNSTRPNRVTGAGFWKATGTDRPIYSSEGACSSKCIGLKKSLVFYKGRAAKGVKTDWMMHEFRLPSLTDSLPPKRSSSFMDKTIPmNDSWAICRIFKKTNSSHAHQRALSHHSWVSVLPETNTYDDHMLGSKAAHSATHHHHQHEFSSERMPLTSKTNSTTNFCGNNNNDIQISSTTSSMSPLDIYTNSSYKPVNPLMMGCNIKLPYQYFPISNSNGDYFSSTPSFTFSSSPLETPSGPVAKCTVDVSSLLLNMSSSMLGDYNGSNSINKASAESTNFEFSTNTGSQDQCNGGFSSMTLPHHHEVMQGNNNMGNGHDTVVGLIKNNISNVNSDVTEDADDQRWETSVRSNTGVPLMMSNSFPLPLNIGGGGGGDAWKSSLTWESSPCPSEMSTTRCYT

>PmNAC025

MERLNFVKNGVLRLPPGFRFHPTDEELVLQYLKRKVFSCPLPmSIIPEVEVCKADPWDLPGDCEQERYFFSTREAKYPNGSRSNRATGSGYWKATGLDKQIVTSRGNQVVGMKKTLVFYRGKPPHGTRTDWIMHEYRLVLAENTAPNAPQEKNSTQLQSPVVPMDNWVLCRIFLKKRGGKNEEEQVQQPCNDRVIRKTKTARPVFYDFMTKDRRNLSLAPCSSSSGSSGVTEVVSSDQLDDHEESSSCNSFPYCRRKQ

>PmNAC026

MEESLVPFGFRFRPSDEEIVGSFLYPFLVESKPFMSLYNNFFRACNLFGNNTEPSEIWKKYGGPQLVDTDLYFISKLKKLTPKRMDRRIGNGGTWSETESSKLVHEKVSGNPNPNPIGRKRKFRYENKGSEDHTGWLLDEYSLFDGPKNDYNQRGYDFDFVICRMRKNDRVGIKATNLKRGSQDKEENKMTTNKKMKTDDQMGSTESSSQQGCSSSPVGGDLVGFDPIDLTIFEGNTMADMEQLLGEAWSPSNFEDAVSYDVDPIGETQINFENEENTMADMEQLLGEDWSPSNFENEENTTANMEQLLGEAWSPSNFENVVSHDVDPIGETQSSQLANWSQATLDQLLVGV

>PmNAC027

MEKQSISSKASIVTKNGSDLHGGHTGSYDSYYDDASYFDSIPPGYRFKPLDGELVAIYLRKKIANEPLPPNKIHDVELYKFNPDTLSEIYESYGEDEMYFFTSRDKKYPNGARPNRAAGDGYWKATGVKKKVKFEDVEVGSKTSLVFYRGKPPHGDKTDWKMHEFQVNAPPKRKDRNNMRLDDCVLSRIYNKHNHSKDSNQSNNNGVEIQEQPNVGSNSQDNAHVSGEVVDHPPmNSHELTSLLSNQNCYYNTDQSEILNSTNCMGFDGPFGVVSISSTLSFQNPQQFNGYQKGQRFCDLPSEFYTNYDQWLNDTAKDLTH

>PmNAC028

MANSGVTQLLGFRFHPTDQEIIGSFLYKMVVEKRPLTLMPPYNKVIHKCNLFGNKREPSEIWRDYGGDQLKDQDLYFISELQRNGLRIQRKTGRGTWSETETYQNVKDEVDEINGKSNLDVIGKKRKFRYENGNTWGDHADWLLDEYSLFKKACKNGTSSNCYDFDVVICRLRRKDNLDKSGKKRKCSSQDQSNKKMKRDQSTKEMKTENSVGPQITNDERNQLMINDSTTYVPEYLIDTNYKSFDIDELFAELDCEPPLLHSQQLPEIVESQEEPCVNQFDGPVEAATSNLSNFEYNIDELAGLIDHEDPNLFFEVDELLAEDAEPLSTSELDDVYNMVSQPLDHNSYRSNSFMDQNYKCNWL

>PmNAC029

MESTDSSSGSQVHPQLPPGFRFHPTDEELVVHYLKKKAASAPLPVTIIAEVDLYKFDPWELPSKATFGEQEWYFFSPRDRKYPNGARPNRAATSGYWKATGTDKPILTSIGNQKVGVKKALVFYGGKPPKGIKSNWIMHEYRLVNNNNNNNNFSKPPDASNKKASLRLDDWVLCRIYKKNNAQRPMMDEDSSNNSMDAKAHYKLHGVARKR

>PmNAC030

MTWCNDSDDERAIQIITPPPPPPPSHNNNEFRNITCPSCGHHIEFQDQAGIHDLPGLPmGVKFDPTDQEILQHLEAKVLSDTRKLHPLIDEFIPTLEGENGICYTHPEKLPGVNKDGQIRHFFHRPSKAYTTGTRKRRKVHTEEDGSETRWHKTGKTRPVLVGGAVKGFKKILVLYTNYGRQRKPEKTNWVMHQYHLGNNEEEKDGELVVSKVFYQTQPRQCGSSIKDVPMFDHHKGVNIRSGHGHDPLPIAKKAGLVEYYNPGGGFINYDHHNLHNQGGHNRESPPQLIPNLVVQGDGSSFIRLTADASKGKLERK

>PmNAC031

MDQKTPGGRNYTEMEVQNMMSDSNNIKEDANLLPGYRFHPTDEELVRFYLRRKVENKPIRLELIKLIDIYKYDPWDLPKASGIVGGKEWYFFCRRGKKYRNSVRPNRVTKSGFWKATGIDKPVYSVGDFHSCIGLKKSLVYYRGSAGKGTKTDWMMHEFRLPmSSSKDDTSNIKNYTQEAEVWTLCRILKRDNNYSCRKYASNWQKTTSNPKQIVADSSSKPCSPESDISFGALAANKDQFERSPILYNGHMYEGNRFSAGDQLSLKGEVDPMVNSYISFSNPKEDEMLTSDGNWDELRPMVDCVLNPSLLYSCR

>PmNAC032

MASEQYYMSLHPEVRFQPKDEELITYFLKRKISGDPLPINIIIDELSFYEYSPIQLSQMYAFANQKEMYFFTPVERKFTDSTLPRRKARNGYWIPTSKYENIEQGTEIIGVKKSLRFYAGRHRNASTKTNWTMLEYIAGGTAAVSQSPYFLYDWVLCKVYEEELSAKKVPESSNQGNAADEEVTGEQLHSHIRTSPLEDLFGAKKRRCLSKNSGQLPLTICTSSYRRPCLPELPFEVIIEILSRLPVESVLRFKCVCRQWCSTFREEDFIAKHWVRASPLRLPYRYIWDYNNSVSFYEENFKLIGNSCGLFLEKNYSSQVFRIRNFAMHQVLYLPHAPNAYINTMCFVLNLSTGECKVAYFYIKGNLDAEVGLKILTVGIDCQWRPLKLSNQTIWGQHEKYLLKRHILKPNQIEGAAHYVEIIRAGQDLCLEVQSFDLLTECFVTTRLPEWVFGNLEKVFVFSWNHHLAVGEIVEEALNILVLEDFKDHKWSENKIVVPYKFLKDNPSLKNQIWAPVRVIYGKLELQGVKSFLTYDMEREIITRIEAGNDPDKEHFASHKPSLLTFKGMRPKRVRCDY

>PmNAC033

MASGNGQLTVPPGFRFHPTDEELLYYYLRKKVSYEAIDLDVIREVDLNKLEPWDLKEKCRIGSGPQNEWYFFSHKDKKYPTGTRTNRATTAGFWKATGRDKAIHLANSKRIGMRKTLVFYTGRAPHGQKTDWIMHEYRLEDDNISADQVQVKLSIIDVK

>PmNAC034

MAPMSLPPGFRFHPTDEELVAYYLDRKINGRTIELEIIPEVDLYKCEPWDLPDKSFLPSKDMEWYFYSPRDRKYPNGSRTNRATRAGYWKATGKDRAVNSQQRPVGMKKTLVYYRGRAPHGIRTNWVMHEYRLVDSVCGNASSSLKDSYALCRVFKKTIQMPKNNKEEKPIGIDNAENDSVWVSNNEQLLGEDNSGINETASRGIETDQDENYSNHDYPKFLSDTSSSDLTQGTPTENGIADDFQAPFASDEANSSADLYSFGVRCSSNLIDQETYIPHNESLLNNYQFPYPPLELEDFPQINLAAEANASKPEIIDDYMSYDKFKDYMNGTFEEIFSLCSSQDNSAALPMQEL

>PmNAC035

MDSNYTSTKDDHQYIDEDDVQLPGFRFHPTDEELVEFYLRRKVEKKAISIELIKSIDIYKHDPWDLPKAISTSGEKEWYFFCKRGRKYKNSIRPNRVTGSGFWKATGIDKPIHSHGGQGHACIGLKKTLVYYRGSAGKGTKTDWMMHEFRLPNPmGSNEYIHRSSNTKNINSTDPQQEAEIWTLCRIFKRNVSYKKADWRGQSGAKRHPMDTVSSKKCTSDQDDLDQSNNYISFGASDNIICYEEKKPVVNHTNMNNFNGSNQQLHAVGQLSSNMPmTYPYMETFSNFSYQDLENELLNENWDELRSAAVHLAFDPTFLV

>PmNAC036

MGGYQVPVGYRFTPSEEELLLHYLLPRVNGNDYPKGVVPDCDLYGTKEPWDIWRDFHHSSADDQEDIYVFTTLKKKTPNGSRFCRTVGAAGTGGWKGEDSGKKIRACGNDIGIRKRFRYMNPGSPHDDRWIMLEFQLHESLVQVPNKETIVLCLVRKKETSGKSKLEERKNHEVDMASRGEIQAQHVHDDQQQHIDASTYDQLRFLDDYLMNDVGGEQNTMVNPTLLAQYDQQDMIGTSTEDQLRVFEDFLMNDVEGEQDTMVNPTLLAQYDQQDMIGTSTEDQLRVLEDFLMNDVEGEEDTVVNPmLLAQYGQQGTSIEDQLRLFEDYLMSDIEG

>PmNAC037

MGGYQVPVGYRFRPSEEELLLHYLFPRVNGNDYPKGVVLDCDLYGTKEPWTIWRDFHHSSPDDQEDIYVFTTLKKKTPNGSRFCRTVGTAGTGVWKGEDSGKIIRARGSDIGIRKRFRYMNPGSPHDDRWIMLEFQLDESLVRVRNKETIVLCLVRKKETSGKSKLEERKNHEVDMPSRGEIQAQHVHDDQQQHIDASTHDQLRVLDLMNDIEGEQDTMVNPTLLAQYGQQGTSTEDQLRFLEDYLMNDIEG

>PmNAC038

MGGYYQVPVGYKFRPSEEELFLHYLLPRLNGEDYPEGVVPDCDLYGTKEPWEIWRDFHHSSPDDQEDIYVFTTLKKKTPNGSRFCRTVGAAGTGVWKGEDSGKKIRACGSNIGIRKRFRYMNPGSPHDDRWIMHEFQLDESLLRVRNKETIVLCLVRKKETPGKRKFEELQNHEDVPPRGEIQAQHVHDDQQHHIGPSTYDELRVLDLMNYVEGEDNMVNPTLLAQYDQQEHLGTSTDDQLIRFLEDYLADVEQN

>PmNAC039

MGGYQVPVGYRFTPSEEELLLHYLLPRVNGNDYPKGVVPDCDLYGTKEPWEIWRDFHHSSPDDQEDIYVFTTLKKKTPNGSRFCRTVGAAGTGVWKGEDSGKKIRACGNDIGIRKRFRYMNPGSPHDDRWIMLEFQLDESLVRVRNKETIVLCLVRKKETSGKSKLEERQTHEVDMPSRGEIQAQHVHDDQQQHIDASTYDQLRVLEDFHMNDVEGEQDTMVNPTLLAQYNQQYMIGTSSEDQLRVLEDFLMNDVEGEEDTVVNPVLLAQYGQQGTSIEDQLKFLEDYLMSDIEG

>PmNAC040

MVKAIEEELKRRAHKGKSGAVPSAKCRVPSSSSLSLSRVLMFHPRDEELVCDYLLKKVTQTDSTLMIEVDLNKCEPWEIPVNACVGGKEWYFYSQRDRKYATGLRTNRATAAGYWKATGKDRPILRKGSLVGMRKTLVFYQGRAPKGRKSDWVMHEFRLEGPLGPPKISSHKEDWVLCRVFCKNREIAAKPSMGISRYDDTSNSSLPmLMDSYISFDGQPQTQPQPQPHHEYEQVPCFSIFSQSQTSPIFSHIDITGAHLEPNMPTKNNNNNPmTFGALPNVTSFLDPFSCDKKVLKAVLSHLTKMEETSSYPTLNVKGSSPPSLGAGSSSENYLSEVGMPNIWSHY

>PmNAC041

MDIMSFTGHFDHGSDTHLPPGFRFHPTDEELITYYLLKKVLDSSFTGRAIAEVDLNKCEPWELPDKAKMGEKEWYFFSLRDRKYPTGLRTNRATEAGYWKATGKDREIYSSKTCALVGMKKTLVFYRGRAPKGEKSNWVMHEYRLEGKFAYHYLSRSSKDEWVISRVFQKSSGSTTNGPGSKKTRMSNGSTSSISLYPEPSSPSSVSLPPLLDSSPYQNMNTAGLTDRDSCSYDSPIPKEHVSCFSTNPNSGFNLSSSCFDLAHAQPPPQPGFGGVSAFPSLRSLQENLQLPFFFSPMSGHQPVHVGSSGGSTIDLGGLSSAGNWPPPPPHQEEPRTVGPTELDCMWTYYKTECL

>PmNAC042

MGVPETDPLSQLSLPPGFRFYPTDEELLVQYLCRKVAGYQFSLQIIAEIDLYKFDPWVLPSKAIFGEKEWYFFSPRDRKYPNGSRPNRVAGSGYWKATGTDKIITTEGRKVGIKKALVFYIGKAPKGTKTNWIMHEYRLIEPSRKNGSSKLDEWVLCRIYKKSSSSAAQKPMTTSVSSIEHSNGSSSSCSSQLDDVLEWLPEIDDRSFTLPRINSLKTLQQQQEDSKLGFQTGSGNFDWASLAGFNVVPELCPNNQPQQGQGQMNVNYSNNNDMYVPSIPPLCHVESPPERLAKTVDEEVQSGFRTQRVDNSGFFQNSNVMTQNFCNPTDPYGYGTRLGRSALGFGCGEK

>PmNAC043

MESTDSSTGSQQQQQQQQQPQPPPQPNLPPGFRFHPTDEELVVHYLKKKVTSAPLPVAIIAEVELYKFDPWELPmKATFGEQEWYFFSPRDRKYPNGARPNRAATSGYWKATGTDKPVLTSGGTQKVGVKKALVFYGGKPPKGIKTNWIMHEYRLADNKTSNKPPGCDLGNKKNSLRLDDWVLCRIYKKNNSHRPMDLEREDSMEDMMGPLMLPSISHVGHHQNMNLHLPKSDTNYGPPFIENDPIFFDGIMSSTNGSASLSNGTSQLPLKRSIVPSFYWNDQDDNQTAGASSSKRVVQLHQLDSGTNNSASDGINGAPNNNSTSIANLLSQLPQTPPLHQHAMLGSLGDGLFRTPYQLPGMNWFSESNLG

>PmNAC044

MGPCAVDLPVGFYFHPKDSDLLGYYLHNKVSGKPMKYEVPKINLYGTSEPWKIWKDFGGDGLEIGEDLYFFTTLKTKGTRVSRKAGNGCWHGENSAKVLDPKNEQRVLGFSRRFHYKNPKSDQNGCWIMHEYSLKDYPSMPKSKSSSVSNDDGDQLVLCRIRKNDSKLHKNERKRKFKSAAEDHGIDDAPVQSKSKIQKINIDDHDPIGLTSYEPEFVTNPIGLTQSNHQPEMIDDNQLLGDDFDEALNIAFSYLDGPTIIENNTTPQMQSYSSMLLCAEREEMGNTPCTDHETERHTSQSFATGSEFIEVTDDIGFGTSKETSHEYLDIDDQIAQINMSEPDYLQLDDHVFEMRLFDDDDPQ

>PmNAC045

MAPCASVVNLPVGFKFRPRDDQLLGYYLLNKVRGTSFMYENVIPEMDLYGKIEPWDIWYEYGGHNLAKGEDLYFFTKLKSLSDKDSRVARTIGSGTWKGENSGTTVSDPKNEENDLGIWKRFHYENPKSVQDGCWIMHEYSLHPSLVKPKPNSTNQFVLCRIRKNDRGKRKLRTAEEDNETDTPVQSQNKRQRPQQVTRFEELIGNCTPMSEATGVGGSVSYLPTELTQSQPDSSFAYPTTVVSSQARANYTDDVSQFHGGGDGNALMSDFSHLDTAQSFTEQALGSHAVCNQERASDAYETQQGLGLTDNNIGHWPSPFGSEEDQVNALDFSIDYELLIHLIDCDDGPPQSSTAQFMGMENTTTASSEANMVIID

>PmNAC046

MAPCESAVNLPVGFKFRPRDDQLLGYYLLNKVRGTTFMYDNVIPEMDLYGKIEPWDIWHEYGGHNLAKGEDLYFFTKLKSLSDKDSRAARTIGSGTWKGENSGTTVSDPENKENDLGIWKRFHYENPKSVQDGCWIMHEYSLHPSLVKPKPNSTNQFVLCRIRKNDRGKRKLRTAEEDNETDTPVQSQNKRQRPQQVTSFEELIGDCTPMSEATGVAGSVSYLPTGLTQSQPDSSFAYPITVVSSQARANYTDDVSQFHGGGDGDALMSDFSHSDTAQPFTEQALGSYAVCNQERASDVHETRQGLVLTDNNIGYWPSPFGSEEDQVNAFDFSIDYDLLNHLIDCDDDNDGPQKSSTAQFMGMGMENTTTASSEANMAIID

>PmNAC047

MEPVEQSHPHNKASVTSQVASYSLINKQSHAQAHLTHELTQPNEPGNSLITNVSAGAELSDDESNSFNNNLKNEEDEFFDSFPPGYRFNPLDEELVVHYLKKKVLDQPLPPNRIIEVNLYRHNPEFLAEKYRKYGEAEWYFFTPRDRKYKNGSRPKRAAGNGYWKATGADKAVRSNGALVGFRKALVFYRGKPPKGDKTNWIMHEFRVKDSPVRRKRGENDMRLDNWVLCRIYKKVDKNSKRCETRNHVEDPLSPQPQIINGEGMEMETEMSTLFDPMVGYDNKYSIMQNVYGVSHNGMIHSQLQAVPMNEVPSYPNIGHPQAFGARASSMPLLPDSLLESRYIDESTIHPDDLYNLKAFERSLYDTYQRQFPPVENFYPYLE

>PmNAC048

MGSGSELELPPGFRFHPTDEELVNYYLCRKCAGQPLAVPIIKEIDLYKFDPWQLPELALYGEKEWYFFSPRDRKYPNGSRPNRAAGTGYWKATGADKHIGKPKALGIKKALVFYAGKAPRGVKTNWIMHEYRLANVDRSASKKNNNLRLDDWVLCRIYNKKGSIEKHNVAMERSKMTKYPEILHEQKPEMTQMPPPHTDMSSMDSAPRVQQTTDYSSCSEHVLSPEVTWEKEVQSELQWSSDELENSLNTLDNQFINYMDGFSDILDPFGAAQPQYQMDQQNMFAYLQTQF

>PmNAC049

MAPVSLPPGFRFHPTDEELVAYYLKRKINGRKIELEVIPEVDLYKCEPWDLPGKSLLPSKDLEWYFFSPRDRKYPNGSRTNRATKAGYWKATGKDRKVNSQTRAVGMKKTLVYYRGRAPHGIRTDWVMHEYRLDERECETAQGLQVQDAYALCRIFKKSATGPKIGEHYGSTSTTNYQLTSDHSSSVELYSDGGRCEDFESSSYPMQMNACSSSPNFVHTSSLDMARKRDGKWTQFLSKDAFNCSSSFSSFPNHGNVPYPPSKVDIALECARLQHRLSLPPLEVEDFPRVGLNDFKTMQYSNPmVEPTSTETDALQEILSVAHVSQEMINQSSNLADQTWGGNYAAPmNDFSFIVDRDAHYNQITDHMNSMRYVEKSWENPYTRSIEIGDLEDDFRMENTAENLRWVGISDKYLEKSFMEENKVVPLENISSFQREEQEHEVQGEIGDQSGMIKEFNDSETNDDFSLGFINEDPNDNFLNDGTMDDYSPSPSFEVIEEIQVNHGMFVSTRQVAETFFHQLMPSQTVKVHLNPmLGQNFFVERVDTQTKCENIGSFFESFKAFGMDKFVGVSKSTKPWRKVASTLVCAVTLLLMHIIYFGQHVEDEKLMDAFTSTTTAAIVEEKGNSNVSKKKKGLGLIKWSNKEEKVCLVNIRGGNSCSVLLKKIGVFLTVSLALCTMWAL

>PmNAC050

MAPCEYVDVPIGFKFHPSDDELLRYYLLNKVCGTPFMYSNVVPEFNLYGKKEPWDIWNDFGEQKLEKGEDLYFFTKLKLVTGKGSRVARTIGNGTWKGEDRGTMVGDPmKKNMPLGLCKRFRYENDKSDQHGCWIMHEYSLHPSLVKPHSNSNTCGGDGKYVLCRIRKNDRKRKLSENDGVGGHALVLQSPTKILRQLENSEEVVLGANFTPISEASEVNYHYNQQPEQTSSAYLPMNQPQDYGFELISDTDHQPmQGFNYDLSQSLDDDDVDALMKDLSNLDNIQPLAAQPLGGGSRPYPSIIQESAVDEDSGNFNSGFGISSEDQTGGELGTENVQLNVLGFPVHDNEMLDFLNRASFDDLVDMIQFDDNDDELQAFATSQSVMGMESTTTAATC

>PmNAC051

MSLYNNIFHARNLFGNKTEPSEIWKKYGEPQLVDTDLYFISKLKKLTPKRMDRRVGNGGTWSETESSKLVEKVSGNPNPNPFGRKRKFRYENKGSEDHTGWLLGEYSLFDGPKNQRSYDFDFVFCRMRKNDRVGIKATKFKRGGHKIKKKRR

>PmNAC052

METTRSLGLLVGCRFEPYEAELLAHYLYNKVNGLPLSSNAVIECDLYGEEEIWRKLFDETGETSLYFFTELKKKTGKGSRVERMSSGGCVTWRNQSNKVICVNDNEPNKIVIGYKRMFSYVPKKTSTIAKGEWVMHEFWLAGCLVNNNNQPSLIN

>PmNAC053

MEVAKVMRNSSDQKVKDDDEEMMLPGFRFHPTDEELVGFYLKKKVEKKPISMELIKQIDIYKYDPWDLPKVSTVGEKECYFFCRRGRKYRNSIRPNRVTGSGFWKATGIDKPIYSVKEPLECIGLKKSLVYYRGSAGKGTKTDWMMNEFRLPPHGHGKTTNFLNAKDITQEAEVWTLCRIFKRIPSYKKYTPDNWKEGNTKQNPTDSSSKTCSFESENYCGEPHVSPQDSVVMQRIERSSSKPVRVDEQVDETNQWFTFVGETPYATSYSSLRDIPTDNDFFINGNWDELRPVVQLAADPSFPSL

>PmNAC054

MGSGNGVYSVGDFNLDAKWLIDPKHLFVGPRIGEGAHAKVYEGKYKNQTVAVKIVHRGETPEEIAKRESRFAREVAMLSKVQHKNLVKFIGACKEPVMVIVTELLLGGTLRKYLFSMRPRCLDICVAVGFALDIARAMECLHCHGIIHRDLKPENLILTADHKTVKLADFGLAREESLTEMMTAETGTYRWMAPELYSTVTLRHGEKKHYNHKVDAYSFAIVLWELIHNKLPFEGMSNLQAAYAAAFKNVRPSAENLPEDLALIVTSCWKEDPNDRPNFTQIIQMLLHYLSTISAPEPmIPQRIFRSENVVLPPESPGTSSLMTTHDDSGETPKIDVEEKPKVYPFRKKAPLRKASATKDSTPEPEARSPSLLPRAHRLRSWVFPLAHCLLHFNLVRAWLINSRGLANKVKNAARSSAYQIKDCGANRDCPNCHFVIDNSDVLSEWPGLPAGVKFDPSDAELLEHLAAKCGVGNSKPHMFIDEFIPTLDGDNGIYYDHPmNLPGSKNDGSSFHFFHKTINAYATGHRKRRKVDCHRSLAAEHVRWHKTGKTKPIMENGVQKGCKKIMVLYKSSKKGSKADKSNWVMHQYHLGTEDGEKEGEYVVSKIFYQQHKQSETNDNNLVIEDDVNALGTSPRTPITSAPNPPRPGKSVGCDDVPDEDVLHSSAKEAELVPGTQFEDNIGDTTWLAGESQADENCDLDCLEDTLLCKETFNSSTLLNGSFADRITDFPSNIYGVTGNSSTSCGIGDLENIDLGTPPDFQLADLQFGSQESLLGWLDRNVFLLRDVSAKLLSNNVVQPSKPSEDCFFSSSTPSNPFDGWCRSMSTSRGSSSRGSSMRSKVAKRMQKESGKTLREVRRAKKLQKKLMTENERLIYNLKRAKRKVALLLQKLKKYELPDLPmPRHDPELLTLEQLQAFKKIGFRNKNYVPVGVRGVFGGVVQNMHLHWKFHETVQVCCDNFPKEKIKEMATMLARLSGGVVINIHNVKTIIMFRGRNYRQPKNLIPFNTLTKRKALFKARFEQALESQKLNIKKIEQELRRSGVNPEDPVAIASIQRVASSFFNAIDQKDGSPYVFRGDKLSSAEPDNKLKHSDPPDEEEEEDLDKFIAEIEDAAEREWAAEEAAEKEEQTRLRYWNKEQFGGRYRRSEDVENDDSDDETTRARGWRDTHGKQRPNDSDYEDDEEESDSSNVVDASVLNSDADDSDGKPEKFKVSTRDRGKQDKFGRAKNDERFKKNREANNTKASSRNIIVDKGTESESMLSDLDTVMWKSDSEEEHDSTVSRAVNYDFRSSSDEEEDSSYRRGERKKLANDLDKAHDKFEVSRNAQGKHHRIGKADNNDYLKRNGDVNVRRKLVEEDVVSENTFGASESAIWELEAVEDVGAPTAGRYNYKSSDEEDYQVRRVGKKVKDTNKSTRTTKELDEDWDSD

>PmNAC055

MDEKNDMDKIDDVMLPGFRFHPTDEELVGFYLRRKIQQRLLPIELIRQVDIYKYDPWDLPKLASTGEKEWYFYCPRDRKYRNSARPNRVTGAGFWKATGTDRPIYSSEGSKCIGLKKSLVFYRGRAAKGIKTDWMMHEFRLPSLSESAAPPKKLLDKSLPmNDAWAICRIFKKTNSMAQRALSHSWGSPFPEPTAASGLLNQGAHCTQFSSENMSCTTDIGSSIQFWSNNDLQQASNASYSAFDTLPYKPINPTGSKPSIFDADQFPNGFMFSPGEMSGPISPmLIGDLSNATGSIDFDGSQQQQFSGFSINLSQNQMQGNMETGENEGGFRKNLSSSTSHANNNNNNGQWGSIRPIAFPFSLASDDDAWKPTLPSDSPPCPSDMSTSYSTNKCYS

>PmNAC056

MENAYGFSKEDEQMELPPGFRFHPTDEELISHYLSPKVLDSCFCARAIGEVDLNKCEPWDLPWKAKMGEREWYFFCVRDRKYPTGLRTNRATDAGYWKATGKDKEIYKAKTLVGMKKTLVFYKGRAPKGEKTNWVMHEYRLEGKYSAYNLPKTAKNEWVICRIFQKSSGGKKTHISGLVRLGSFGNELRPSLLPPLMDSSPYNSDTRTTVCETSHVSFSDPMEDQRTQDDIIDSFQHNNHHNSSNSNHNPLLASSSRSNPSAHLNPFYSNQITPNIGFLQHQDSLLMQDQSFLRMLLDNQAPNLRRSAKTELSQDTGLSMDVSSVVSNREMVQDDPSYSSAPIEYDSLWNY

>PmNAC057

MEKLPNFVINGGVKMPVGYRFHPTDEELVIHYLKRKVHAAPLPmSIIPDFDVFHTHPWGLPGCGGDVREKRYFFYNKINANEIDDTKRAAGCGYWKPMGKEKQIVDPESSEAVGIRKTLVFSQRKRRYHETQTKTRWLMHEYQLLSSQVNPTHTQASKREPGNWVVYRVFQRKRKPQRSSDIIPQPSNSKRTRTQRLVEVITPSSSSSCSSDITHLSSNANRLDHDQEENSSAAADN

>PmNAC058

MGREAEQPKAIVLAATAPVTPPPmPTALAPGFRFHPTDEELVIYYLKRKVCRKPFKFNAISEVDIYKSEPWDLADKSSLKSRDQEYYFFSALDRKYGNGARMNRATNLGYWKATGNDRAVKHNDITVGMKKTLVFHSGRAPDGKRTNWVMHEYRLVDEVFEKAGLGSIQDAFVLCRVFHKSNIGPPNGHRYAPFVEEEWDDDDKLTLVPGQESRNEAVAGRDAFIIGNGHAACNDQNAHAARSEQNAHAARSEQSGHAARREQNGHAACIEGNDHAACNGENGHGTSVQGNGHGTSVQGNGRGTSVQENGHGTSVQGNGHGTSIKGNGHATSFEGNDHGASVKGNGHGTSVKENGHGTSVKENGHFCQRENGHGTSVKGNGHLTSVEGNGHGIIIGDDHGTTNEGNCLEMVEYGPDMHNAGNNFEQDTQPISNALVTDGELPTENENDLPLCKTERMDDYPMTCVVNREERLDDYPSPGPDDAQPLLSLFNRHPGQLRQYKRRRHNDSNSNHSNASDISSGMTHDPCSSTTTTASTEASMTTTTRNFLSALVEYQLLESLEPKDTTPPPPPEFSAALLHSSVPPSCLKYVETLQNEIHKISIERETLKFEMMSAQAMINILQARIDLLNKENEDLKRNNV

>PmNAC059

MGRNSLAPGFRFHPTDEELVWYYLKRKVSGKNFRFDPISVIDIYKTEPWDLPGKSKLKTGDLEWYFFSFLDKKYGNSSRTNRATEKGYWKTTGKDRPVLHNSRNVGMKKTLVFHSGRAPKGARTNWVMHEYRLSNEELEKSGIQQKDPYVLCRIFQKSGTGPKNGEQYGAPIAEEEWDDDDVTCVPSELAADNVVVVSEGPYVETNDVSNGAYVEAFDNDQKFDAGIPSECPPLNFYYGETSNYVEHSGEFVDDGTTAVMGTGETSEYHEDQKFFDLPVDYEMGGKTVKDEYLTSENYDDLKFFDVPEHSEADAKLVKDECFIEPSNDTNPmDVNYPLNEPYFNTAENPPVGDGLFLETNDLSNPVESTAGFDMLDEYLTYFDADDDISQYIDFDSCGMMGVENSVPDQSPVDQKLVNGETEQLPMGVEHLAQAEDTNDASSSEQKPEFKLESDVNYPFIKKASHMMLGNIPmPPmFASEFPmKDAILRLNSGSASSSPVHITAGMIRIRDITSSDNRMDWSFGKDGVVNLVFSVQLSQNDGNSGNLVPMDGSLSGKTGCVVMRGWFLFMFFWVLFLSMSFKIGSYVYTR

>PmNAC060

MAPENMSISVNGQSQVPPGFRFHPTEEELLQYYLKKKVSNQSIDLDVIRDVDLNKLEPWDIQEKCKIGTTPQNDWYFFSHKDKKYPTGTRTNRATAAGFWKATGRDKVISSNCRRIGMRKTLVFYKGRAPHGQKSDWIMHEYRLDDNNTSNCITNVPMIVGEAAQEEGWVVCRIFKKKNLHKIGSGSTIMSTTTSSMTTETRSAQSLFDSCPEGALEQMLQYIGRTCKEEENEGNNMSTRFNLQPINTGVNNHNGYHERFLKLPSLDSPNSTSSQDCYQPNIHEEVMAAENNIDEDFQHQMDSGLTNWAALDRFVASQLNGQAETSRQLACFSDPYDCNDDHHELQLPTTLRSISSSSSNKSYHATQDYNNGEINLWSFARQSSSLSSSDALCHVSNGPI

>PmNAC061

MDCGFRFCPTEEELVNHYLRKKKQDKDFKVDHIIPEIDICKYEPWDLPGLFTEPESPYQDMFFFSPRDYKYINNRARTNRVTGRGFWKITGKERVIKGARGSIGRKKTLIFYEGRVTQCNRTNWVMHEYYLCEDEAIPNPKLAQQRDFVLCRLRKNAVKNVTSICAVAEPmYAEWLGISEEECPQPEELELVFPmLQLQDNISEEMEFEIFSKK

>PmNAC062

MDSNYRFLPTEEELVSYLREKKQVDHIIPGINICNHEPWEVPmLYEGLFTEPDSPYQGMFFFSPRDYKYSDNNYARTNSATARGFWKTTGKERVIKAGGSIVRKRTFFYEGRVPPYNKNNWVMHEYCLIEDEANPTPKLAQQRDFVLCCLKKKQDKKDTSIFAQPDEWPLISEEYPQPEEPE

>PmNAC063

MDSYFRFLPSEEELVSHYLREKKQVDHIIPEIDLCKHEPWDVPGLYAGLFTEPESPYQDMFFFSPRVYKYNNSARIERTTALGFWKITGKERVIKARGSTGKKKTLTFYEGRVRQSKKTNWVMYEYYLVEDEANPNPKLAQQVMQRDFVLCRLKKKADDHTSIYAEAEPmYDK

>PmNAC064

MNTFSHVPPGFRFHPTDEELVDYYLRKKVSSRRIDLDVIKDVDLYKIEPWDLQELCRIGTEEQNEWYFFSHKDKKYPTGTRTNRATAAGFWKATGRDKAIYSKHDLIGMRKTLVFYKGRAPNGQKSDWIMHEYRLETDENGTPQEEGWVVCRVFKKKIASLRIKMNEHESPCWYDDQVSFMPDLDSPNQNSNSNINMAPYHHLPYPCKKELDHLPmFQFPHEHFFQLPFLGSPKLLQSSATGVSSSNSMAAHAYSIDINHACTFQPSDQDQNFHGAVYGNNSNDDHPQAVDQLTDWRMLDKFVASQLSQDDASNKGNSYS

>PmNAC065

MKSCVKQFHLSEPLHAFLYIIRTEEQEAKLDNMNTFSHVPPGFRFHPTDEELVDYYLRKKVSSRRIDLDVIKDVDLYKIEPWDLQELCRIGTEEQNEWYFFSHKDKKYPTGTRTNRATAAGFWKATGRDKAIYSKHDLIGMRKTLVFYKGRAPNGQKSDWIMHEYRLETDENGTPQEEGWVVCRVFKKKIASMGIKMSEHESPCWYDDQVSFMPDLDSPNQNSNSNINMAPYHHLPYPCKKELDHLPmFQVPHEHLFQLPLLGSPKLLQSSATGVSSSNSMAAHAYSIDINHACTFQPSDQDPNFHGTVYGNNSNDDHSQAVDQLTDWRMLDKFVASQLSQDDASNKGNSYS

>PmNAC066

MLVVDMTVLSTNTLPLGFRFRPTDEELIDYYLRSKINGNHKQVTVIREIDVCKWEPWDLPDLSVIRTTDPEWFFFCPQDRKYPNGHRLNRATGRGYWKATGKDRQIKSATILIGMKKTLVFHTGRAPKGKRTNWVMHEYRTTQKELDGTNPGQNPFVLCRLFKKQDETIEDSNLAEVEPTVSSPTAAISSPQDTQSDHALGPmSPSFERNEKPTKVECTTADCSNGMEPDTLQPIECPSNCYNGYDAEDQLNVPLEELDIFYDPPQQPLFSPLHSQMQTELYYCGSNNFNNGHQGVKFQYGTNDQDADISEFLSSIFNSSGEHSDVDAKVAVPCETEFQNPVQSEGNIDRKGPVQNESTRIDCDQSSMFRVTSNHSNVFLSGDAGNGIRLRARKTQNLTSTENFARQGDAPRRLRLQRKVLYCSNLSKDWNCRPEDRETKPMAVEEIRDTEEKAVDAAVSDAVDAATDPPKKETMSTRPNFTPEGSILTGERVPNVILQASPmGHSMWSFAFLFRVVVVVAFLFIMLLAYGDFISFDAAGSPFTARMA

>PmNAC067

MGLRDIGATLPPGFRFYPSDEELVCHYLLKKITNEEALKGTLVEIDLHICEPWQLPEVAKLNANEWYFFSFRDRKYATGFRTNRATTSGYWKATGKDRMVMEPGTGEIVGMRKTLVFYRNRAPNGIKTGWIMHEFRLETPHMPPKEDWVLCRVFHKGKGEENTKLSPHDFMLETTSCTVPLSMEKYSSPPTQDQTMPRGYNPIPSFSTPPPRHSHNQSNSLLNLLHFPQEKYSNNSVPELGAKNDDEYGFLWDMSLEETSFENGVAPDMDEMRFEMDHNSMVLL

>PmNAC068

MAANTKDQEQMIPEFELPGFRFHPTEEELLEFYLKSVVFGKRQRFDIIGFLNIYHHDPWDLPGLSKIGEREWYFFVPRDRKHGSGGRPNRTTETGFWKATGSDRKIVSLSDPKRIIGLRKTLVFYKGRAPRGTKTDWVMNEYRLPDNCHYLKDIVLCKIYRKATSLKVLEQRAAMEEEIKNFHASPNTSSPPTSMETISFCSQPQEDLTLQMPKNNVVLKQEMEDSSLTEAKEPKEEKAMEIKGSPPPPTYILPELQVPRLSMDWTQDTFWTQMNSPWLQNLTPLPNLLNF

>PmNAC069

MAHYQNVPLGFRFHPTDKEIVGSFLHTLLVDRNPLMPPYSNFIRACNLFGNKLEPSEIWNKYGGPQLVDQDLYFLSGLKKLSPKRMDRSIGHGGTWSETESFKLIEYDNGNPNPIGRKRKFRYENKGSEEHTWWLLDEYSLFVGPKNDYNDRCYDFDFVICRMRKNDRASSKEINLKRSSQDQVQKKRSTNKKMKKDHQMGSTEESSSHVQQGCSSSLTGGELVVSYDDVDPTDLTFFEDNPIFNIEQILCETKVEDACSPSNSENVDSSYSKWTESYMEELMACI

>PmNAC070

MAHYQNVPLGFRFHPTDKEIVGSFLHTLLVDRNPLMPPYSNFIRACNLFGNKLEPSEIWNKYGGPQLVDQDLYFLSGLKKLSPKRMDRSIGHGGTWSETESFKLIEYDNGNPNPIGRKRKFRYENKGSEEHTWWLLDEYSLFVGPKNDYNDRSYDFDFVICRMRKNDRASSKEINLKRSSQDQVQKKRSTNKKMKKDHQMGSTEESSSHVQQGYSSSLTGGELVVSCDDVDQTDLTFFEDNPIFNIEQILCETKVEDACSPSNSENADSSYSKWTESYMEELMACI

>PmNAC071

MSSISVSVNGQCQVPPGFRFQPTEEELLLYYLRKRVSCEKIDLDVIRDIDLTKLEPWDIQAEKCKIGSLPQNDWYLFSHKDKKYPSGTRTNRATIAGFWKATGRDKVIYSNGRPIGLRKTLVFYNGRAPHGERSDWLMYEYNHIVSNAMGGAATEEEGWVVCHIFIKKKKNLKTLNSPSNSSFNYMGINTGHMLDSYYDEGSLEQMFQQVERNCKEENKAEVVKYDTKPLIYTSNQVIGAPDPKSLHQIVSGLTSWAAFDRLIASQLNGHTA

>PmNAC072

MESCVPPGFRFHPTEEELVGYYLKRKINSLQIDLDVIIDIDLYKIEPWDIQARCKLGYSEQNEWYFFSHKDKKYPTGTRTNRATAAGFWKATGRDKAVLSKNNIIGMRKTLVFYKGRAPNGNKTDWIMHEYRLQTSEHAPPQAKGWVVCRAFKKPSPSHRQGFEALKHAYYIREHNSHASSSNFGSEMHMVNPSLGFQYQSFGLDQQDHLISNHIFMGNQLTELPQLESPSISTSLANKQGVHEQQNNNITTEHYDDQRSNMNSSDQHMDWRNLDSLLEVPLIPQNYEQEAQNHHLLGCFPDL

>PmNAC073

MGSEDDPEMLFRNGRFCAPGFRFHPTDEELVVYYLKRKICKKRLKLNVIAETDVYKWDPEELPGLSLLKTGDRQWFFFSPRDRKYPNGGRSNRATRHGYWKATGKDRNITCYSRSVGLKKTLVYYKGRAPSGERTDWVMHEYTLDEEELKRCRNVQEYYALYKVYKKSGPGPKNGEQYGAPFREEEWADDELPVINNPmDRQIPVKQSVDVISVDPVKVNGEVHSALSDIEEFMKQIADEAVLELPQMNGYAYTIPQAVSEEETQSTVVDLYSREVVCPEPNTVFNPSHHQGNLQASFDFTQSDTSQIQRYEASEVTTSAPEIHEQGPPILREEDFLEMDDLLGPEPTISNIENPVDNLQFEGIDGLSEFDLYHDAAMFFHDMEPFDQGTVSHQQYMNSLGNNIVDQFEYQLQPNPPmVNQVNHQLNPESTQMNNQLWTHTERAEPNQGCLSYSTSGVVYEPSNFASQANQNQSGNEAAGGPSQFSSALWAFVESIPTTPASASENALVNRAFERMSSFSRLRINSVSANVTAGSSSEARRAGRKRGFFFLPVVVALCAIFWVLMATLRPWWRCLSA

>PmNAC074

MGKSSLAPGFRFKPTDVELVQYYLKRRLLGKRLGFKVIAEVDIYKYDPWDLPDKSCWDCGDLKWYFFCPREKKYRSGNRVQRATEGGYWKTTGKDRSVLYSGAIVGWIKTLIFHTGRAPSGDRTNWVMHEYRLEDQGLADRGVPLDSYVLCMIFQKEGLGPRIGAQYGAPFKEEDWSDDEVEITSEAVQVAITPEPDLGLPCNYVSPTATNAHSVGDIGVGPSGSGIYDILPPFCEVSQPVSSNYVILEMPLASVGDDIISMFDCFTEESALYTDEPKKVEAEGRLETVQNANMPSTHSPEVIGVGPSFESCGFDVLPPCNVNESVSCNFVTMENPPmSNGVETLPIMDCFAEESMLFVNDNGKNEELNNLEHLGNATPHFNTSNIYEDLGDLENLGRVGEVGYNFCSEPDSRSHFLELYDLDQPQHPNLFF

>PmNAC075

MRKSSLAPGFRFKPTDVELVQYYLKRKLLGKRLGFKVIAEVDIYKYDPWGLPDKSCWESGDLKWYFFCPREKKYRNGNRIQRATEGGYWKTTGKNRSVVYSGEVVGWIRTLIFHTGRAPRGDRTNWVMHEYRLEDQGLADRGVPLDSYVICMIFQKDGLGPKIGAQYGAPFKEEDWTDDEAEICSEAIPHENMSEPNLVVPSNCNSSITTSRHSLKGIHTSPYESCISDVLPPSCNVHQLVSSNHVTMEKLRGSNDDLLSMLNCFTEGSTSLMKENDKNEELGNVIASGNASATPNFNSDDIYEDLGDLGKMARVSEDGYNFSNVHNSICAPmQMQLGDNEQFLELDDLSW

>PmNAC076

MGKPPLPPGFRFSPTDVELVRYYLKRKVMGKRLHFNFIAEVDIHKYAPWDLPEKSGWQSGDLKWYFFCPTARKYPTGVRVQRGTECGYWKSTGKDRSVLYNSEVSGWKKILIFHKGRSPKGERTDWVMHEYRLEAKDLADSGVPHDSYVICMIFQKDGWGPKNGAQYGAPFKEEDWTDDEAEICSEAVPHENMSELNLVVQSNCNSSVTTSGHSPKGIHIGPSESCISDVLPPSCNVLQLVSSNHVTIEKLHGSDGDILSMLNCFTEGSTSLMKVNDKNEELGNVIHSGNASATPNVNSDDIYEDLGDLGKMAKVSEDGYNFSNVHNSICAPmQMQLGDNEQFLELDDLSW

>PmNAC077

MANSSTGGVPPGFRFHPTDEELLHYYLKKKVSFQKFDMEVIREVDLNKMEPWELQERCRIGSTPQNEWYFFSHKDRKYPTGSRTNRATNAGFWKATGRDKCIRNTFKKIGMRKTLVFYRGRAPHGQKTDWIMHEYRLEDGEDPQGNLSSEDGWVICRVFKKKNLFKVGNEGGSSSMNSSDRQQLNNTSSTNHARSFMHTHRDNEYLLRQQHSQAFELNNPSLHYAHLQPPPQYSLFQPQALISTHKPIAGYDYTPLPSDDSPGHGMVKQLMTNPRDCESGSESLRYQACEPGLEVGTCEPNQAPMVAGGGGRDHHDQGGMNEWAMLDRLVTSHLGNDQDSSSKGARYDQDANAAASSVTQINNHLSLRGEMDFWGYGK

>PmNAC078

MLGMEDAVMSELSGEDINEQGLPPGFRFHPTDEELITFYLASKVYNGSFCGVDIAEVDLNRCEPWELPDVAKMGEREWYFFSLRDRKYPTGLRTNRATGAGYWKATGKDRQVYSASTGALLGMKKTLVFYKGRAPRGHKTKWVMHEYRLHGRLSSYGGHACKDEWVICRINHKTEDKKNPLQLDQVEVEAASSNCNCLPPLLESPTAAAKPTFLQQGQRPHQSHNPMQMQSPHPLPPFLFQHQENDLKSLINPVVSQPHLFSSFPINAFQTQPSPSFSPTTTNSNTALLTNDKNPSPLQSLLFKSLFSSHDQDCNNTIPKQCKTEPNYFSHFQTPmNNNNNCDLNLLNLMEKNHHHHHHHHQPNTPYHQYNNHPNDPLLFDCLDYSVLGFSDAAGTATTVHEHDTCPSTAFNRAGFQTMLDLPPIKVTGESWPLDYIMDAK

>PmNAC079

MGGASLPPGFRFHPTDEELVGYYLKRKVEGLEFELEVIPVIDLYKFDPWELPEKSFLPRCDMEWFFFCARDRKYPNGSRTNRATKAGYWKATGKDRKVVCQSTVTGYRKTLVFYRGRAPLGDRTDWIMHEYRLSDDLAQGPSGHQGVFALCRVIKKNENAQKTNDSHGEPKAKRVGSASNSGEDLSSTRISNEPLNISDDLSSQASYQHNESHYSSHVTSPYEVNPIAEFEPTSRETNPmDFWVSPDFILDSSKDYPQLQEAMPNYFPQYKSAMASWQSYENTESPYSSYSNFTGDVTMADNANQVADMSPFSGHTGYMGYYGNEEMQFEGFDQPSSLRNPTPF

>PmNAC080

MNTFSHVPPGFRFHPTDEELVDYYLRKKIASKRIDLDVIKDVDLYKIEPWDLQELCKIGTDDQNEWYFFSHKDKKYPTGTRTNRATKAGFWKATGRDKAIYSRNILIGMRKTLVFYKGRAPNGQKSDWIMHEYRLETSENGTPQAKGESKQGSTKLCPPQLYMFLCLVFKKRLATVRKIGDYESPCWYDQVSFMPELESPRRSISHPYASPYNQQQQHYSQCKQELDLQYNMPHHHHHDSFLHLPQLESPKVPQSAPYGSMLQSSTLTQEEQLQQHINQQQNMFNSSSSSLVLYNNNDQQSVDQVTDWRVLDKFVASQLSQDQQDASKEVTNYSNAASMFHVANMLANESSRRPDNENLGQDYASTSTSSCQIDLWK

>PmNAC081

MAEDHMNLSINGQSQVPPGFRFHPTEEELLHYYLRKKVAFERIDLDVIREVDLNKLEPWDIQEKCKIGSTPQNDWYFFSHKDKKYPTGTRTNRATAAGFWKATGRDKIIYSGFRRIGLRKTLVFYKGRAPHGQKSDWIMHEYRLDESNTHDTTVCNSMAESMAEDGWVVCRVFKKKNYQKALESPKASFSMESSSNQMHSSRNDGVLDQILMYMGRTCKLENHDQSLNNLSERFMHLPRLESPTLPTFDQDRSFKACYQAIDEMLIDTPSTNQPSNGCDNNDPVEDHDEYPKTRLNDWATLDRLVASQLGQLNGQDQETSKHNLSCFGDPNMAFCSSPPHDHDHDHDVQLSYPYLRTSRSHHQSEVYSNENDLWNFTKSPSSPSSSDPLCHLSV

>PmNAC082

MDKFNFVRNGMIRLPPGFRFQPTDEELVFQYLRCKVFSCPLPmSIIPEVNVCMYDPWDLPGDLEQERYFFSNKESKYRNGSRANRVTSSGYWKATGTDKKIVSSRRNHIVGKKKTLVFYRGKAPNGCRTDWVMHEYCLVDAETTTSINTAENALNQKENWVLCRIFSKKRSCKTDDEKGIRVNNAEMVHAPQTNNNQSPVSSSSSCSSSSGITEVTSSSGAGDEEEISGCTKF

>PmNAC083

MENVPmGMAEQDEQIDLPPGFRFHPTDEELISHYLHKKVIYISFSCKAIGEVDLNKSEPWDLPGKAKMGEKEWYFFCVRDRKYPTGLRTNRATEAGYWKATGKDKEIYKGKSLVGMKKTLVFYRGRAPKGEKSNWVMHEYRLEGKFSVHNLPKTAKNEWVICRVFEKNSGGKKTHISGFVRYGAVGNEIGPSGLPPLMDSSPYSSKTKLASESSYVPCFSNPDPTDVQRNQGIVDYVNNPLFGVSSNSSDFFPRAPFSNSFYSGQSAPVAANFPFPGSVLMQDQSILRALLENNGSNMRQSFKTEGEMVSVSQETGLTSEMNTEISSVMSNLEMGRRPFGDQEAPmAAAGAVDLDNFWNY

>PmNAC084

MEAEHGQGAMMSSGQLPVGFRFMPTDKELVTHYLMNKVFDRPVPmAEAIQDIDATQFYSTHPKNLVTFSCGEREWFFFIHEDDENCSASAQGRKNIRVVGNGVGFWKPNGSENPIHNEDGNVYASKIFLTYFSGSLRKAKKTHWKMVEYHLHSDSHTEEEYQVLEAVQRREWVLGRLKRGNAYNGL

>PmNAC085

MQDSLPPGFRFHPTDEELITYYLCCKVSDVSFTSKAVAVVDLNKCKASMGEKEWYFFNLRDRKYPTGLRTNRATEAGYWKTTGKDKEIHRAGVLVGMKKTLVFYKGRAPRGEKSNWVMHEYRLENKHPFKSSKEEWVVCRVFQKSTSLKKPQQTTTSSQQQQQTSIESPCDETNSIVNEFGDIELPNLNSNNIANSSSGLISTMPSSQSCYNNNINDHSSDVNMSLNMNWRNLIEQNQQQARQILIQNPLLTKALFQAQIMLGMVRPPQVIPSIQPSASQHSQQSTQPTQQSNIQSASVSPGQVGLQDQTGPSQIQAPPRKQYQNQSAMPSSSAAAPSINLQSQPMPSHPLQTPQQPKGHLSHQMTPTSLPQSSQLPNIPSHPLHSSSQPPSLHQTQIPTASGQLQQSLQTSGVLHMPMQPPLPPQPRPPSMPNFHHQYPPQIGPNMGYQHANSQHLPQSMFHSGTKPPmSAGPSFPQGQPPLPSQPPPQSLYQGGGMHLGSEFNNQAGSSMQVDRGSWMSGPPESSSSGPPQLVPGQMGPGSQSTRPPPLTPDMEKALLQQVMSLTPEQINLLPPEQRNQVLQLQQILRQ

>PmNAC086

MVVNRGAEDQLIELPPGFRFHPTDEEIITSYLTEKVIDSSFVAIAIGEADLNKCEPWDLPKKAKMGEKEWYFFCQRDRKYPTGMRTNRATESGYWKATGKDKEIFNNKGKAGCLVGMKKTLVFYGGRAPKGEKTNWVMHEYRLEGKISQYTNLSKAAKDEWVVCRVFHKNMGLKKTNNTPTRMNSFGDHDLLDYSSLPPLMDPTSFNNNIMNNNDNKACGYGDQDVKPGTNLASTAPSLSSAKASSSDDHHAINNNNYLSYFSIGRSGGQVQKPNYSFQVQPTNSYYQATSLSNPNMLYPHHHHQFPNPNPNPSSVWFQPNPNNSDYFQQGMIRVTNNITTSDHDEDDQAMLRAIAAANNNSNMNNETSSSAAAGFGSSGGLERQCKVEQFSSAPQSMFSLSQDTGISTDINTTEISSSLVSSKQELGSAANNCSSYDQRPSVVPISDIEGLWDF

>PmNAC087

MMKNPESSLPPGFRFHPTDEELILHYLTKKVASTPLPVSIIAEVDIYKFDPWELPmKAAFGEKEWYFFSPRDRKYPNGARPNRAAASGYWKATGTDKTIVASLGGRQNVGVKKALVFYKGRPPKGIKTNWIMHEYRLPENPNNYTTTSKTMKLKDLSMRLDDWVLCRIYKKSNVSSSAAVPPIDHEEEVEEEEDFLHDVLLPSLKSPLPGLGHNMSTNNTDLMPQKSSSFSNLLDAMDYSLLTSFLADGQYSNPTGTGLQSTPNRFPCSGTTGLDQKPLFINDYSNISSSSSSSKSHLLQKLPQFNGLNLPmIPSTESRLKRHDHPIADDHGLLYPSKKYVNSNCSFNHATIQSDNISQGHLLNHSLLNHQQLVLSSPQFQFQG

>PmNAC088

MEENNRFINDPIPDQVDMLINDPTNDAYFNSLPCGYRFAPTDVELVSSYLEGKVLNKEIPKNRFLDLDIYLYHPRDLTGRITLMRESEWYFFTSRKRKYPRGQRPDRSAVDGFWKATGKAQDIEDSNGKVIGSKRTLDFYQGNHQDSKRTEWKMHEYTLDTETAPRNEISNTRVTQLDNCVLCKIYNNSKGGNNDSSTIQSDQRAEPSTNVEASQAFPDQQIQPVHQHDQYLVGQSSGASSSSTANKRPRGMPTPTILNTYVGNPTYYPHQQYPFQNQTSGTPPGPVYNNYQNLTQYDQNDQNLTQYDMQSANIESHVQPRESFSSQPGFWLPMETSTYADPMPLVDSSSMQLPVHIRLYEQLLAARNRDGDFAKRLERWYHHQQQLFRQNPSSTPNCKLV

>PmNAC089

MEESNIQVAHEIFYEAWLTDKYINDLPSGYQFVPTDEELIEEYLKKKIKNELLPINRFREVDVYKYHPEELTGMHNLLRESEWYFFSSRDRKYRNGSRPNRAADNHGYWKATGTDEKIKVNNEVIGQKRTLDFYAGKHGEGVKTEWKMHEYVLSENIAPSNDHKANGDMKLNDCVLCKIYKNKRVKKNKNKEIADSVQSAHQPDHQEYPSPLIANSNSSSSSINHHYTAAGMSSTTVNTEFGEPPYYNESFQNEAWSNYPWPITSFNSDQTLDSADTSHGLPMNTFSSPFSTDLEPTNDTALAHPSTSSQFPHDHAKEDDNNSFDPmAQFLNI

>PmNAC090

MRKAPFPPGFRFSPTDVELVQYYLKRKVMGKRLHYNFIAEVDIHKYAPWDLPGKSCWQGDLKWYFFCPTSRNYPTGARVKRANECGYWKATGKDRSVLYNGEVSGWIKTLIFHTGRAPKGERTDWVMHEYRLEAKNLADSDVLQDSYVICMIFQKDGPGPRNGAEYGAPFKEEDWTDDEAEICSEAVPHANMPEPNLVVPSNYNSSNILEGIHIGPSSESCISDALPPSCNVLQLVSINHVTMEKPHVSNDDILSMLNCFTEGSTSLMKENDKTEELGNVIPSGNASATPHVNSDDIYENLGDLGKLATVSEDGYNFSAGHNSICAPmQMLLGDNEQFLELDDLGRSVELP

>PmNAC091

MMESIESTVPPGFRFHPTDEELVGYYLRKKVASQKIDLDVIRDIDLYRIEPWDLQERCRIGYEEQNEWYFFSHKDKKYPTGTRTNRATMAGFWKATGRDKSVYDKTKLIGMRKTLVFYKGRAPNGQKTDWIMHEYRLESEENGPPQAKGWVVCRAFKKRTSSQNKGIEGWDSSYFYEELNGVSSVVDPTEYMISRQPPSFLAQNFMCKQETEADSLNFMHSDHFVQLPQLESPSLPLISKRPSSMSLISENNIEVEDEPKKVTDWRALDKFVASQLSQEERYEGDGESSFGAHDDSEMALLLLQSSGRDDDQGNKLNGFLNSSPDGDIGICIFEK

>PmNAC092

MNKSNLGSIRSSDLIDAKLEEHQLCGSKQCPGCGHKLEGKPDWLGLPmGVKFDPTDQELIEHLEAKVEGKDTKSHPLIDEFIPTIEGEDGICYTHPEKLPGVTRDGLSRHFFHRPSKAYTTGTRKRRKIQTECDLQGGETRWHKTGKTRPVMVNGKQKGCKKILVLYTNFGKNRKPEKTNWVMHQYHLGQHEEEKEGELVVSKIFYQTQPRQCNWSDRASATTGEGSSDIVPmNSRRDSGSGSCSSKEIIPPHSHSHSHREHDQMSAVASGVAAAAPISSYSAMDIHQLKSDHFSFAPFRKSFDEVGIGGEASTAREGPmSGTCEEMRGEHHHQRSVPQPHHHHHHHHMAHEVVPDHPQHDQHQQQQQQLHHQQIATAFHISRPSHPISTIISPPPLHHTSIILDQDSYSRIMLQNENFQAQQQHQQQQQQQHHKMGARSASGLEELIMGCTSSSNVKEESSMPNPQEAEWMKYSSFWPDPDNPDHHG

>PmNAC093

MDRVIPVLYIYEYNPWDLPQFSGEACHGDPEQWFFFIPRQESEARGGRPRRLTTTGYWKATGSPSFVYSSNCNRAIGLKRTMVFYNGRAPHGRKTEWKMNEYKAVEAHHADQNQPLIASSSSNTPSAPTLRQEFSLCRVYKKSKCLRAFDRRPLGVEIMSNPSLNLNQTAAAHEGAGHQGQGAGDQGQGSASHSRNPLMGSDQRTNSSSPESSSSGDHHGPQPPSQPmGETGTLPMFIDNEALWDLEQLMINNNWL

>PmNAC094

MAPREFVFNLPVGYTFRPSDDQLLEHYLLKKNSGEPLQYENVIPEMDLYGKIEPWDIWHECGGHKLAKGEDLYFFTKLKSLGDKDTRVARTIGSGTWKGENSGTTVSDPNNKEKDLGIWKRFHYENPKSVQDGCWIMHEYSLHPSLVKTKSNSTNQFVLCRIRKNDRGKGKLTTAEEDNKTDTPVQSQNKRQRPQQVTSFEELIGDCTPMSKATGVGGSVSYLPTELTQSQPDRSFACPTTVVSSQARENYTETQQALGLYAVCNQERASDIYETQQGLGLIDNDIGYWPSPFGSEEDQVNALDFSIDYDLLNHLINCDDGPPQSSTAQFMGMGMENTSTAISEANMVIID

>PmNAC095

MGIPPGWRFCPTDEELIVFYLRNRTWNNSRIIHLDIYDFTPMQLAEYFEIPEDPVMFFYTTRKRKYKNGKRPSRTANIGYWKATGKDTRITEIDGSVGVKKSLVFYLGQQKEGKKTDWIMHEYTLQKEEFEACVLCKVYETSRGRNSRQNTRNEANTVSNDDGEQGTNPSTTRSQAEEDQALRFLPLPPPPRNIYCGSSSSSSSSNLAGQYDVHMTNHSDHALNNVSFPQCTWASNGLGAHPEVTTLAPMAHAQLENSIPLLHSMNIYDQDGYLLNENIEYYQSTAPYGFGAQSLPIYNNVSQPNNSGYGMAPTDTTEEEEELYLLHEPLPDFSFQIDDNAECVPFGMPVESAKPLNSVTPTDPVTQSTNPGGNSTTTPMDGH

>PmNAC096

MGGYQVPVGYRFTPSEEELLLHYLLPRVNGNDYPKGVVPDCDLYGTKEPWEIWRDFHHSSPDDQEDIYVFTTLKKKTPNGSRFCRTVGAAGTGVWKGEDSGKKIRACGNDIGTRKRFRYMNPGSPHDDRWIMLEFQLHESLVQVPNKETIVLCLVRKKETSGKSKLEERQNHEVDMASRGEIQAQHVHDDQQQHTDASTYDQLRFLDDYLMNDVGGEQNTMA

>PmNAC097

MSTTEESVLDLLPKFRFHPSEEEMVNLLKNKVEGQDSQAMPDEIVDKPWDLPDNESIQLFCRFLKRMMQGKASQSIPEIDVYKYEPWDLAELMFPDSPYQPRAWFSFSRPDYKYANSRRCNRASGKGFWKITGKPRQVKSRQLPKSVTCKKRTLTFHEGRVPKSRNTGWVRQEYYLTPTDPmDPGIAGCMASNSELEQAAEIHLISEAEEHLTYKELEHVLLGSGNQNDGEPGGFVSSDFDDMIQELCAKGGEYLDSPSPPERPHQHQLPQLGNVPVTSDYIGSYSNNQAAAINHMIPHPEDFLAYKELEHVLGTCNPDAGEPSGCVSSNMLQKLCAQLGEELDSPIPPPGPPELGNAPYVYIDECSSWPSPIGDNDSSLPNKNSIPTNYDSKPVSNTASNFENQTRISELPSPLRKSSPYAPKDKTHLFIIESVLDNLSDPVAIAKHYFPPNFHFMPQSPYKSLKYYRDILSETQPSSSGTSNTSTSVDGKSKSELQELARQLIIRASQMDDDDDDATPKSQSARVMSL

>PmNAC098

MISFSRAAAEEGDLSLPVGFRFRPTDEELVDYYLKNKVQGTDFHAEGIIDEIDILKFEPWDLVAKSLMKPDDENGFFFSQPEYTQKKNTKRSTEEGFWKITGREHDIRTRDTRRTVIGKKRILTFYIGHGRNSDKTNWVMHEYYIPKAHANQRDFVLCHCHLKKNVKKSDEIHTDVATTCDEGEPSTHNASDFEHPPmHDILEEYVDYNALQPmFAANDLDVEFAEFLETVLVEPDGGVTSDTDMEPFHGHGQAEMLSEVFYGSSQPSRDTGGIFRNQISPQAPSSVNVAPKPQTDRVQLKSDTSEGARGPRHQPGPIIDTSADEYYTKEKTRRRTNPPmKPEQPmYRRTAADLPQKQISLTNSSQGKKVAQGKNAEKEMIVISRAAEDDLSVPVGFRFHPTDEELVTHYLKKKLKGMDSHVSNIIREIDILKFEPWDLPKSSLLKSDDENWFFFSRPEYNKHKKNRTTQEGFWKITGREHAIKARDNRSVIGRKRILTFYRGRVRNSERTNWVMHEYYIPDDNPNAQRDFVLCRLKKNVKRSDENTDVAATCDEGETHNASDVENQPEDTWPPENLDYFERARDLLLASSPSNNDHNAFQPmFSAHDQEANFEDFLRTLIVEPQFGVISDRDREPVHHRVQSLQMRCEPQIPYELLQCGSSQSRRDTDVILHNQLSRQASSSVNVASKPGTYQREHRPQQQSGPIIVFRDTSADEYYTREKTRRITYPPEKPKEPEKPKPmAEKPKVPPYPRTAADFPQKQISITKSSIDKKVPQGSMEQTQNRTTPRNWKGSFITWQTSPLTSPPSVYIFNTVLGAILFLFCVRENAHSYICILLRNARCSDWNGSKRGHVYKFLRSQGFVSTYDTAHQYTDADAHKWVSHRHHKGNICGVDFIWLCNPNKSCKPLKTSWCEAVFGILRKGDSNSDFITSSAFCEALHQVNLIGQPLGLGFQETRDLWIQADVDSNGVLDYEEFKNRIWISTVSEEKENLNGSREESIRGTQDALGFNVKNAVVYPREAEKGIWPEDCTLSDHA

>PmNAC099

MIRFGRAAAEEGDLSLPVGFRFRPTDEELVDYYLKNKVQGMDFHAEGIIDEIDILKFEPRDLVAKSLMKPDDENGFFFSQPEYTPKNKTKRSTEEGFWKITGREHDIRTRDTRRTFIGKKRILTFYIGHGRNSDKTNWVMHEYYIPKAHPNANQRDFVLCHLKKNVKNSDEIHTDVATTCDEGEPSTRNAFDVENQPKHGMDIQEEDTQQPENLDYFEQAKDRWLASSCSNNDHNATFADNDSASELEERLRPFNTKPFHAGELSLEMLYELQHGSSQSIQDIDIMLHNQLSRQAASSVNVAPKPQTDRVQSEAGIALRKYGIRLRSDRIALRDTSADEYYTREKTRRITYPPEKAKEPEKPKPEPEKPKAPmYHRTAADLVEKQISITKSSIDKKVPQGSMEQTQNRTTPGNWKRSFITWQTSPLKSPPSVYIFNTVLGTILFLFCVREVVLYGEWS

>PmNAC100

MARLSRAAEEEEEGDLSVPVGFRFRPTDEELVNYYLKNKLEGMDSHAENIIDEIDILKFEPWDLPDKSLIKSDDENWFFFYKYKKGSRATKEGFWKITSKDRVIKARDNRTVIAKKRILTFYIGRVRKSTKTNWVIHEYYIPDDAHPNANKQRDFVLCHLKKNVKKSDENTDVATTCDEGETSTHNASDFENLPVHGMFQMQEDIHPRENLDYFERTRDRWLANSHRNNDHNAFQFDFEANDPEAEEFARSLLIDPQFDTDTDTEPIYVQSPERCEPQMFFEPQYVSSQSRRDTDVMFRNLLSRQASSSVNVAPQPQTSELQLQSGTSERTHRPQPKSISVLRDTSAVNVDALTFSVNRIQLATDDEYYTKERTRRRTHPPVGKLREIKLQQSKAKEPEERRTSVDLPQKQISITKSSIDGKVAQGKKTEKDVEQTQNRTTPSNWKGSFITWQTFPLTSPPSVYICNTVLGSILFYFCLREVVLYGKWC

>PmNAC101

MGSKCYRKTTEGDPVPLGFRFHPTEEELVDYYLKNKRQDRDFNVNHIPEIDICKYDPWEIPGLLFAEQDSPYMEWFFFSRRDYKYMNSNRSNRATPHGSWKITGKERMIRARGSNAVIGTKRTMTFYERGVLKSKKTNWVMHEYNLFESEASPDPQLAERDFVLYRMKKNPDKKDTSVFAEGEPSSYNWSNCEDQAAHVTPEDHSPSTLQSPmYIELGDVLQVNGFNGDCNDMQSPFGDNDYSVTHKNDFSTFDEVAYDMSQELYAQQDKNSYMLMPQSPTFRNLEDFTYINASDFGNQTANNMVLEAQDYHSFTLPSPIYSELGSVPHDDVYNNEWQSAYANFENQTTDERISEECPQPKENLRSILSASQLADYTSQSLMNTEEYQRRHCEIGIEGAAGMNWEGLLFTEPDSPYHDMEWFFFSLKDYKYSNSNRSNRATPKGYWKITGKERVIRARGSKFVIGRKRTLTFYEGRVPKSKKTNWVMHEYYLIEDEANSNPKLAKRDFVLCRLKKKLDKKDTSICAEGEPSNCNLSNCEDQVAAVVTPEDHSPSTLQSPmSIELGDVLQANDINEDCNEMQSPFGDNEYHVTDNNDISTCDEGEPHGFTMSDFKNEVADDMSRELYAQQERNLDPTYTELEGFMYIDASGFENQAGEQCAQPDKNLDPCFHPPQPHDYCSSTLQSPIYTELGSVPHANVYNDQWQSTYDGNGQVRNTIANFENQTTYERISEEYPQQEENLELIFHAPQLQDYTLQLQDYTLQPLMNTEVGDVLHDNNYIECNELQSPVWS

>PmNAC102

MPEIEYKPWNLSDNESISLFLRFLKQMMQGKASQACHIIPEIDVCKYEPSDLAELLFPDSPYQPRMWFSFSRPRYRSINSLRYNRATKKGFWKITGKPREIKSQQLSKSVTCKKRTLTFHEGRVSKSTKTDWVMQEYYLTQTEPGSIPNQLSDFVLCRMKNKSAHYESDNKKLKYASICDESADPGIGGCVASNSEDDQAPESNMIPEADRHLADKEPEHDLPGSGNHDAGEPDGCVSSDCDDMIQELSAQPGEYLDLHFPPPEPPEPGNASVPSPIGNNDSSLPYKNNITTNYDSKPVSNTASNFKNQTRDERTSEVYSQPEEDPESFLQLKLDDYTWLSRILQPEQGNLLHANNSIGCNELQSPYPETAALFPQSY

>PmNAC103

MSSGSSTTTTEAWVLDLLPEFRFRPSDEKMVNLLWKKINGKDYQVMPEINYKPWDLPDNESIPLFLRFLKQLMQGKASIIPEIDFYKHEPWDLAGAGLMFPDSPYQARTWFCFSRPDYKYVHSPRRNRLTEKGFWKITGQPREVKSRQLSKSLTCKKKTLTFHLGRPKKSSKTDWVKQEYYLTPTDPGSNPNQMSGLVLYRVKYRSADYESYNNCVLGTGNLNDGEPGGFVSSDFDDMIQEPCDQQEHLDSPSPPPEPPRPHQLPQLGNVHGSTGDYIASNSDNQAAAIHHMITDEEELLAYKELERVLLGSGNPDDGELRTCVSSDMLRELCAQLGEDLDSPFPPPGPPELGNAPDVCTDECSSWPTPIEDNDSSLPNKNNIPTNYYGKPVSNTASDFENQTKDERIPEVYSQSEENLQSFFRSLEEEDYTLPSPILYVEQGDVLHANNYIGCNESQYAALFPHSS

>PmNAC104

MSSISTMEEGDSIPVFLPGFRFYPTEEVLVSYYLKKKIEGKDSNFSHIIPEIDVCKHEPCDVPmFFEEADFPDQEMEWFFFSQPDYKYTNSTRCNRATDQGFYKITGKVREIKAGRSKVVIGKKRTLTFYEGRVPTAKKTNWIMHEYYLTNTELAQLGPNTNQQKDFVLCRLKNKSANYKKLKDDSICDELADTGSGGGIASNSEDDQAAGADMISEPLEHLASQEVGDVLNGNGSGLIENNDISICDDDQIDAWIFSDFDSQAAYDLLQEQYYAEPGENLDSPLPPPQPPQPPLPQDYCFSTQQSPLYTNQGNVPYVYDADCNRQQSPIGDRNSYLTHKNNISMNNQIEPVGSITYGVQNRATGGSNSEVYNNSTNDFEEPVSNITYNCNNGAPDERILEAFVPRCIVLEVALYRFCCCMAAGVSMYVTITRHYCDVCGYWNGSFEVPSLMK

>PmNAC105

MSSSSSTTTDAWVLDLLRGLRYHPPDEEMVNLLKKRMEGSQARPIIPDIDFYNYEPWELAGRMLPDSPYQPRMWFSFSRHHHKYANSPRCNRLTEKGFWKITGKPREIKSRQLSESVTCKKRTLTFHLGRPSKQSKTGWVKQEYYLTSTAPGSNLSGLVLFRMKNKSADYKSDNKQQQDVAIYDESADPmIGGDVASNSQDDQAAANHKILEAEAEQHLLGSGNHNDGEPGGFVSSDFDIMQELCAQLGLEPPQLGNVPGTGDYLASNSDNQAAAIHPMTTEEEELLAFKELERVLLGSGNPDNGEPRSCVSSDMRQESCAQPGEDLDSPVPPPGPPELGNAPDVYNDSSLPNKNNIPNNYDSKPISNTASDFENQAKDERIPEVYSQSEENLQLFFRSLEVENYTLPSSILYVEQGDVLHANNYIGCNESQYAALFPHSS

>PmNAC106

MEEGDSVPVVLPGFRFYPTEEVLVGYYLKKKIEGKDSNFSHIIPEIDVCKHEPCDIPmFFEEPDFPDHEMEWFFFSQPDFKYTNSTRCNRATDQGFYKITGKVREIKARRSKAVIGKKRTLTFYEGRVPKAKKTDWIMHEYYLTSTELAQLGPNPNQQKDFVLCRLKNKSANYKKVKADFGGCIASNSEDDQAAATDMISEPLEHLASQEVGDVLNGNGDHDECTESPNGTGLIDNNDISSCDDDEIDTWIFYDFDNQAACDLFQEQYCAEPGENLDSLLPPPQPPQPPLPPLPQDYCSSTQQSPIYTNQGNVPYVYDGDCNRQQSPIGDRNSYLTHKNNMSMNNQIEPVSNITYGVQNRATGESNSEVNNNSTNDFKEPVSIITCNFNNGAPNERISKACSQPKENLGSCFDPFQLQDFTLLSPMNLELGDFEHGNKLYWHAMSCNL

>PmNAC107

MGGGGGTTDERVSVPMGLLPGIKFSPTDKELLSFYLKKKIAGEDSEFSNIIPEINLREHEPRDLPTEFFFSEPNYMSPNGDSCYRTTNEGFYKSTGKPREIEDELSKAVIGEKRYMPFYEGRAPNHKLTKYVLHQFSLTKTELAKLAPNREFVLNHLTIKKGSTRACNPKPNCKKRKYDSNCDKLAEHGIPGYIASNYKDDQAAAAANMIPEPEEHLADEDVLLGSENRNECAESESANGSRLINNNDSSTLAVGSPGGCFLSGYGNVAAYDPFREVSFQPEGNLISPLHPSSLRDIPLRSPVHTGLPGDFMCANNSILIGMQL

>PmNAC108

MSSSGGTTDGGVSVPMGLLPGFRFSPTEEELLSFYLKKKIAGEDSELSDIIPEINVCEREPRDLPEYFFSKPNYMNTNSNRCSRTTDEGYYKSTGQVREVKAELSQAVIGNKRILPFLEGRAPKGKITKYVMHEFSLPKTKQAQLGPNPNQQREFVLNHLTTKSVNYKKRKYDSICDKVAEPGIASNYKDDQAAAAANMILEPKEHLADKDVLLGSEKCNEGAESESVNGSCLIDNNDISTLDVGLLGGCFSSGSDNLAVNDSFHEVSFQPERNLISPFHPSSLRDIPLISPVHTGLLGDVVIHSCLHTREVLREESDCKAVSQDKVSDAAECCFAFHLKTKSWKHTV

>PmNAC109

MGTFKQAVKFVVLPmLPCSFFSAHFLLSPQHSSTSLFLANIPTRKLLFKKFIDDCWECDPNWPDNRQSLADCAIGFGQYALGGKGGEYYIVTDSSNDDAVNPRPGTLRYAVIQTEPLWIVFPGNMLIKLSQELIFNSCKTLDCRGANVHIVGCGCITWQYISNVIIHNVHIHHCYPSVDKLRSFLRQLPLFSNAFLVGGAEDFFIIELADQVDIFFTGQLQKLKSVVLSGGYEDDEDHGEWFLYTGSGGRDLSGNKRTNKAHTFDQKLELRNQALRESCLQGYPVRVVSGGGGGGGGGGTTGEGVSGKKGLPGVKFFFPTEQELVGYLTNQIEGKDSEFRDLIPVIDNVCEREPRDLPmFFFSQPDYKYKNSKRCNRSTDEGFYKSTGKVREIKAEQSQAVIGNKRILSYYEGRAPQAKKTKHVMHEYSLTQTKLAQLGAQNNQQRELVLCHLTNKSAKSAKLKDDSICGDELAEPmIVGEIAYSKDVQAAATDMIQGPDEHLKDKDALLGTENRNECAEPSCADLGENMDSTFLSPEQLAATSATTPTSATSFGGFGVGSPDGFFLSDIDDLLKQPSCADLGENMDSTFLSPEQLAATSATTPTSATSFGGFGVGSPDGFLLPDIDDMLKQPSCADLGENMDLTFLPPEQQAATSATTPTSATSFGGFGVGSPDGFLLSDIDDLLKQPSCADLGENMDSTFLPPEQLAATSATTPTSATSFGGFGAGSPDGFLLPDIDDLLKEVNF

>PmNAC110

MAGPSWLVDKNRIATKIKSASGTCDPERIKWQSNPTRACPNCQYTIDNSDVAQEWPGLPKGVKFDPTDQEIIWHLIAKSGAEDLKPHPFIDEFIITVDDDEGICCSHPHKLPSVKQDGSASHFFHRAIKAYNTGTRKRRKIHDGDGDVRWHKTGRTKPVMLDGVQRGCKKIMVLYMSTVGGGKPKKTNWVMHQYHLGTEEDEKDGEYVISKIYYQQQQVKQADKTDQDLPENFDLVITKVDPVTPKSVTPEPPRTTSRDPFPQHPEMDQAEDEVHPEMDHAEDEVHPEFEKPDHYDQHNVENEADEVINNTENNAEEDPKWWDSESQNLLDSQQLVEGLSLCDDLLQSQSPIRAGHENGEPmQVKPRLADYAKLGPENLKKDLDECQNIVLDPmNIMLEDTPPDFRLSQLEFGSQESFLSWGVKLAD

>PmNAC111

MAVLSMDSLPLGFRFRPTDEELINHYLKLKINGRNSEVQVIPEIDVCKWEPWDLPKLSVIKSDDQEWFFFCPRDRKYPNGHRSNRATDAGYWKATGKDRTIKSRQCKSASNSTGQVGMKKTLVFHRGRAPRGERTSWIMHEYRATQKDLDGTAPGQGAFVLCRLFHKPEEKADVLKYDEVEQTGLSPTTTKYSPDETSSDVVQETATPDMQGEKQSESIMRWWNDKSDNMSPDALPPVPGDSYMASDVEDPGAEETGIQGHLLKEENQVFDEPFGGQIDCKVFSPLQSLINSELEHYVGSPFTSDFGIYNNGFHFQDGTCEQDVAFPEFLDEFGINPYESSCEESTSQKNLVVGNETYLSGQSCMLQTTPPGNSCLNGAWDNTDTNIAQHDQEGRASGLYSKQFDAEDLLQKTSLGYYQAEAQASLDNQKPRMGNMADNYFPPSAFAEQFLVSSVNDTFNSSDGSTSWKNFDNHSGDHGGGTAIKIRARHLQQLQNSGNFVDQGCAPRRLLLCVDKPSSGSIANGNMGDANYRKEEDEVQSAITEARDDIRQSPSSDEQEEEHAIFNNGEEFSSRKDSVNHGYDQVGRTRIRIKTRQPQQQSNSENYVTQGTAPRRIRLQMNISTGSVVDSNVRDRDDVEEDEVQSTNTDAREAIQQSHTSDEQEKEHAKINNKEELTSRKNSVNYGSDIVGITGIRIKARQPQQPLKSDESVTQGTAPRRIRLQMNPVADSNLREPTPGKEDEVQSTINETRESTQKIPDSDEHDAECHLSKLEVNGKMAEDSPmEMVDKSRETAEEAAGKLKLRTGSDSSLHSNHTGLRVRFLILRSLPFVSSYPLPPPPPLIGAPmAA

>PmNAC112

MEREQQTSDNIQLPmGFRFHPSDEELIVHYLKNKVTSSPLPmTIITELDLYKYNPWELPmKASFGEEEWYFFTPRDRKYPNGSRPNRAAGLGYWKATGTDKHIFSSCGTKSIGVKKALVFYTGHPPKGVKTEWIMNEYRLLDTTMWSSKQKGSMRLDDWVLCRVRQKCNSSRSIWEDQNSPPSYKLGAYTKQADETCSKDTNPSIEMVRNYLYKDCPMLPYIFASPELPYTKTTSSISFQGSGDTKSCTKIHENDSLNKNNGPLLASSLESLINPFKRKPmAEGNGHHQSFVTPSKRICSREYQEEVSTSIGRDGCAMNSWGVDQSGSAENNFNADQWSSMIQYQELSQLIGFQCK

>PmNAC113

MAPCESVVNLPVGFIFCPRDDQLLGYYLLNKDSGKPFQYDNVIPEMDLYGKIEPWDIWHEYGGHNLAKGEDLYFFTKLKSLSDKDSRVARTIGSGTWKGENSGTTVSDPKNKKNNLGIWKRFHYENPKSVQDGCWIMHEYSLHSSLVKPKPNSTNRFVLCRIRKNGRGKKKLRTAEEDNETNNPVQSQNTRQRPQQLTSLSEATGIGGSVPFGSEEDQVLNFTDYDSLYHLINCDDVPPQSSTAQFMGM
