# Supplementary figures and images for: Genome-Wide Analysis of the NAC Transcription Factor Gene Family Reveals Differential Expression Patterns and Cold-Stress Responses in the Woody Plant Prunus mume
Source: Genes (Basel). 2018 Oct 12;9(10):494. doi: 10.3390/genes9100494 (PMC6209978; doi:10.3390/genes9100494)

# Schematic diagram of PmNAC protein motif

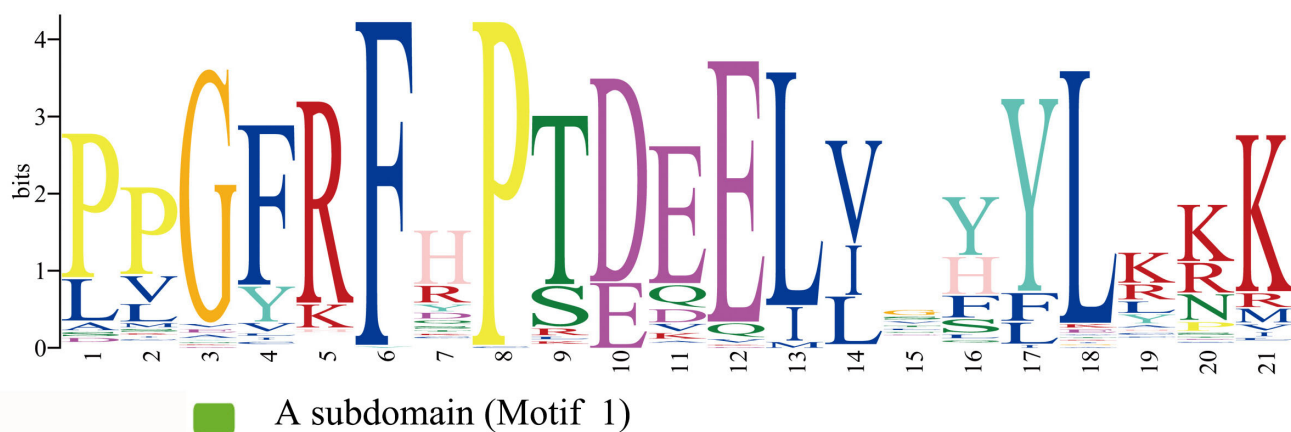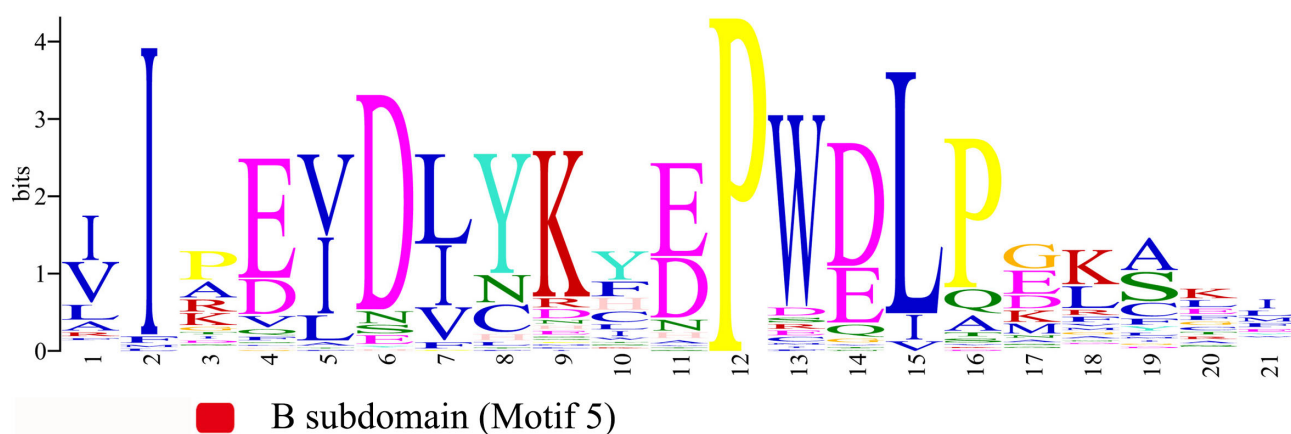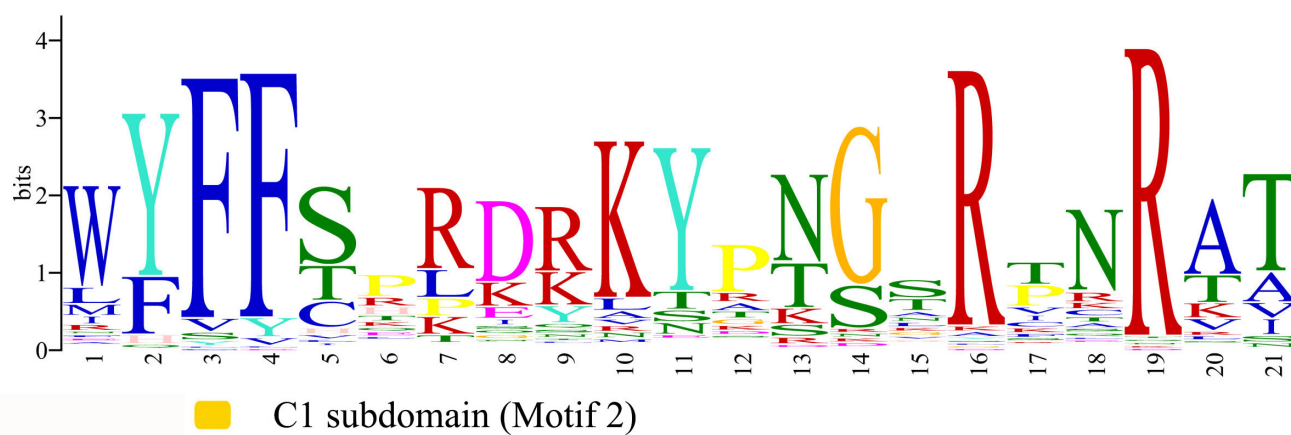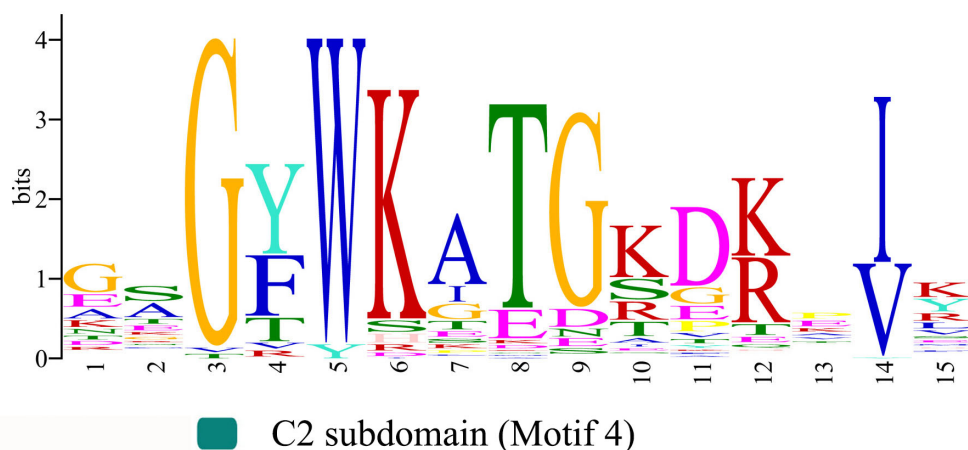

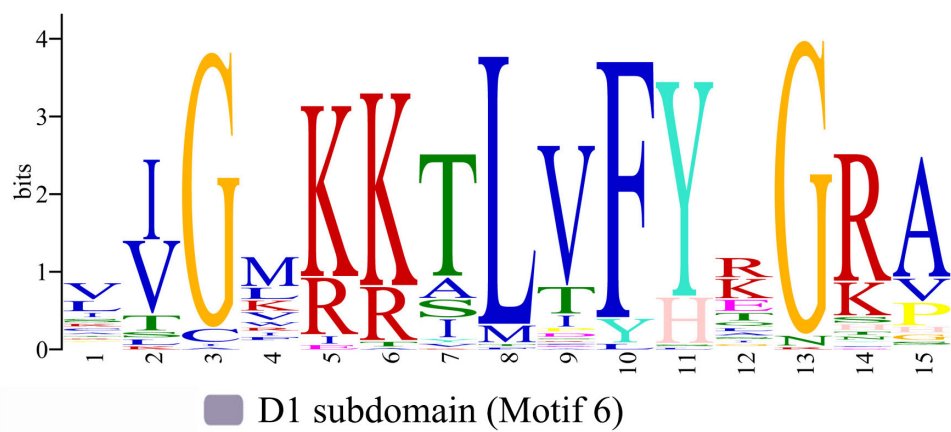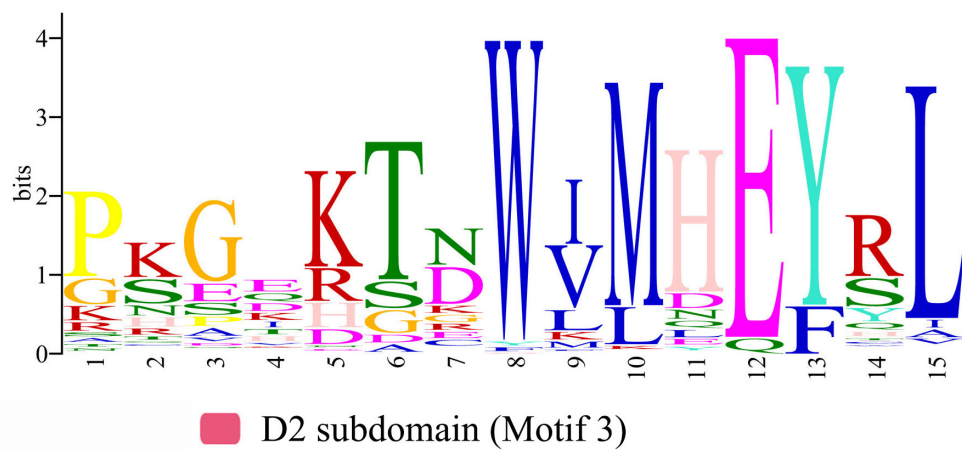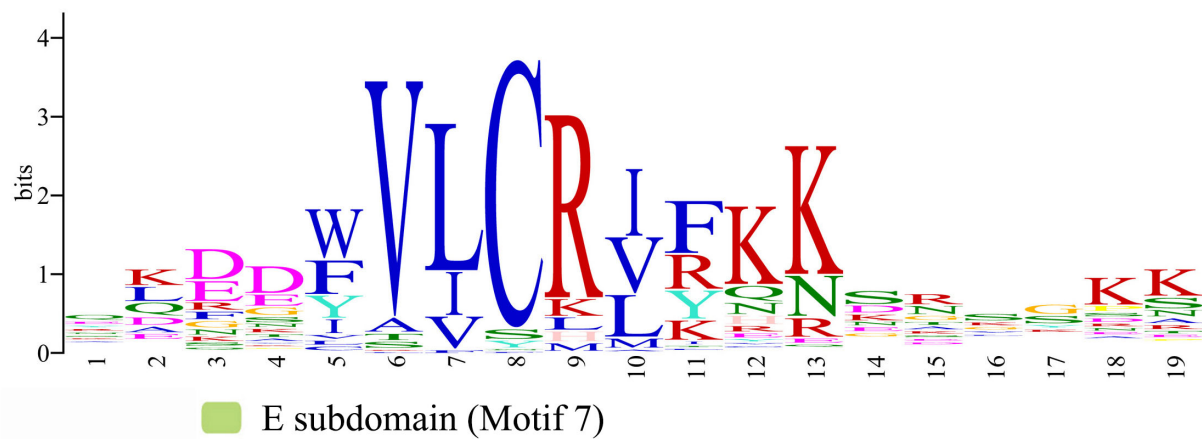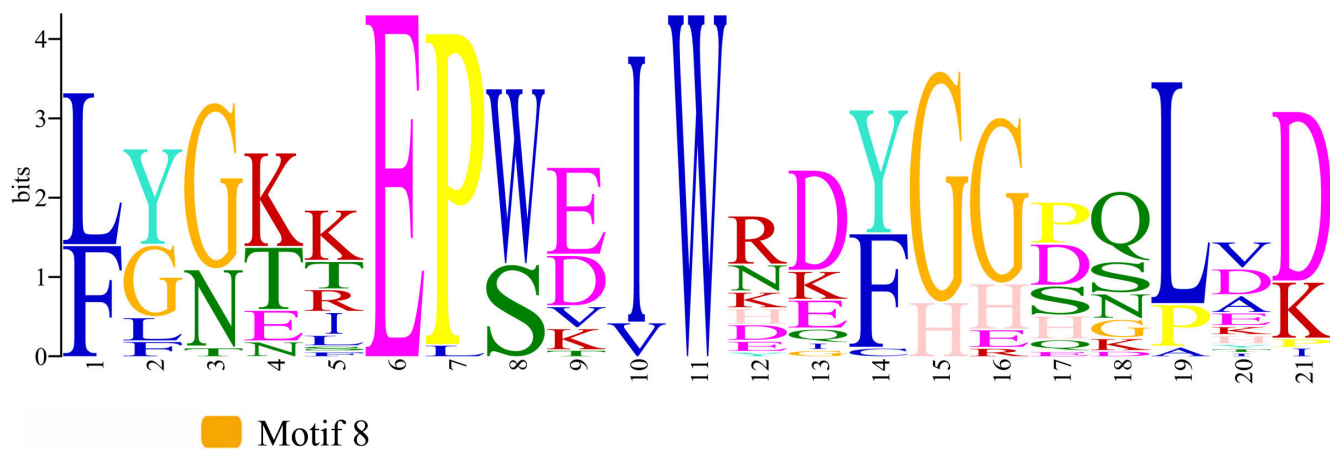

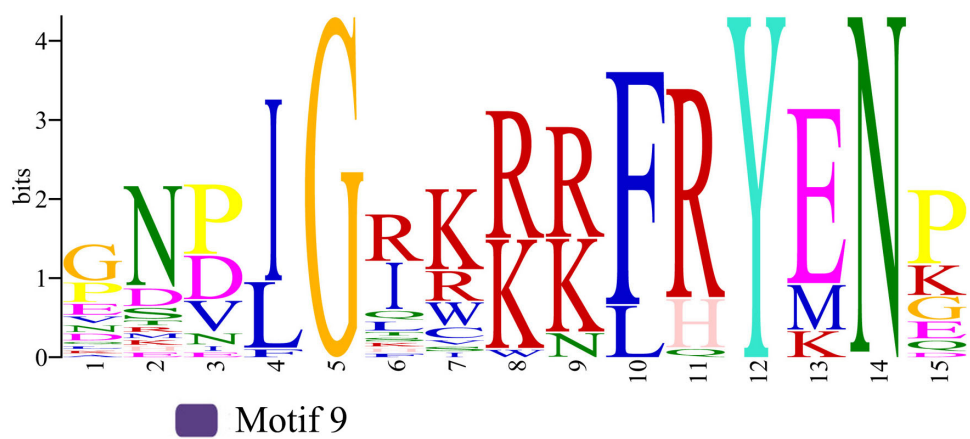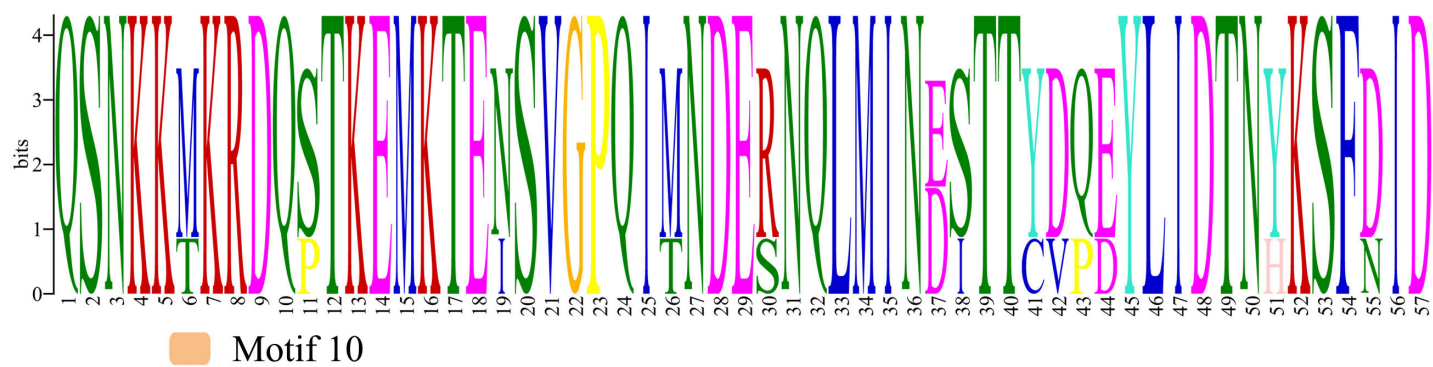

Supplement: Supplementary file 1 [file genes-09-00494-s001.zip › Supplementary materials/Supplementary File S1 .pdf]
